# Conditional cooperation with longer memory

**DOI:** 10.1073/pnas.2420125121

**Published:** 2024-12-06

**Authors:** Nikoleta E. Glynatsi, Ethan Akin, Martin A. Nowak, Christian Hilbe

**Affiliations:** ^a^Max Planck Research Group Dynamics of Social Behavior, Max Planck Institute for Evolutionary Biology, Plön 24306, Germany; ^b^Department of Mathematics, The City College of New York, New York, NY 10031; ^c^Department of Mathematics, Harvard University, Cambridge, MA 02138; ^d^Department of Organismic and Evolutionary Biology, Harvard University, Cambridge, MA 02138

**Keywords:** evolutionary game theory, direct reciprocity, evolution of cooperation, prisoner’s dilemma

## Abstract

Direct reciprocity is a wide-spread mechanism for the evolution of cooperation. In repeated interactions, players can condition their behavior on previous outcomes. A well-known approach is given by reactive strategies, which respond to the coplayer’s previous move. Here, we extend reactive strategies to longer memories. A reactive-*n* strategy takes into account the sequence of the last *n* moves of the coplayer. A reactive-*n* counting strategy responds to how often the coplayer cooperated during the last *n* rounds. We derive an algorithm to identify the partner strategies within these strategy sets. Partner strategies are those that ensure mutual cooperation without exploitation. We give explicit conditions for all partner strategies among reactive-2, reactive-3 strategies, and reactive-*n* counting strategies. To further explore the role of memory, we perform evolutionary simulations. We vary several key parameters, such as the cost-to-benefit ratio of cooperation, the error rate, and the strength of selection. Within the strategy sets we consider, we find that longer memory tends to promote cooperation. This positive effect of memory is particularly pronounced when individuals take into account the precise sequence of moves.

## Significance

In repeated interactions, people tend to cooperate conditionally. They are influenced by whether others cooperate, and react accordingly. Direct reciprocity is based on repeated interactions between two players. Nice strategies are those that are never the first to defect. Consequently, they never seek to exploit the other. Partner strategies are nice strategies which can sustain full cooperation as a Nash equilibrium. If you interact with such a partner then you maximize your own payoff by full cooperation. Therefore, partners resolve social dilemmas. Here, we characterize partners among those strategies that react to an opponent’s behavior during the last *n* interactions. Such players can sustain cooperation in an equilibrium, even if their opponent uses a longer memory strategy.

**Fig. 1.**
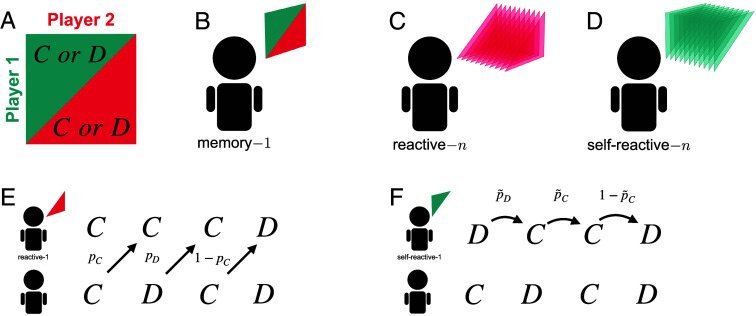
The repeated prisoner’s dilemma among players with finite memory. (*A*) In the repeated prisoner’s dilemma, in each round two players independently decide whether to cooperate (*C*) or to defect (*D*). (*B*) When players adopt memory-1 strategies, their decisions depend on the entire outcome of the previous round. That is, they consider both their own and the coplayer’s previous action. (*C*) When players adopt a reactive-*n* strategy, they make their decisions based on the coplayer’s actions during the past *n* rounds. (*D*) A self-reactive-*n* strategy is contingent on the player’s own actions during the past *n* rounds. (*E*) To illustrate these concepts, we show a game between a player with a reactive-1 strategy (*Top*) and an arbitrary player (*Bottom*). Reactive-1 strategies can be represented as a vector $p=(p_{C},p_{D})$. The entry *p*_*C*_ is the probability of cooperating given the coplayer cooperated in the previous round. The entry *p*_*D*_ is the cooperation probability after the coplayer defected. (*F*) Now, the *Top* player adopts a self-reactive-1 strategy, $\tilde{p}=\left({\tilde{p}}_{C},{\tilde{p}}_{D}\right)$. Here, the player’s cooperation probability depends on its own previous action.

To a considerable extent, human cooperative behavior is governed by direct reciprocity (1, 2). This mechanism for cooperation can explain why people return favors (3), why they show more effort in group tasks when others do (4), or why they stop cooperating when they feel exploited (5, 6). The main theoretical framework to describe reciprocity is the repeated prisoner’s dilemma (7–12). This game considers two individuals, referred to as players, who repeatedly decide whether to cooperate or to defect with one another (Fig. 1*A*). Both players prefer mutual cooperation to mutual defection. Yet given the coplayer’s action, each player has an incentive to defect. One common implementation of the prisoner’s dilemma is the donation game. Here, cooperation simply means to pay a cost *c* > 0 for the coplayer to get a benefit *b* > *c*. Despite the simplicity of these games, they can give rise to remarkable dynamical patterns. These patterns have been explored in numerous studies (13–32). Some of this literature describes how the evolution of cooperation depends on the game parameters, such as the benefit of cooperation, or the frequency with which errors occur (33–36). Others describe the effect of different learning dynamics (37, 38), of population structure (39–42), or of the strategies that players are permitted to use (43).

Strategies of the repeated prisoner’s dilemma can vary in their complexity. While some are straightforward to implement, like always defect, many others are more sophisticated (44, 45). One way to quantify a strategy’s complexity is to resort to the number of past rounds that the player needs to remember. Unconditional strategies like “always defect” (ALLD) or “always cooperate” (ALLC) are said to be memory-0. Strategies that only depend on the previous round, such as “Tit-for-Tat” (7, 46) or “Win-Stay Lose-Shift” (20, 21), are memory-1 (Fig. 1*B*). Similarly, one can distinguish strategies that require more than one round of memory, or strategies that cannot be implemented with finite memory (10).

Traditionally, most theoretical research on the evolution of reciprocity focuses on memory-1 strategies (21–31). Although one-round memory can explain some of the empirical regularities in human behavior (47–51), people often take into account more than the last round (52, 53). In experiments, longer memory seems particularly relevant for noisy games, where people occasionally defect because of unintended errors (54). However, a formal analysis of strategies with more than one-round memory is nontrivial, for two reasons. First, as the memory length *n* increases, strategies become harder to interpret. For example, because two consecutive rounds of the prisoner’s dilemma allow for 16 possible outcomes, memory-2 strategies need to specify 16 conditional cooperation probabilities (55, 56). Although some of the resulting strategies have an intuitive interpretation, such as “Tit-for-Two-Tat” (7), many others are difficult to make sense of. Second, the number of strategies, and the time it takes to compute their payoffs, increases dramatically in *n*. For example, for memory-1, there are 2^4^ = 16 deterministic strategies (strategies that do not randomize between different actions). When both players adopt memory-1 strategies, computing their payoffs requires the inversion of a 4 × 4 matrix (9). After increasing the memory length to memory-2, there are 2^16^ = 64,536 deterministic strategies, and payoffs now require the inverse of a 16 × 16 matrix.

There have been various approaches to tackle this problem. Some studies describe the strengths of particular strategies with more than one-round memory (57–60). Others explore the properties of entire strategy classes, such as “zero-determinant strategies” (61, 62) or “reactive learning strategies” (19). Stewart and Plotkin (63) characterize a set of memory-*n* strategies that is evolutionary robust. They show that for larger *n*, the volume of robust cooperative strategies exceeds the volume of strategies that lead to mutual defection. However, they do not provide an explicit description of the memory-*n* Nash equilibria. We give a more detailed account of these approaches in our *SI Appendix*.

To make further progress, we focus on an easy-to-interpret subset of memory-*n* strategies, the reactive-*n* strategies. Capturing the basic premise of conditional cooperation, they only depend on the coplayer’s actions during the last *n* rounds (Fig. 1 *C* and *E*). We show that within the reactive-*n* strategies, an explicit characterization of all Nash equilibria becomes feasible. Our results rely on a central insight, motivated by previous work of Press and Dyson (25): If one player adopts a reactive-*n* strategy, the other player can always find a best response among the deterministic self-reactive-*n* strategies. Self-reactive-*n* strategies are remarkably simple. They only depend on the player’s own previous *n* moves (Fig. 1 *D* and *F*). Based on this insight, we study all reactive-*n* strategies that sustain full cooperation in a Nash equilibrium (the so-called partner strategies). We provide a full characterization for *n* = 2 and *n* = 3. Even stronger results are feasible when we restrict attention to so-called counting strategies. Such strategies only react to how often the coplayer has cooperated in the last *n* rounds (irrespective of the exact timing of cooperation). For the donation game, we characterize the partners among the counting strategies for arbitrary *n*. The resulting conditions are straightforward to interpret: For every defection of the coplayer in memory, the focal player’s cooperation rate needs to drop by c/(nb). To further assess the relevance of partner strategies for the evolution of cooperation, we conduct extensive simulations for n∈{1,2,3}. Our findings indicate that the evolutionary process strongly favors partner strategies and that these strategies are crucial for cooperation.

Overall, our results provide important insights into the logic of conditional cooperation when players have more than one-round memory. We show that partner strategies exist for all repeated prisoner’s dilemmas and for all memory lengths. These findings also allow us to reinterpret existing results on strategies with shorter memory. For example, we find that the well-known strategy Generous Tit-for-Tat (GTFT, see refs. 64 and 65) is just one instance of a more general strategy class. The same principles that make GTFT sustain cooperation within the reactive-1 strategies, allow us to construct partners within the reactive-*n* strategies.

## Results

### Model and Notation.

We consider a repeated game between two players, player 1 and player 2. Each round, players can choose to cooperate (*C*) or to defect (*D*). If both players cooperate, they receive the reward *R*, which exceeds the (punishment) payoff *P* for mutual defection. If only one player defects, the defecting player receives the temptation *T*, whereas the cooperator ends up with the sucker’s payoff *S*. We assume payoffs satisfy the typical relationships of a prisoner’s dilemma, T>R>P>S and 2R>T+S. Therefore, in each round, mutual cooperation is the best outcome for the pair, but players have some incentive to defect. The players’ aim is to maximize their average payoff per round, across infinitely many rounds. To make results easier to interpret, it is sometimes instructive to look at a particular variant of the prisoner’s dilemma, the donation game. Here, cooperation means to pay a cost *c* > 0 for the coplayer to get a benefit *b* > *c*. The resulting payoffs are R=b−c,S=−c,T=b,P=0. For simplicity, we focus on the donation game in the following. However, most of our findings are straightforward to extend to the general prisoner’s dilemma (or to other repeated 2 × 2 games, see *SI Appendix*).

We consider players who use strategies with finite memory. To describe such strategies formally, we introduce some notation. The last *n* actions of each player i∈{1,2} are referred to as the player’s *n*-history. We write this *n*-history as a tuple $h^{i}=\left(a_{-n}^{i},\ldots ,a_{-1}^{i}\right)\in {\left\{C,D\right\}}^{n}$. Each entry $a_{-k}^{i}$ corresponds to player *i*’s action *k* rounds ago. We use *H*^*i*^ for the set of all *n*-histories. This set contains $|H^{i}|=2^{n}$ elements. Based on this notation, we can define a reactive-*n* strategy for player 1 as a vector $p={\left(p_{h}\right)}_{h\in H^{2}}\in {\left[0,1\right]}^{2^{n}}$. The entries $p_{h}$ correspond to player 1’s cooperation probability in any given round, contingent on player 2’s actions during the last *n* rounds. The strategy is called pure or deterministic if any entry is either zero or one. We note that the above definition leaves player 1’s moves during the first *n* rounds unspecified. However, in infinitely repeated games without discounting, these initial moves tend to be inconsequential. Hence, we neglect them in the following.

For *n* = 1, we recover the classical format of reactive-1 strategies (9), $p=(p_{C},p_{D})$. Here, *p*_*C*_ and *p*_*D*_ are the player’s cooperation probability given that the coplayer cooperated or defected in the previous round, respectively. This set contains, for example, the strategies of unconditional defection, ALLD =(0,0), and Tit-for-Tat, TFT =(1,0). The next complexity class is the set of reactive-2 strategies, $p=(p_{\mathrm{CC}},p_{\mathrm{CD}},p_{\mathrm{DC}},p_{\mathrm{DD}})$. In addition to ALLD and TFT, this set contains, for instance, the strategies Tit-for-Two-Tat, TF2T =(1,1,1,0) and Two-Tit-for-Tat, 2TFT=(1,0,0,0). Similar examples exist for *n* > 2. When both players adopt reactive-*n* strategies (or more generally, memory-*n* strategies), it is straightforward to compute their expected payoffs, by representing the game as a Markov chain. The respective procedure is described in *SI Appendix*.

Herein, we are particularly interested in those reactive-*n* strategies that sustain full cooperation. Such strategies ought to have two properties. First, they ought to be nice, meaning that they are never the first to defect (7). This property ensures that two players with nice strategies fully cooperate. In particular, if $h_{C}$ is a coplayer’s *n*-history that consists of *n* bits of cooperation, a nice strategy needs to respond by cooperating with certainty, $p_{h_{C}}=1$. Second, the strategy ought to form a Nash equilibrium, such that no coplayer has an incentive to deviate. Strategies that have both properties are called partner strategies (66) or partners. The partners among the reactive-1 strategies are well known. For the donation game, partners are those strategies with $p_{C}=1$ and $p_{D}\leq 1-c/b$ (29). However, a general theory of partners for *n* ≥ 2 is lacking. This is what we aim to derive in the following. In the main text, we provide the main intuition for our results; all proofs are in *SI Appendix*.

### An Algorithm to Identify Partners Among Reactive-*n* Strategies.

It is comparably easy to verify whether a reactive-*n* strategy **p** is nice. Demonstrating that the strategy is also a Nash equilibrium, however, is far less trivial. In principle, this requires uncountably many payoff comparisons. We would have to show that if player 2’s strategy is fixed to **p**, no other strategy *σ* for player 1 can result in a higher payoff. That is, player 1’s payoff needs to satisfy $\pi^{1}\left(\sigma ,p\right)\leq \pi^{1}\left(p,p\right)$ for all *σ*. Fortunately, this task can be simplified considerably. Already Press and Dyson (25) showed that it is sufficient to test only those *σ* with at most *n* rounds of memory. Based on two insights, we can even further restrict the search space of strategies *σ* that need to be tested.

**Fig. 2.**
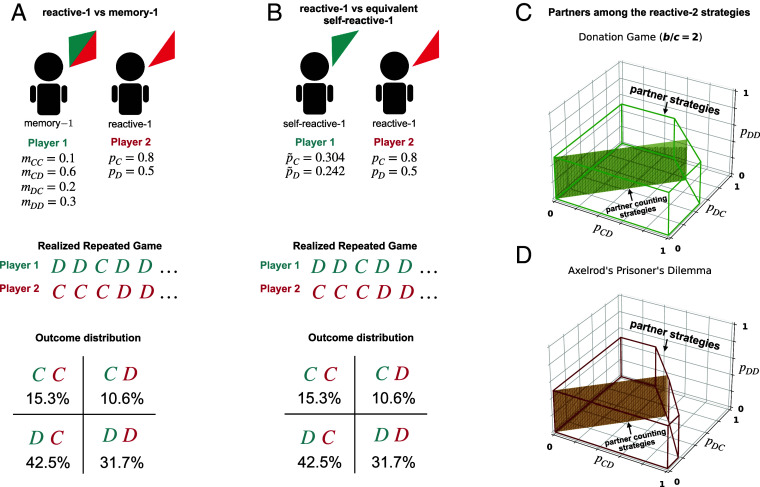
Characterizing partners among the reactive-*n* strategies. (*A* and *B*) To characterize the reactive-*n* partner strategies, we prove the following result. Suppose the focal player adopts a reactive-*n* strategy. Then, for any strategy of the opponent (with arbitrary memory), one can find an associated self-reactive-*n* strategy that yields the same payoffs. Here, we show an example. Player 1 uses a reactive-1 strategy against player 2 with a memory-1 strategy. Our result implies that player 2 can switch to a well-defined self-reactive-1 strategy. This switch leaves the outcome distribution unchanged. In both cases, players are equally likely to experience mutual cooperation, unilateral cooperation, or mutual defection in the long run. (*C*) Based on this insight, we can explicitly characterize the reactive-2 partner strategies (with $p_{\mathrm{CC}}=1$). Here, we represent the corresponding conditions in Eq. **1** for a donation game with b/c=2. Among the reactive-2 strategies, the counting strategies correspond to the subset with $p_{\mathrm{CD}}=p_{\mathrm{DC}}$. Counting strategies only depend on how often the coplayer cooperated in the past, not on the timing of cooperation. (*D*) Similarly, we can also characterize the reactive-2 partner strategies for the general prisoner’s dilemma. Here, we use the payoff matrix of Axelrod (7).

First, suppose player 1 uses some arbitrary strategy *σ* against player 2 with reactive-*n* strategy $p={\left(p_{h}\right)}_{h\in H^{1}}$. Then we prove that instead of *σ*, player 1 may switch to a self-reactive-*n* strategy $\tilde{p}$ without changing either player’s payoffs. When adopting a self-reactive strategy, player 1 only takes into account her own actions during the last *n* rounds, $\tilde{p}={\left({\tilde{p}}_{h}\right)}_{h\in H^{1}}$. In particular, if *σ* is a best response to **p**, then there is an associated self-reactive strategy $\tilde{p}$ that is also a best response. This result follows the same intuition as a similar result of Press and Dyson (25): If there is a part of the joint history that player 2 does not take into account, player 1 gains nothing by considering that part of the history. In our case, because player 2 only considers the last *n* actions of player 1, it is sufficient for player 1 to do the same. Fig. 2 *A* and *B* provides an illustration. There, we depict a game in which player 1 adopts a memory-1 strategy against a reactive-1 opponent. Due to the above result, we can find an equivalent self-reactive-1 strategy for player 1. While that self-reactive strategy is simpler, on average it induces the same game dynamics. Hence, it results in identical payoffs.

The above result guarantees that for any reactive-*n* strategy, there is always a best response among the self-reactive-*n* strategies. In a second step, we prove that such a best response can always be found among the deterministic self-reactive-*n* strategies. This further reduces the search space for best responses, from an uncountable set to a finite set of size $2^{2^{n}}$. For *n* = 2, this leaves us with 16 self-reactive strategies to test. For *n* = 3, we end up with (at most) 256 strategies. While this may still appear to be a large number, many of the different strategies impose redundant constraints on partner strategies. This redundancy further reduces the number of conditions a partner needs to satisfy.

### Partners Among the Reactive-2 and the Reactive-3 Strategies.

To illustrate the above algorithm, we first characterize the partners among the reactive-2 strategies. To this end, we note that it is straightforward to compute the payoff of a specific self-reactive-2 strategy against a general reactive-2 strategy **p** (*SI Appendix*). By computing the payoffs of all 16 pure self-deterministic strategies $\tilde{p}$, and by requiring $\pi^{1}\left(\tilde{p},p\right)\leq \pi^{1}\left(p,p\right)$ for all of them, we end up with only three conditions. Specifically, we prove that **p** is a partner if and only if

[1] $$p_{\mathrm{CC}}=1,\;\frac{p_{\mathrm{CD}}+p_{\mathrm{DC}}}{2}\leq 1-\frac{1}{2}\cdot \frac{c}{b},\;p_{\mathrm{DD}}\leq 1-\frac{c}{b}.$$

**Fig. 3.**
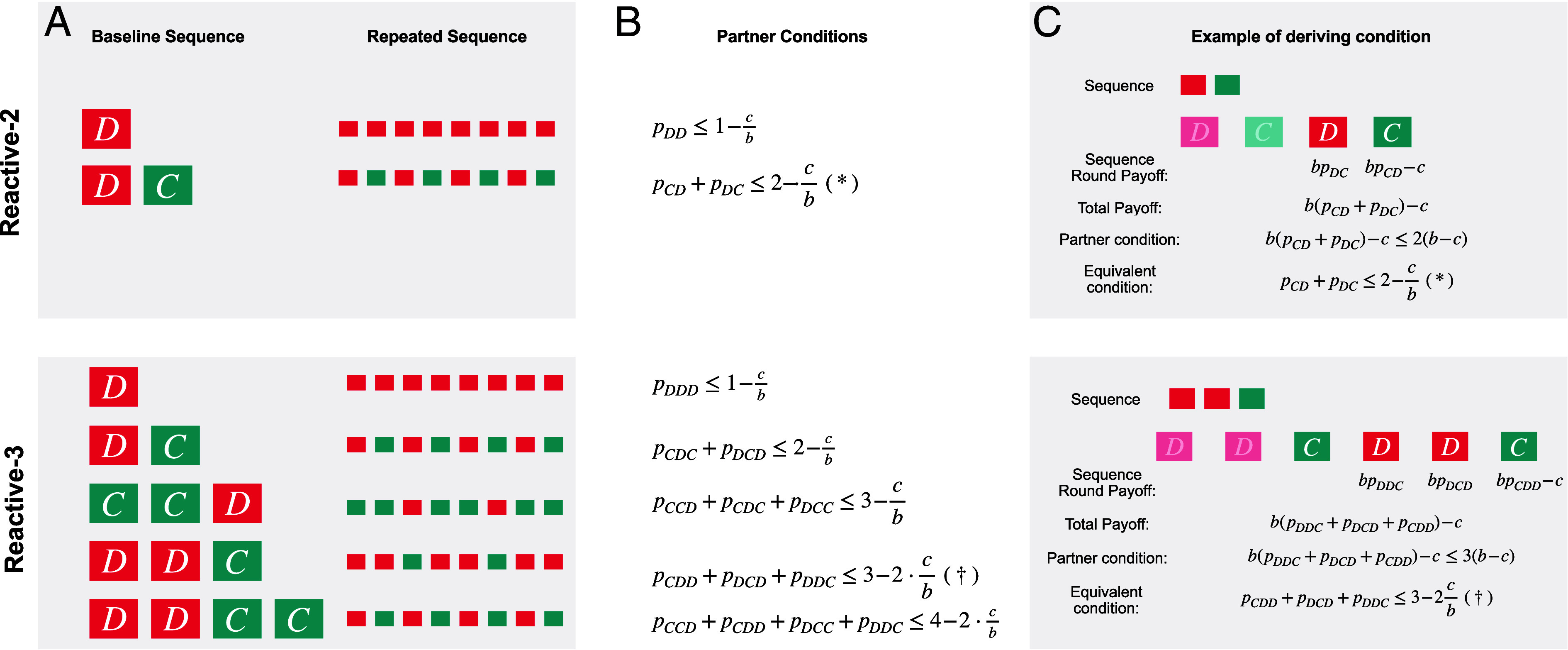
Conditions for partners among reactive-2 and reactive-3 strategies. (*A*) Pure self-reactive strategies generate simple repetitive sequences of actions that are independent of the coplayer. For example, in the case of *n* = 2, the pure self-reactive strategy $\tilde{p}=\left(0,1\right)$ generates the indefinitely repeated alternating sequence *DC*. (*B*) For a nice reactive strategy **p** to be a partner, all of these self-reactive strategies need to achieve at most the mutual cooperation payoff against **p**. This leads to necessary conditions for **p** to be a partner, which we show here for *n* = 2, and *n* = 3. Interestingly, we prove that these necessary conditions are also sufficient, see *SI Appendix*. (*C*) To derive the conditions, we consider the average payoff of each repetitive sequence. In the *Top* panel, we illustrate an example for *n* = 2. Here, the repetitive sequence *DC* plays against the reactive strategy $p=(1,p_{\mathrm{CD}},p_{\mathrm{DC}},p_{\mathrm{DD}})$. In odd rounds, the sequence player receives a benefit *b* with probability *p*_*DC*_, without paying any cost. In even rounds, the player receives the benefit *b* with probability *p*_*CD*_, while paying a cost *c*. Over the course of two consecutive rounds, the player thus receives $(p_{\mathrm{DC}}+p_{\mathrm{CD}})b-c$. This payoff needs to be smaller or equal than what a partner strategy achieves against itself, which is 2(b−c). This leads to condition (∗). In the *Bottom* panel, we illustrate a similar example for *n* = 3, explaining condition (†).

The above conditions define a three-dimensional polyhedron within the space of all nice reactive-2 strategies (Fig. 2*C*). The condition $p_{\mathrm{CC}}=1$ follows from the requirement that the strategy ought to be nice. As long as the coplayer cooperates, the reactive-*n* player goes along. The other two conditions imply that for each defection in memory, the player’s cooperation rate decreases proportionally. Interestingly, in cases with a mixed 2-history (one cooperation, one defection), the above conditions suggest that the exact timing of cooperation does not matter. It is only required that the two cooperation probabilities *p*_*CD*_ and *p*_*DC*_ are sufficiently small on average. Notably, the above conditions also imply that to check whether a given reactive-2 strategy is a partner, it suffices to check two deviations. These deviations are the strategy that strictly alternates between cooperation and defection (yielding the first inequality), and ALLD (yielding the second inequality) (Fig. 3). We note that this last implication is specific to the donation game. For the general prisoner’s dilemma (depicted in Fig. 2*D*), there are more than two inequalities that need to be satisfied (*SI Appendix*).

Analogously, we can also characterize the partners among the reactive-3 strategies. A reactive-3 strategy can be represented by a vector $p=(p_{\mathrm{CCC}},p_{\mathrm{CCD}},p_{\mathrm{CDC}},p_{\mathrm{CDD}},p_{\mathrm{DCC}},p_{\mathrm{DCD}},p_{\mathrm{DDC}},$$p_{\mathrm{DDD}}).$ It is a partner strategy if and only if

[2] $$\begin{array}{c} \begin{array}{cc} p_{\mathrm{CCC}} & =1\\ \frac{p_{\mathrm{CDC}}+p_{\mathrm{DCD}}}{2} & \leq 1-\frac{1}{2}\cdot \frac{c}{b}\\ \frac{p_{\mathrm{CCD}}+p_{\mathrm{CDC}}+p_{\mathrm{DCC}}}{3} & \leq 1-\frac{1}{3}\cdot \frac{c}{b}\\ \frac{p_{\mathrm{CDD}}+p_{\mathrm{DCD}}+p_{\mathrm{DDC}}}{3} & \leq 1-\frac{2}{3}\cdot \frac{c}{b}\\ \frac{p_{\mathrm{CCD}}+p_{\mathrm{CDD}}+p_{\mathrm{DCC}}+p_{\mathrm{DDC}}}{4} & \leq 1-\frac{1}{2}\cdot \frac{c}{b}\\ p_{\mathrm{DDD}} & \leq 1-\frac{c}{b} \end{array} \end{array}$$

These conditions follow a similar logic as in the previous case with *n* = 2. For every coplayer’s defection in memory, the respective cooperation probability needs to be diminished proportionally. As an example, the second inequality in Eq. **2** considers three sequences *CCD*, *CDC*, *DCC*. The proportion of *D*’s across these three sequences is 1/3. Hence, the threshold on the right-hand side is 1−1/3·c/b. Because reactive-2 strategies are a subset of reactive-3 strategies, we can also derive the conditions in Eq. **1** as a special case of Eq. **2** (*SI Appendix*).

Moreover, the above conditions imply that to check whether a given reactive-3 strategy is a partner, it suffices to check five deviations. Similarly to the previous case, two of these deviations include the strategy that strictly alternates between cooperation and defection, and ALLD. The remaining conditions arise from deviations toward self-reactive strategies that repeat certain sequences, where the sequences are *CCD*, *CDD*, and *CCDD* (Fig. 3).

For *n* = 3, there are now more conditions to consider than in the previous case. These conditions become even more complex for the general prisoner’s dilemma. Given these complexities, we do not present conditions for reactive-*n* partner strategies beyond *n* = 3, even though the algorithm presented in the previous section still applies.

### Partners Among the Reactive-n Counting Strategies.

We can more easily generalize these formulas to arbitrary *n* if we further restrict the strategy space. In the following, we consider reactive-*n* counting strategies. These strategies only depend on how often the coplayer cooperated during the past *n* rounds; they do not take into account in which of the past *n* rounds the coplayer cooperated. We represent such strategies as a vector $r={\left(r_{i}\right)}_{i\in \{n,n-1,\cdots ,0\}}$. Each entry *r*_*i*_ indicates the player’s cooperation probability if the coplayer cooperated *i* times during the last *n* rounds. We note that although reactive-*n* counting strategies have fewer entries (bits) than reactive-*n* strategies, they are equally complex in terms of their memory requirements. Even a player with a reactive-*n* counting strategy needs to keep a record of the exact sequence of the opponent’s last *n* actions. Only by doing so, the player can update its opponent’s cooperation count each round, by discarding the opponent’s oldest action in memory (*SI Appendix*). Any reactive-1 strategy $p=(p_{C},p_{D})$ is a counting strategy by definition. However, for larger *n*, the set of counting strategies is a strict subset of the reactive-*n* strategies. For example, for *n* = 2, counting strategies are those strategies that satisfy $p_{\mathrm{CD}}=p_{\mathrm{DC}}=:r_{1}$. As a result, the partners among the counting strategies form a 2-dimensional plane within the 3-dimensional polyhedron of reactive-2 partner strategies (Fig. 2 *C* and *D*).

For the donation game, it is possible to characterize the set of partner strategies for arbitrary *n*. We find that a counting strategy **r** is a partner if and only if

[3] $$r_{n}=1\;\;\text{and}\;r_{n-k}\leq 1-\frac{k}{n}\cdot \frac{c}{b}\;\;\;\text{for}\;k\in \left\{1,2,\cdots ,n\right\}.$$

That is, for every defection of the opponent in memory, the maximum cooperation probability needs to be reduced by c/(nb). It is worth highlighting that this result is general. These strategies are Nash equilibria even if players are allowed to deviate toward strategies that do not merely count the coplayer’s cooperative acts, or toward strategies that take into account more than the last *n* rounds.

### Further Analytical Results.

In *SI Appendix*, we use our framework to derive a number of additional results. Here, we provide a brief summary. First, instead of partners we can equally use our formalism to characterize “defector strategies”—reactive-*n* strategies that lead to stable mutual defection. Following Stewart and Plotkin (63), we can use this characterization to compare the relative volume of partners and defectors. We find that for sufficiently small cost-to-benefit ratios, the set of partners has the larger volume. Moreover, the relative volume of partners increases in *n*, both for reactive-*n* and for reactive-*n* counting strategies (*SI Appendix*, Fig. S3 and Table S2). This finding has interesting implications for the evolution of cooperation (28, 63). If evolutionary processes generate mutant strategies at random, larger memory lengths make it increasingly likely that mutants adopt strategies in the vicinity of partner strategies, compared to defectors.

Second, we use our formalism to explore the effects of implementation errors (33). When such an error occurs (with some exogenous probability *ε*), players implement the opposite action of what they intend to do. For this scenario, we derive two sets of results. First, we assume errors to be vanishingly rare. In that case, we find that almost all our previously described partner strategies remain approximate Nash equilibria. That is, even if there are profitable deviations, the respective payoff advantage is guaranteed to be arbitrarily small, see *SI Appendix*, Fig. S2. Second, we derive a result for donation games when the error rate is strictly positive. In that case, we describe a subset of so-called “equalizer” strategies (67), which can sustain cooperation in equilibrium. Among the reactive-1 strategies, this set includes a single strategy, GTFT. For *n* ≥ 2, we derive additional variants of GTFT, which punish defection with some delay. While we characterize the set of reactive-*n* equalizers explicitly, it remains an open question whether they are the only partner strategies for positive error rates.

### Evolutionary Dynamics.

With our previous equilibrium analysis, we have identified the strategies that can sustain cooperation in principle. In a next step, we determine whether these strategies evolve in the first place. Here, we no longer presume that individuals would play equilibrium strategies. Rather they initially implement some random behavior. Over time, they adapt their strategies based on social learning. To model this learning process, we consider a population of individuals who update their strategies based on pairwise comparisons. The efficacy of the resulting learning process is determined by a strength of selection parameter *β*. The larger *β*, the more likely individuals imitate strategies with a higher payoff. In addition, mutations occasionally introduce new strategies. We describe the exact setup of this learning process in *Materials and Methods*. As we explain there, the process is particularly easy to explore when mutations are rare (68–71). In that case, the population is typically homogeneous, such that all players adopt the same (resident) strategy. Once a new mutant strategy appears, this strategy fixes or goes extinct before the next mutation happens. Evolutionary processes with rare mutations can be simulated more efficiently because there is an explicit formula for the mutant’s fixation probability (72).

**Fig. 4.**
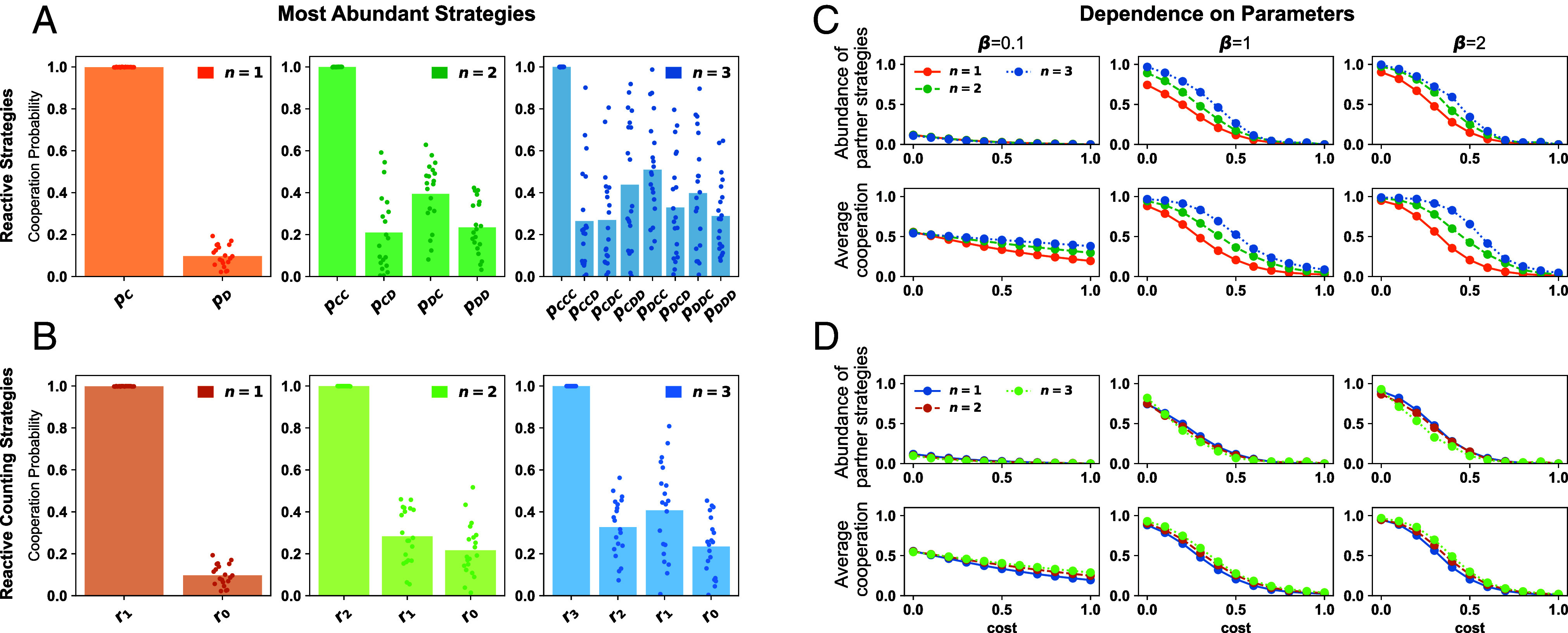
Evolutionary dynamics of reactive-*n* strategies. To explore the evolutionary dynamics among reactive-*n* strategies, we run simulations based on the method of Imhof and Nowak (68). This method assumes rare mutations. Every time a mutant strategy appears, it goes extinct or fixes before the arrival of the next mutant strategy. (*A* and *B*) We run twenty independent simulations for reactive-*n* strategies and for reactive-*n* counting strategies. For each simulation, we record the most abundant strategy (the strategy that resisted most mutants). The respective average cooperation probabilities are in line with the conditions for partner strategies. (*C* and *D*) With additional simulations, we explore the average abundance of partner strategies and the population’s average cooperation rate. For a given resident strategy to be classified as a partner by our simulation, it needs to satisfy all inequalities in the respective characterization. In addition, it needs to cooperate after full cooperation with a probability of at least 95%. For all considered parameter values, we only observe high cooperation rates when partner strategies evolve. Simulations are based on a donation game with *b* = 1, *c* = 0.5, a selection strength *β* = 1, and a population size *N* = 100, unless noted otherwise. For *n* equal to 1 and 2, simulations are run for $10^{7}$ time steps. For *n* = 3 we use $2\cdot 10^{7}$ time steps.

The results of these simulations are shown in Fig. 4. First, we explore which reactive-*n* strategies evolve for a fixed set of game parameters. Here, we vary the strategies’ memory length *n*, and whether mutations introduce all reactive-*n* strategies, or counting strategies only. For twenty independent simulations, Fig. 4 *A* and *B* displays the most abundant strategy for each simulation run (those are the strategies that prevent the largest number of mutants from taking over). We note that all the shown strategies show behavior consistent with our characterization of partners: If a coplayer fully cooperated in the previous *n* rounds, these strategies prescribe to continue with cooperation. If the coplayer defected, however, they cooperate with a markedly reduced cooperation probability that satisfies the constraints in Eqs. **1**–**3**.

Interestingly, however, the evolving strategies exhibit an interesting asymmetry. For example, for reactive-2 strategies, we observe that players’ strategies tend to satisfy $p_{\mathrm{CD}}<p_{\mathrm{DC}}$. That is, they are more likely to defect if their opponent defected last round, rather than two rounds ago. In light of our equilibrium analysis, this result is surprising. After all, according to our partner condition Eq. **1**, the two cooperation probabilities are completely interchangeable. This asymmetry arises because our evolutionary process with uniform mutations does not introduce perfect partner strategies (with $p_{\mathrm{CC}}=1$). Rather, it introduces strategies in the respective neighborhood (with, say, $p_{\mathrm{CC}}=0.99$). Among these noisy partner strategies, we show that strategies are more resilient when they punish defection without delay (for more details, see *SI Appendix*, Figs. S4–S6 and Table S3).

In a next step, we systematically explore the impact of several key parameters: the cost-to-benefit ratio c/b, the selection strength *β*, and the memory length *n* (Fig. 4 *C* and *D*). In addition, we vary the error rate *ε* in *SI Appendix*, Fig. S7. In each case, we record how these parameters affect the abundance of partner strategies and the population’s average cooperation rate. Overall, the effect of each parameter is as expected. In particular, interactions are most cooperative when cooperation is comparably cheap. This effect is magnified for stronger selection strengths. Two results, however, are particularly noteworthy. First, the curves representing evolving cooperation rates align with the prevalence of partner strategies. This observation suggests that partner strategies are indeed crucial for the evolution of cooperation. Second, the positive effects of larger memory are most pronounced for reactive-*n* strategies. In contrast, for counting strategies any positive effect of increasing *n* is considerably dampened.

We repeat these simulations for the more general sets of memory-*n* strategies and memory-*n* counting strategies (*SI Appendix*). Again, among memory-*n* strategies, larger values of *n* lead to more cooperation. But even among counting strategies, longer memory has a positive, albeit smaller, effect (*SI Appendix*, Fig. S6). We conclude for the considered strategy spaces that the timing of cooperation can be important, even in additive games such as the donation game.

## Discussion

Direct reciprocity is a key mechanism for cooperation, based on the intuition that individuals are more likely to cooperate when they meet repeatedly (8). To capture the logic of reciprocity, most previous theoretical studies focus on a subset of strategies, the memory-1 strategies (21–31). This set is comparably easy to work with: The number of deterministic memory-1 strategies is manageable; most strategies are easy to interpret; and payoffs can be computed efficiently (9). At the same time, however, this strategy space leaves out many interesting reciprocal behaviors that are of theoretical or empirical relevance. For example, already simple behaviors such as Tit-for-Two-Tat (7) are not representable with one-round memory. This shortcoming is particularly consequential for noisy games, where higher-memory strategies are important (54). In such games, individuals often take into account information from previous rounds to make sense of a coplayer’s defection in the last round. That is, the earlier history of play provides an important context to interpret the coplayer’s last-round behavior.

To make progress, we consider an easily interpretable set of strategies with higher memory. These reactive-*n* strategies take into account a coplayer’s moves during the past *n* rounds. They capture the basic idea of conditional cooperation: People are responsive to the previous actions of their interaction partners. For reactive-*n* strategies, we derive a convenient method to characterize all “partner strategies”—strategies that sustain full cooperation in a Nash equilibrium (29, 66). We show that for a reactive-*n* strategy to be a Nash equilibrium, it is not necessary to check all possible deviations. It suffices to only check deviations toward (deterministic) self-reactive-*n* strategies. Self-reactive players are particularly simple to describe. They only take into account their own previous moves. In particular, the future behavior of a self-reactive player is independent of the coplayer. We use this insight to characterize the reactive-*n* partner strategies (and the defector strategies) in the repeated prisoner’s dilemma. But the same insights should be applicable to other contexts. For example, we expect that similar techniques can be used to characterize the equilibria of other repeated games, such as the snowdrift game (73) or the volunteer’s dilemma (74). In this way, some of our technical results represent useful tools to make further progress on the theory of repeated games, similar to Press and Dyson’s insight that any memory-1 strategy has a memory-1 best response (25).

Especially for small memory lengths, the conditions for partner strategies are intuitive. For example, for the donation game with *n* = 2 rounds of memory, we end up with three conditions, see Eq. **1**. (i) If the coplayer cooperated twice, continue to cooperate; (ii) If the coplayer cooperated once, cooperate with a slightly reduced probability of 1−c/(2b) on average. (iii) If the coplayer did not cooperate at all, reduce the cooperation probability even further, to 1−c/b. As we increase the memory length to *n* ≥ 3, or as we consider more general games, there are more conditions to satisfy, and the conditions become harder to interpret. However, the three simple conditions do generalize to larger *n* if we focus on the set of counting strategies. These are the reactive-*n* strategies that react to how often the coplayer cooperated during the last *n* rounds. For counting strategies, we show that for each defection of the coplayer in memory, a partner reduces its cooperation probability by c/(nb). A partner’s generosity decreases in proportion to their opponent’s selfishness.

While in practice, people’s cooperative decisions often depend on the outcome of their last encounter, they rarely depend on that last encounter only. Overall, our results suggest a way how individuals can integrate information from previous interactions to cooperate most effectively.

## Materials and Methods

Our study combines two independent approaches, an equilibrium analysis and evolutionary simulations.

### Equilibrium Analysis.

Here, we only summarize our approach; all details are in *SI Appendix*. There, we formally introduce the three relevant strategy spaces, memory-*n* strategies, reactive-*n* strategies, and self-reactive-*n* strategies. Then we provide an explicit algorithm for computing these strategies’ payoffs. This algorithm uses a Markov chain approach. The states of the Markov chain are the possible combinations of *n*-histories of the two players. Given the players’ current *n*-histories and their strategies, we can compute the likelihood of observing each possible state one round later.

In a second step, we explore the partner strategies among the reactive-*n* strategies. To this end, we first generalize some well-known reactive-1 partner strategies: Tit-for-Tat (7) and Generous Tit-for-Tat (64, 65). In a next step, we derive a general algorithm to check whether a given reactive-*n* strategy is a partner. We use this algorithm to characterize all reactive-*n* partners for n∈{1,2,3}, for both the donation game and the prisoner’s dilemma. For counting strategies in the donation game, we characterize partners for all *n*.

### Evolutionary Analysis.

For our simulations, we consider a population of size *N*. Initially all members are of the same strategy (in our case, they are unconditional defectors). In each elementary time step, one individual switches to a new mutant strategy. The mutant strategy is generated by independently drawing each individual cooperation probability from the unit interval [0,1] uniformly at random. If the mutant strategy yields a payoff of $\pi_{M,k}$, where *k* is the number of mutants in the population, and if residents get a payoff of $\pi_{R,k}$, then the fixation probability *ϕ*_*M*_ of the mutant strategy can be calculated explicitly (72),

[4] $$\phi_{M}=(1+\sum_{i=1}^{N-1}\prod_{j=1}^{i}e^{-\beta (\pi_{M,j}-\pi_{R,j})}{)}^{-1}.$$

The parameter *β* ≥ 0 reflects the strength of selection. It measures the importance of relative payoff advantages for the evolutionary success of a strategy. When *β* is small, *β* ≈ 0, payoffs become irrelevant, and a strategy’s fixation probability approaches $\phi_{M}\approx 1/N$. The larger the value of *β*, the more strongly the evolutionary process favors the fixation of strategies with a high payoff. Depending on *ϕ*_*M*_, the mutant either fixes (becomes the new resident) or goes extinct. Afterward, the process repeats, and another mutant strategy is introduced to the population.

We iterate this elementary population updating process for a large number of mutant strategies. At each step, we record the current resident strategy and the resulting average cooperation rate, indicating how often the resident strategy cooperates with itself. Additionally, we assess how many resident strategies qualify as partner strategies in our simulation. For a resident strategy to be classified as a partner, it must satisfy all inequalities in the respective definition of partner strategies. In addition, it must cooperate with a probability of at least 95% after full cooperation.

## Supplementary Material

Appendix 01 (PDF)

## Data Availability

The source code used to reproduce the results of this study is available on the online GitHub repository: Nikoleta-v3/conditional-cooperation-with-longer-memory (75). The simulation data have been archived on Zenodo and can be found at zenodo.org/records/10605988 (76).
