# Supplementary material for: Conditional cooperation with longer memory
Source: Proc Natl Acad Sci U S A. 2024 Dec 6;121(50):e2420125121. doi: 10.1073/pnas.2420125121 (PMC11648855; doi:10.1073/pnas.2420125121)
Supplement: Supplementary file 1 — Appendix 01 (PDF) [file pnas.2420125121.sapp.pdf]

Supporting Information for

Conditional cooperation with longer memory

Nikoleta E. Glynatsi, Ethan Akin, Martin A. Nowak, Christian Hilbe

Nikoleta E. Glynatsi.
E-mail: glynatsi@evolbio.mpg.de

This PDF file includes:

Supporting text
Figs. S1 to S8
Tables S1 to S3
SI References

Contents

1 An overview of the related literature **3**

2 Model and basic results **5**
- A The repeated prisoner's dilemma . . . 5
- B Finite-memory strategies . . . 5
- C Computing the payoffs of finite-memory strategies . . . 6
- D An Extension of Akin's Lemma . . . 7

3 Characterizing the partner strategies among the reactive-n strategies **8**
- A Partner strategies . . . 8
- B Zero-determinant strategies with n rounds memory . . . 8
- C An algorithm to check whether a reactive-n strategy is a Nash equilibrium . . . 9
- D Reactive partner strategies in the donation game . . . 11
- E Reactive partner strategies in the general prisoner's dilemma . . . 13
- F Defector strategies in the donation game . . . 13
- G Games with errors . . . 14

4 Further numerical results **18**
- A Volume of partner and defector strategies . . . 18
- B Exploring an emerging asymmetry in the evolving strategies . . . 18
- C Evolutionary dynamics among memory-n strategies . . . 21
- D Evolution of cooperation in games with errors . . . 22

5 Appendix: Proofs **25**
- A Proof of Lemma 1: Akin's lemma . . . 25
- B Proof of Lemma 2: Sufficiency of testing self-reactive strategies . . . 25
- C Proof of Theorem 1: Sufficiency of pure self-reactive strategies . . . 26
- D Proof of Theorem 2: Reactive-2 partner strategies in the donation game . . . 27
- E Proof of Theorem 3: Reactive-3 partner strategies in the donation game . . . 28
- F Proof of Theorem 4: Reactive-n counting strategies in the donation game . . . 28
- G Proof of Theorem 5: Reactive-2 partner strategies in the prisoner's dilemma . . . 30
- H Proof of Theorem 6: Reactive-3 partner strategies in the prisoner's dilemma . . . 31
- I Proof of Theorem 7: Reactive-n defector strategies in the donation game . . . 31

6 Supplementary References **33**

Supporting Information Text

This document provides further details on the previous literature, our methodology, our analytical findings, and our evolutionary simulations. Section 1 describes previous work on direct reciprocity with longer memory. For several important articles, we summarize which key question they explore, and how our article further contributes to this strand of work. Section 2 summarizes our model. Here, we introduce all relevant strategy spaces, and we show how to compute long-term payoffs for strategies with more than one-round memory. Section 3 contains our key analytical results. Here, we define partner strategies, we present an algorithm that allows us to verify whether a given reactive-n strategy is a partner, and we apply this algorithm to fully characterize the reactive-n partner strategies for $n=2$ and $n=3$. We perform a similar analysis for the stable defecting strategies. Section 4 presents some complementary evolutionary results. Finally, the Appendix in Section 5 contains the proofs of our mathematical results.

1. An overview of the related literature

By now, there is a vast literature on the evolution of reciprocal cooperation in repeated games. This literature has highlighted various strategies that either succeed in evolutionary simulations and computer tournaments, or that exhibit some interesting theoretical properties. This literature also describes how results depend on the payoffs of the game, or on implementation errors. For a general overview on these results, we refer to the reviews and text books in Refs. (1–5). In the following we discuss some key papers that discuss the role of memory on direct reciprocity.

Particular higher-memory strategies. There are several papers that highlight particular strategies with more than one-round memory. For example, Yi *et al* (6) describes a variant of Tit-for-Tat (`TFT`), referred to as `TFT-ATFT`. This strategy depends on the outcome of the last two rounds. It typically repeats the co-player's previous move, just as `TFT` does. But in case the focal player defected because of an error, the strategy prescribes to do the opposite of the co-player's previous move. The authors show that `TFT-ATFT` is robust with respect to implementation errors, and that it shows a good performance in evolutionary simulations.

Following up on this work, Baek and Murase (7) introduce a class of strategies called 'friendly rivals'. These strategies mutually cooperate with themselves. At the same time, a player with such a strategy always gets at least the payoff of its opponent, irrespective of the opponent's strategy. In particular, such strategies satisfy our notion of being a partner. While explicitly considering all pure strategies with three rounds of memory, they highlight one strategy that performs particularly well, referred to as `CAPRI`. In (8), the authors examine whether friendly rivals emerge in an evolutionary setting. They conclude that in well-mixed populations, friendly rivals play a minor role. Instead, partner strategies often seem to be sufficient to promote cooperation.

Hilbe et al (9) use an axiomatic approach to derive stable memory-n strategies of direct reciprocity. They impose three conditions on the strategies they look for: (i) *Mutual cooperation:* A strategy ought to fully cooperate with itself. (ii) *Retaliation:* Once a co-player defects, the strategy ought to defect for at least k rounds. (iii) *Error-correction:* When two players with that strategy interact, they return to mutual cooperation after at most k rounds, after any possible previous history. The authors find that there is a class of memory-n strategies that satisfies these properties. They refer to the elements of this class as All-or-None strategies, or $\texttt{AON}_k$. These $\texttt{AON}_k$ strategies satisfy the conditions for partner strategies. The well-known strategy Win-Stay Lose-Shift (10) is a special case of an $\texttt{AON}_k$-strategy with $k = 1$.

Finally, Li *et al* (11) describe a strategy of *cumulative reciprocity*, or `CURE`. This strategy counts how often the two players cooperated in all previous rounds. A player with that strategy cooperates if the co-player's cooperation count is above a certain threshold (that depends on the player's own cooperation count). `CURE` satisfies the properties of a partner strategy. Moreover, the authors show that the strategy performs well when competing with memory-1 strategies.

Compared to these previous articles, we take a more systematic approach. Instead of describing particular partner strategies, we aim to identify *all* partner strategies within an important strategy space, the space of (stochastic) reactive-n strategies. For the special case of reactive-n counting strategies, we provide explicit conditions for all memory lengths n. For the more general case of reactive-n strategies, we provide explicit conditions for $n = 2$ and $n = 3$. Moreover, we provide a general algorithm to test whether a given reactive-n strategy is a partner. This algorithm is valid for any n.

Particular classes of higher-memory strategies. In addition to the work on particular higher-memory strategies, there are also several articles that describe properties of certain strategy classes. One such class is the set of *zero-determinant* (ZD) strategies. This strategy class was first described by Press and Dyson (12). They showed that there are certain memory-1 strategies that can unilaterally enforce a linear relationship between the players' payoffs. Ueda (13, 14) extends this formalism to memory-n strategies. To this end, he derives a version of what we refer to as Akin's lemma (see Section 3). Based on this result, he describes different variants of Tit-for-Tat.

Another strategy class that has been studied in more detail is the class of *reactive learning strategies*, as introduced by McAvoy and Nowak (15). These strategies gradually modify their cooperation propensity each round, based on the opponent's last action. As a result, one player's action can have a lasting effect on the co-player's behavior. McAvoy and Nowak focus on the space of feasible payoffs (i.e., the payoffs that can be achieved when one player's strategy is fixed). They define a map that assigns to any memory-one strategy an associated reactive learning strategy. The feasible payoffs against this reactive learning strategy are a strict subset of the feasible payoffs against the original memory-one strategy. McAvoy and Nowak interpret this result as an indication that players with reactive-learning strategies have more control over the resulting payoffs.

While both the papers by Ueda (13, 14) and by McAvoy and Nowak (15) offer some remarkable mathematical results, their scope is different from ours. They aim to derive general properties of certain strategy classes, independent of whether the resulting strategies form a Nash equilibrium. In particular, they do not explore which of their strategies can sustain cooperation (i.e., which of their strategies satisfies the notion of being a partner). Partner strategies are the main focus of our work.

Work on evolutionary robust cooperating strategies. Perhaps the paper most closely related to ours is the work by Stewart and Plotkin (16). They consider memory-n strategies for public goods games in groups of size $m \geq 2$. They particularly focus on those strategies that are 'evolutionary robust' – strategies that resist selective invasion by any mutant. In the limit of large populations, evolutionary robust strategies are closely related to the Nash equilibria of the repeated game (17). Using an elegant coordinate transform, Stewart and Plotkin (16) give a rigorous characterization of those evolutionary robust strategies that sustain cooperation. They use this characterization to compute the respective volume of evolutionary robust cooperating strategies. Analytically and with simulations, they show that this volume increases in the player's memory, relative to the

respective volume of evolutionary robust strategies that lead to mutual defection. Finally, using simulations, they show that when the players' strategies co-evolve with their memory capacity, then evolution favors larger memory capacities.

Stewart and Plotkin's robustness results are related to ours. Importantly, however, even in the case of $m=2$ players, their characterization of evolutionary robust strategies is implicit. For example, for cooperating strategies, their Eq. (2) contains terms w^{l_0,l_p}. These terms in turn depend on the specific mutant strategy considered. Especially for larger values of n, these terms become increasingly expensive to compute. Moreover, in principle, one would have to check these conditions for all possible mutants. The authors argue that only four particular cases of w^{l_0,l_p} need to be studied (as described in their SI Section 5.4). However, two of these cases correspond to an entire family of possible mutant strategies. In one case, one needs to check mutants that play such that the two players do not defect for n consecutive rounds. In the other case, one needs to check mutants that make sure the two players do not cooperate for n consecutive rounds. Because there are many such mutants, these two cases can lead to more than two inequalities when characterizing the set of evolutionary robust strategies. In fact, for our example of finding the partners among the reactive-n counting strategies, we find that these two cases give rise to $n-1$ inequalities. Together with the condition that the strategy ought to be robust against `ALLD`, we obtain n conditions overall.

In contrast to Stewart and Plotkin's work, we give fully explicit conditions for the partner strategies among the reactive-2, reactive-3, and for reactive-n counting strategies. To this end, we make important conceptual progress. We show that to test whether any given reactive-n strategy is a Nash equilibrium, it suffices to compare it to all pure self-reactive-n strategies. Similar results could in future help to prove further results on the stability of other classes of memory-n strategies. Finally, we perform extensive simulations to study the evolution of reactive-n and reactive-n counting strategies, for $n \in \{1, 2, 3\}$.

2. Model and basic results

A. The repeated prisoner's dilemma. We consider the infinitely repeated prisoner's dilemma between two players, player 1 and player 2. Each round, each player can either cooperate (C) or defect (D). The resulting payoffs are given by the matrix

$$\begin{array}{c} \\ C \\ D \end{array} \begin{array}{c} \begin{array}{cc} C & D \end{array} \\ \left(\begin{array}{cc} R & S \\ T & P \end{array}\right) \end{array}. \qquad [1]$$

Here, R is the reward payoff of mutual cooperation, T is the temptation to defect, S is the sucker's payoff, and P is the punishment payoff for mutual defection. For the game to be a prisoner's dilemma, we require

$$T > R > P > S \quad \text{and} \quad 2R > T+S. \qquad [2]$$

That is, mutual cooperation is the best outcome to maximize the players' total payoffs, but each player's dominant action is to defect. For some of our results, we focus on a special case of the prisoner's dilemma, the donation game. This game only depends on two parameters, the benefit b and the cost c of cooperation. The payoff matrix of the donation game takes the form

$$\begin{array}{c} \\ C \\ D \end{array} \begin{array}{c} \begin{array}{cc} C & D \end{array} \\ \left(\begin{array}{cc} b-c & -c \\ b & 0 \end{array}\right) \end{array}. \qquad [3]$$

For this game to satisfy the conditions Eq. (2) of a prisoner's dilemma, we assume $b>c>0$ throughout.

Players interact in the repeated prisoner's dilemma for infinitely many rounds, and future payoffs are not discounted. A strategy σ^i for player i is a rule that tells the player what to do in any given round, depending on the outcome of all previous rounds. Given the player's strategies σ^1 and σ^2, one can compute each player i's expected payoff $\pi^i_{\sigma^1,\sigma^2}(t)$ in round t. For the repeated game, we define the players' payoffs as the expected payoff per round,

$$\pi^i(\sigma^1,\sigma^2) = \lim_{\tau\to\infty} \frac{1}{\tau} \sum_{t=1}^{\tau} \pi^i_{\sigma^1,\sigma^2}(t). \qquad [4]$$

For general strategies σ^1 and σ^2, the above limit may not always exist. Problems may arise, for example, if one of the players cooperates in the first round, defects in the two subsequent rounds, cooperates in the four rounds thereafter, etc., which prevents the time averages from converging. However, in the following, we focus on strategies with finite memory. When both players adopt such a strategy, the existence of the limit in Eq. (4) is guaranteed, as we discuss further below.

B. Finite-memory strategies. In this study, we focus on strategies that ignore all events that happened more than n rounds ago. To define these strategies, we need some notation. An *n-history for player i* is a string $\mathbf{h^i} = (a^i_{-n}, \dots, a^i_{-1}) \in \{C,D\}^n$. We interpret the string's entry a^i_{-k} as player i's action k rounds ago. We denote the space of all n-histories for player i as H^i. This space contains $|H^i| = 2^n$ elements. A pair $\mathbf{h} = (\mathbf{h^1}, \mathbf{h^2})$ is called an *n-history of the game*. We use $H = H^1 \times H^2$ to denote the space of all such histories, which contains $|H| = 2^{2n}$ elements.

Memory-n strategies. Based on this notation, a *memory-n strategy* for player i as a tuple $\mathbf{m} = (m_\mathbf{h})_{\mathbf{h}\in H}$. Each input $\mathbf{h} = (h^i, h^{-i})$ refers to a possible n-history, where now $\mathbf{h^i}$ and $\mathbf{h^{-i}}$ refer to the n-histories of the focal player and the co-player, respectively. The corresponding output $m_\mathbf{h} \in [0,1]$ is the focal player's cooperation probability in the next round, contingent on the outcome of the previous n rounds. We refer to the set of all memory-n strategies as

$$\mathcal{M}_n := \left\{ \mathbf{m} = (m_\mathbf{h})_{\mathbf{h}\in H} \;\middle|\; 0 \le m_\mathbf{h} \le 1 \text{ for all } \mathbf{h}\in H \right\} = [0,1]^{2^{2n}}. \qquad [5]$$

This definition leaves the strategy's actions during the first n rounds unspecified, for which no complete n-history is yet available. However, because we consider infinitely repeated games without discounting, these first n rounds are usually irrelevant, as we show further below. In the following, we therefore only specify a strategy's move during the first n rounds when necessary.

Among all memory-n spaces $\mathcal{M}_1$, the one with $n=1$ is the most frequently studied. Memory-1 strategies take the form $\mathbf{m} = (m_{CC}, m_{CD}, m_{DC}, m_{DD})$. The first index refers to the focal player's last action (1-history) and the second index refers to the co-player's last action. One well-known example is Win-Stay Lose-Shift (10), $\mathbf{m} = (1,0,0,1)$, but there are many others (4).

Reactive-n strategies. For our following analysis, two particular subsets of memory-n strategies will play an important role. The first subset is the set of *reactive-n strategies*,

$$\mathcal{R}_n := \left\{ \mathbf{m} \in \mathcal{M}_n \;\middle|\; m_{(\mathbf{h^i},\mathbf{h^{-i}})} = m_{(\tilde{\mathbf{h}}^\mathbf{i},\mathbf{h^{-i}})} \text{ for all } \mathbf{h^i}, \tilde{\mathbf{h}}^\mathbf{i} \in H^i \text{ and } \mathbf{h^{-i}} \in H^{-i} \right\}. \qquad [6]$$

Reactive-n strategies are independent of the focal player's own n-history. The space of reactive-n strategies can be naturally identified with the space of all 2^n-dimensional vectors

$$\mathbf{p} = (p_{\mathbf{h^{-i}}})_{\mathbf{h^{-i}}\in H^{-i}} \quad \text{with} \quad 0 \le p_{\mathbf{h^{-i}}} \le 1 \quad \text{for all} \quad \mathbf{h^{-i}} \in H^{-i}. \qquad [7]$$

In this reduced representation, each entry $p_{\mathbf{h^{-i}}}$ corresponds to the player's cooperation probability in the next round based on the co-player's actions in the previous n rounds. Again, the most studied case of reactive-n strategies is when $n=1$. Here, the reduced representation according to Eq. (7) takes the form $\mathbf{p}=(p_C, p_D)$. Probably the best-known example of a reactive-1 strategy is Tit-for-Tat, `TFT` (18). `TFT` cooperates if and only if the co-player cooperated in the previous round. Hence, its memory-1 representation is $\mathbf{m}=(1,0,1,0)$, whereas its reduced representation is $\mathbf{p}=(1,0)$. Another example is the strategy Generous Tit-for-Tat, `GTFT` (19, 20). `GTFT` occasionally cooperates even if the co-player defected. For that strategy, the memory-1 representation is $\mathbf{m}=(1,p_D^*,1,p_D^*)$, and the reduced representation is $\mathbf{p}=(1,p_D^*)$, where

$$p_D^* := \min\left\{1-(T-R)/(R-S),\ (R-P)/(T-P)\right\}. \qquad [8]$$

In the special case that payoffs are given by the donation game, this condition simplifies to $p_D^*=1-c/b$.

Self-reactive-n strategies.. The other important subspace of memory-n strategies is the set of self-reactive-n strategies,

$$\mathcal{S}_n := \left\{\mathbf{m}\in\mathcal{M}_n \;\middle|\; m_{(\mathbf{h^i},\mathbf{h^{-i}})} = m_{(\mathbf{h^i},\tilde{\mathbf{h}}^{-\mathbf{i}})} \text{ for all } \mathbf{h^i}\in H^i \text{ and } \mathbf{h^{-i}},\tilde{\mathbf{h}}^{-\mathbf{i}}\in H^{-i}\right\}. \qquad [9]$$

These strategies only depend on the focal player's own decisions during the last n rounds, independent of the co-player's decisions. Again, we can identify any self-reactive-n strategies with a 2^n-dimensional vector,

$$\tilde{\mathbf{p}} = (\tilde{p}_{\mathbf{h^i}})_{\mathbf{h^i}\in H^i} \quad \text{with} \quad 0\le\tilde{p}_{\mathbf{h^i}}\le 1 \quad \text{for all} \quad \mathbf{h^i}\in H^i. \qquad [10]$$

Each entry $\tilde{p}_{\mathbf{h^i}}$ corresponds to the player's cooperation probability in the next round, contingent on the player's own actions in the previous n rounds. A special subset of self-reactive strategies is given by the round-k-repeat strategies, for some $1\le k\le n$. In any given round, players with a *round-k-repeat strategy* $\tilde{\mathbf{p}}^{k-\mathrm{Rep}}$ choose the same action as they did k rounds ago. Formally, the entries of $\tilde{\mathbf{p}}^{k-\mathrm{Rep}}$ are defined by

$$\tilde{p}^{k-\mathrm{Rep}}_{\mathbf{h^i}} = \begin{cases} 1 & \text{if } a^i_{-k}=C \\ 0 & \text{if } a^i_{-k}=D. \end{cases} \qquad [11]$$

From this point forward, we will use the notations $\mathbf{m}$, $\mathbf{p}$, and $\tilde{\mathbf{p}}$ to denote memory-n, reactive-n, and self-reactive-n strategies, respectively. We say these strategies are *pure* or *deterministic* if all conditional cooperation probabilities are either zero or one. If all cooperation probabilities are strictly between zero and one, we say the strategy is *strictly stochastic*. When it is convenient to represent the self-reactive repeat strategies as elements of the memory-n strategy space, we write $\mathbf{m}^{k-\mathrm{Rep}}\in[0,1]^{2^{2n}}$ instead of $\tilde{\mathbf{p}}^{k-\mathrm{Rep}}\in[0,1]^{2^n}$.

C. Computing the payoffs of finite-memory strategies.

A Markov chain representation. A repeated game between two players with memory-n strategies $\mathbf{m^1}$ and $\mathbf{m^2}$ can be represented as a Markov chain. The states of the Markov chain are the possible n-histories $\mathbf{h}\in H$. To compute the transition probabilities from one state to another within one round, suppose players currently have the n-history $\mathbf{h}=(\mathbf{h^1},\mathbf{h^2})$ in memory. Then the transition probability that the state after one round is $\tilde{\mathbf{h}}=(\tilde{\mathbf{h}}^{\mathbf{1}},\tilde{\mathbf{h}}^{\mathbf{2}})$ is a product of two factors,

$$M_{\mathbf{h},\tilde{\mathbf{h}}} = x^1\cdot x^2, \qquad [12]$$

The two factors represent the (independent) decisions of the two players,

$$x^i = \begin{cases} m^i_{(\mathbf{h^i},\mathbf{h^{-i}})} & \text{if } \tilde{a}^i_{-1}=C, \ \text{ and } \ \tilde{a}^i_{-t}=a^i_{-t+1} \ \text{ for } \ t\in\{2,\dots,n\} \\ 1-m^i_{(\mathbf{h^i},\mathbf{h^{-i}})} & \text{if } \tilde{a}^i_{-1}=D, \ \text{ and } \ \tilde{a}^i_{-t}=a^i_{-t+1} \ \text{ for } \ t\in\{2,\dots,n\} \\ 0 & \text{if } \tilde{a}^i_{-t}\neq a^i_{-t+1} \ \text{ for some } \ t\in\{2,\dots,n\}. \end{cases} \qquad [13]$$

The resulting $2^{2n}\times 2^{2n}$ transition matrix $M=(M_{\mathbf{h},\tilde{\mathbf{h}}})$ fully describes the game dynamics after the first n rounds. More specifically, suppose $\mathbf{v}(t)=\big(v_{\mathbf{h}}(t)\big)_{\mathbf{h}\in H}$ is the probability distribution of observing state $\mathbf{h}$ after players made their decisions for round $t\ge n$. Then the respective probability distribution after round $t+1$ is given by $\mathbf{v}(t+1)=\mathbf{v}(t)\cdot M$. The long-run dynamics is particularly simple to describe when the matrix M is primitive (which happens, for example, when the two strategies $m^i_{\mathbf{h}}$ are strictly stochastic). In that case, it follows by the theorem of Perron and Frobenius that $\mathbf{v}(t)$ converges to some $\mathbf{v}$ as $t\to\infty$. As a result, also the respective time average exists and converges to $\mathbf{v}$,

$$\mathbf{v} = \lim_{\tau\to\infty}\frac{1}{\tau}\sum_{t=n}^{n+\tau-1}\mathbf{v}(t). \qquad [14]$$

This limiting distribution $\mathbf{v}$ can be computed as the unique solution of the system $\mathbf{v}=\mathbf{v}M$, with the additional constraint that the entries of $\mathbf{v}$ need to sum up to one.

But even when M is not ergodic, $\mathbf{v}(t)$ still converges to an invariant distribution $\mathbf{v}$ that satisfies $\mathbf{v}=\mathbf{v}M$. However, in that case, the system $\mathbf{v}=\mathbf{v}M$ no longer has a unique solution. Instead, the limiting distribution $\mathbf{v}$ depends on the very first n-history after the first n rounds, $\mathbf{v}(n)$, which in turn depends on the players' moves during the first n rounds.

A formula for the payoffs among memory-n players. Based on the above considerations, we can derive an explicit formula for the payoffs according to Eq. (4) when players use memory-n strategies $\mathbf{m^1}$ and $\mathbf{m^2}$. To this end, we introduce a 2^{2n}-dimensional vector $\mathbf{g^i}(k) = \big(g^i_{\mathbf{h}}(k)\big)_{\mathbf{h}\in H}$, that takes an n-history $\mathbf{h}$ as an input and returns player i's payoff k rounds ago, for $k \leq n$. That is,

$$g^i_{\mathbf{h}}(k) = \begin{cases} R & \text{if } a^i_{-k} = C \text{ and } a^{-i}_{-k} = C \\ S & \text{if } a^i_{-k} = C \text{ and } a^{-i}_{-k} = D \\ T & \text{if } a^i_{-k} = D \text{ and } a^{-i}_{-k} = C \\ P & \text{if } a^i_{-k} = D \text{ and } a^{-i}_{-k} = D. \end{cases} \qquad [15]$$

Now for a given $t \geq n$, given that $\mathbf{v}(t)$ captures the state of the system after round t, we can write player i's expected payoff in that round as

$$\pi^i_{\mathbf{m^1},\mathbf{m^2}}(t) = \big\langle\, \mathbf{v}(t),\, \mathbf{g^i}(1) \,\big\rangle = \sum_{\mathbf{h}\in H} v_{\mathbf{h}}(t) \cdot g^i_{\mathbf{h}}(1). \qquad [16]$$

As a result, we obtain for the player's average payoff across all rounds

$$\begin{aligned} \pi^i(\mathbf{m^1},\mathbf{m^2}) &\overset{\text{Eq. (4)}}{=} \lim_{\tau\to\infty} \frac{1}{\tau} \sum_{t=1}^{\tau} \pi^i_{\mathbf{m^1},\mathbf{m^2}}(t) \;=\; \lim_{\tau\to\infty} \frac{1}{\tau} \sum_{t=n}^{n+\tau-1} \pi^i_{\mathbf{m^1},\mathbf{m^2}}(t) \\ &\overset{\text{Eq. (16)}}{=} \lim_{\tau\to\infty} \frac{1}{\tau} \sum_{t=n}^{n+\tau-1} \big\langle\, \mathbf{v}(t),\, \mathbf{g^i}(1) \,\big\rangle \;=\; \Big\langle \lim_{\tau\to\infty} \frac{1}{\tau} \sum_{t=n}^{n+\tau-1} \mathbf{v}(t)\,,\, \mathbf{g^i}(1) \Big\rangle \\ &\overset{\text{Eq. (14)}}{=} \big\langle\, \mathbf{v}, \mathbf{g^i}(1) \,\big\rangle. \end{aligned} \qquad [17]$$

That is, given we know the invariant distribution $\mathbf{v}$ that captures the game's long-run dynamics, it is straightforward to compute payoffs by taking the scalar product with the vector $\mathbf{g^i}(1)$. With a similar approach as in Eq. (17), one can also show

$$\big\langle\, \mathbf{v}, \mathbf{g^i}(1) \,\big\rangle \;=\; \big\langle\, \mathbf{v}, \mathbf{g^i}(2) \,\big\rangle \;=\; \ldots \;=\; \big\langle\, \mathbf{v}, \mathbf{g^i}(n) \,\big\rangle. \qquad [18]$$

That is, to compute player i's expected payoff, it does not matter whether one refers to the last round of an n-history or to an earlier round of an n-history. All rounds k with $1 \leq k \leq n$ are equivalent.

D. An Extension of Akin's Lemma. The above Markov chain approach allows us to analyze games when both players adopt memory-n strategies. But even if only one player adopts a memory-n strategy (and the other player's strategy is arbitrary), one can still derive certain constraints on the game's long-run dynamics. One such constraint was first described by Akin (21): if player 1 adopts a memory-1 strategy $\mathbf{m}$ against an arbitrary opponent, and if the time average $\mathbf{v}$ defined by the right hand side of Eq. (14) exists, then

$$\big\langle\, \mathbf{v}\,, \mathbf{m} - \mathbf{m}^{1-\text{Rep}} \,\big\rangle = 0. \qquad [19]$$

That is, the limiting distribution $\mathbf{v}$ needs to be orthogonal to the vector $\mathbf{m} - \mathbf{m}^{1-\text{Rep}}$. This result has been termed *Akin's Lemma* (22). With similar methods as in Ref. (21), one can generalize this result to the context of memory-n strategies (13). Here, we provide the version that will be most relevant for us.

Lemma 1 (A generalized version of Akin's Lemma)
Suppose player 1 uses a memory-n strategy and player 2 uses an arbitrary strategy. For the resulting game and $t \geq n$, let $\mathbf{v}(t) = \big(v_{\mathbf{h}}(t)\big)_{\mathbf{h}\in H}$ denote the probability distribution of observing each possible n-history $\mathbf{h} \in H$ after players made their decisions for round t. Moreover, suppose the respective time average $\mathbf{v}$ according to Eq. (14) *exists. Then for each k with $1 \leq k \leq n$, we obtain*

$$\big\langle\, \mathbf{v}\,, \mathbf{m} - \mathbf{m}^{k-Rep} \,\big\rangle = 0. \qquad [20]$$

All proofs are presented in the Appendix. Here we provide an intuition. The expression $\langle \mathbf{v}, \mathbf{m} \rangle = \sum_{\mathbf{h}} v_{\mathbf{h}} m_{\mathbf{h}}$ can be interpreted as player 1's average cooperation rate across all rounds of the repeated game. To compute that average cooperation rate, one first draws an n-history $\mathbf{h}$ (with probability $v_{\mathbf{h}}$), and then one computes how likely player 1 would cooperate in the subsequent round (with probability $m_{\mathbf{h}}$). Alternatively, one could compute the average cooperation rate by drawing an n-history $\mathbf{h}$ and then checking how likely player 1 was to cooperate k rounds ago, according to that n-history. That second interpretation leads to the expression $\langle \mathbf{v}, \mathbf{m}^{k-\text{Rep}} \rangle$. According to Eq. (20), both interpretations are equivalent.

3. Characterizing the partner strategies among the reactive-n strategies

A. Partner strategies. In this study, we are interested in identifying strategies that can sustain full cooperation in a Nash equilibrium. Strategies with these properties have been termed as being of *Nash type* by Akin (21), or as *partner strategies* by Hilbe *et al* (23). In the following, we formally define them.

Definition (Partner strategies)

(i) A strategy σ for the repeated prisoner's dilemma is a *Nash equilibrium* if it is a best response to itself. That is, we require $\pi^1(\sigma,\sigma)$ to exist and

$$\pi^1(\sigma,\sigma) \geq \pi^1(\sigma',\sigma) \quad \text{for all other strategies } \sigma' \text{ for which } \pi^1(\sigma',\sigma) \text{ exists.} \qquad [21]$$

(ii) A player's strategy is *nice*, if the player is never the first to defect.

(iii) A *partner strategy* is a strategy that is both nice and a Nash equilibrium.

Several remarks are in order. First, we note that when two players with nice strategies interact, they both cooperate in every round. Partner strategies thus sustain mutual cooperation in a Nash equilibrium. Second, if a memory-n strategy $\mathbf{m}=(m_{\mathbf{h}})_{\mathbf{h}\in H}$ is to be nice, it needs to cooperate after n rounds of mutual cooperation. In other words, if $\mathbf{h_C}=(\mathbf{h^i_C},\mathbf{h^{-i}_C})$ is the n-history that consists of mutual cooperation for the past n rounds, then the strategy needs to respond by cooperating with certainty, $m_{\mathbf{h_C}}=1$. Similarly, a nice reactive-n strategy needs to satisfy $p_{\mathbf{h^{-i}_C}}=1$. Third, we note that our definition of Nash equilibria only requires that players cannot profitably deviate towards strategies *for which a payoff can be defined*. If the strategy σ is a memory-n strategy, in the following we make the slightly looser requirement that the strategy is a best response among all σ' for which the limit Eq. (14) exists. Fourth, in general it is a difficult task to verify that any given strategy σ is a Nash equilibrium. After all, one needs to verify that it yields the highest payoff according to Eq. (21) among all (uncountably) many alternative strategies σ'. Fortunately, the situation is somewhat simpler if the strategy under consideration is a memory-n strategy. In that case, it follows from an argument by Press and Dyson (12) that one only needs to compare the strategy to all other memory-n strategies. However, this still leaves us with uncountably many strategies to check. In fact, it is one aim of this paper to show that for reactive-n strategies, it suffices to check finitely many alternative strategies.

B. Zero-determinant strategies with n rounds memory. Before we provide a general algorithm to identify reactive-n partner strategies, we first generalize some of the well-known reactive-1 partner strategies, `TFT` and `GTFT`, to the case of memory-n. To this end, we use Lemma 1 to develop a theory of zero-determinant strategies within the class of memory-n strategies, see also Refs. (13, 14). In the following, we say a memory-n strategy $\mathbf{m}$ is a *zero-determinant strategy* if there is a number γ and vectors $\boldsymbol{\alpha}=(\alpha_1,\ldots,\alpha_n)$, $\boldsymbol{\beta}=(\beta_1,\ldots,\beta_n)$, $\boldsymbol{\delta}=(\delta_1,\ldots,\delta_n)$ with $\sum_{k=1}^n \delta_k = 1$ such that $\mathbf{m^i}$ can be written as

$$\mathbf{m^i} = \sum_{k=1}^n \alpha_k\, \mathbf{g}^i(k) + \sum_{k=1}^n \beta_k\, \mathbf{g}^{-i}(k) + \gamma\,\mathbf{1} + \sum_{k=1}^n \delta_k \mathbf{m}^{k-\text{Rep}}. \qquad [22]$$

In this expression, $\mathbf{g}^i(k)$ is the vector that returns player i's payoff k rounds ago, as defined by Eq. (15), $\mathbf{m}^{k-\text{Rep}}$ is the memory-n strategy that repeats player i's own move k rounds ago, and $\mathbf{1}$ is the 2^{2n}-dimensional vector for which every entry is one. If we define $\hat{\alpha}=\sum_{k=1}^n \alpha_i$ and $\hat{\beta}=\sum_{k=1}^n \beta_i$, we obtain based on Akin's lemma

$$\begin{aligned}
0 &\overset{\text{Eq. (20)}}{=} \sum_{k=1}^n \delta_k \big\langle \mathbf{v}, \mathbf{m^i} - \mathbf{m}^{k-\text{Rep}} \big\rangle = \big\langle \mathbf{v}, \mathbf{m^i} - \sum_{k=1}^n \delta_k \mathbf{m}^{k-\text{Rep}} \big\rangle \\
&\overset{\text{Eq. (22)}}{=} \Big\langle \mathbf{v}, \sum_{k=1}^n \alpha_k\, \mathbf{g}^i(k) + \sum_{k=1}^n \beta_k\, \mathbf{g}^{-i}(k) + \gamma\,\mathbf{1} \Big\rangle = \sum_{k=1}^n \alpha_k \,\langle \mathbf{v}, \mathbf{g}^i(k)\rangle + \sum_{k=1}^n \beta_k \langle \mathbf{v}, \mathbf{g}^{-i}(k)\rangle + \gamma\,\langle \mathbf{v}, \mathbf{1}\rangle \\
&\overset{(17),(18)}{=} \hat{\alpha}\,\pi^i(\mathbf{m^i},\sigma^{-i}) + \hat{\beta}\,\pi^{-i}(\mathbf{m^i},\sigma^{-i}) + \gamma.
\end{aligned} \qquad [23]$$

That is, a player with a zero-determinant strategy enforces a linear relationship between the players' payoffs, irrespective of the co-player's strategy. Importantly, the parameters $\hat{\alpha}$, $\hat{\beta}$, and γ of that linear relationship are entirely under player i's control.

Generalized versions of Tit-for-tat. Suppose we choose $\boldsymbol{\alpha},\boldsymbol{\beta},\boldsymbol{\delta}$ such that $\alpha_k=-\beta_k=1/(T-S)$, $\delta_k=1$ for some k, and $\alpha_j=\beta_j=\delta_j=0$ for $j\neq k$. Moreover, set $\gamma=0$. In that case, formula Eq. (22) yields the strategy with entries

$$m_{\mathbf{h}} = \begin{cases} 1 & \text{if } a^{-i}_{-k} = C \\ 0 & \text{if } a^{-i}_{-k} = D \end{cases}$$

Therefore, a player with that strategy cooperates if and only if the co-player cooperated k rounds ago. Thus, the strategy implements `TFT` (for $k=1$) or delayed versions thereof (for $k>1$). By Eq. (23), the strategy enforces equal payoffs against any co-player,

$$\pi^i(\mathbf{m^i},\sigma^{-i}) = \pi^{-i}(\mathbf{m^i},\sigma^{-i}). \qquad [24]$$

Moreover, this strategy is nice if we additionally require it to unconditionally cooperate during the first k rounds. Given this additional requirement, the payoff of $\mathbf{m^i}$ against itself is R. Moreover, the strategy is a Nash equilibrium. To see why, suppose to the contrary that there is a strategy σ^{-i} with $\pi^{-i}(\mathbf{m^i},\sigma^{-i}) > R$. Then it follows from Eq. (24) that $\pi^i(\mathbf{m^i},\sigma^{-i})+\pi^{-i}(\mathbf{m^i},\sigma^{-i}) > 2R$. That is, the total payoff per round exceeds $2R$, which is incompatible with the basic assumptions on a prisoner's dilemma, Eq. (2). We conclude that all these versions of `TFT` are nice and they are Nash equilibria. Hence, they are partner strategies.

Generalized versions of Generous Tit-for-Tat. Another interesting special case arises in the donation game if $\boldsymbol{\alpha}=\mathbf{0}$, $\gamma=1-c/b$, and there is a k such that $\beta_k=-1/b$, $\delta_k=1$ and $\beta_j=\delta_j=0$ for $j\neq k$. In that case Eq. (22) yields the strategy

$$m_{\mathbf{h}} = \begin{cases} 1 & \text{if } \ a_{-k}^{-i}=C \\ 1-c/b & \text{if } \ a_{-k}^{-i}=D \end{cases}$$

The generated strategy is `GTFT` (if $k=1$), or a delayed version thereof (for $k>1$). By Eq. (23), the enforced payoff relationship is $\pi^{-i}(\mathbf{m^i},\sigma^{-i})=b-c$. That is, the co-player always obtains the mutual cooperation payoff, irrespective of the co-player's strategy. In particular, all these versions of `GTFT` are Nash equilibria (independent of how they act during the first n rounds). If we additionally require them to cooperate during the first n rounds, they are also nice. Hence, they are partner strategies.

Equalizers. We can further generalize the previous insight that a strategy like Generous Tit-for-Tat (and delayed versions thereof) can unilaterally determine the co-player's payoff. To this end, consider the donation game and let $\boldsymbol{\delta}$ be a non-negative distribution such that $\delta_k\geq 0$ for all k, and $\sum_{k=1}^{n}\delta_i=1$. Moreover, let $\kappa\in[0,1]$ be a parameter, and define

$$\boldsymbol{\alpha}=0, \qquad \boldsymbol{\beta}=-\frac{1}{b}\boldsymbol{\delta}, \qquad \gamma=\kappa(1-c/b). \qquad [25]$$

By Eq. (23), the enforced payoff relationship is now $\pi^{-i}(\mathbf{m^i},\sigma^{-i})=\kappa(b-c)$. That is, a player with a zero-determinant strategy that satisfies Eq. (25) sets the co-player's payoff to be a fixed number between 0 (for $\kappa=0$) and $b-c$ (for $\kappa=1$). In line with the previous literature, we refer to such a strategy as an *equalizer* (12, 24). The generalized versions of Generous Tit-for-Tat are a special case with $\kappa=1$ and $\boldsymbol{\delta}$ being a distribution that puts full weight on a single round k. By keeping $\kappa=1$ but varying the distribution $\boldsymbol{\delta}$, we obtain other equalizer strategies that fix the co-player's payoff to be $b-c$. Assuming these strategies prescribe to cooperate during the first n rounds, they all represent linear combinations of the various delayed versions of `GTFT`. All these linear combinationsl are partner strategies.

C. An algorithm to check whether a reactive-n strategy is a Nash equilibrium.

Sufficiency of checking pure self-reactive strategies. After discussing these particular cases, we aim to derive a general algorithm that allows us to verify whether a given reactive-n strategy is a Nash equilibrium. In principle, this requires us to check the payoff of any other strategy (including strategies that have a much longer memory length than n). Fortunately, however, some simplifications are possible when we use an insight by Press and Dyson (12). They discussed the case where one player uses a memory-1 strategy and the other player employs a longer memory strategy. They demonstrated that the payoff of the player with the longer memory is exactly the same as if the player had employed a specific shorter-memory strategy, disregarding any history beyond what is shared with the short-memory player. Here we show a similar result. If there is a part of the game's history that one player does not take into account, then the co-player gains nothing by considering that part of the history.

Lemma 2 (Against reactive strategies, any feasible payoff can be generated with self-reactive strategies)
Let $\mathbf{p}\in\mathcal{R}_n$ be a reactive strategy for player 2. Moreover, suppose player 1 adopts some strategy σ such that for the resulting game, the time average $\mathbf{v}$ according to Eq. (14) *exists. Then there is a self-reactive-n strategy $\tilde{\mathbf{p}}\in\mathcal{S}_n$ such that $\pi^i(\sigma,\mathbf{p})=\pi^i(\tilde{\mathbf{p}},\mathbf{p})$ for $i\in\{1,2\}$.*

For an illustration of this result, see Figure S1. It shows that against a reactive-2 player, any payoff that can be achieved with a memory-2 strategy can already be achieved with a self-reactive-2 strategy.

If we are to verify that some given reactive-n strategy $\mathbf{p}$ is a Nash equilibrium, Lemma 2 simplifies our task considerably. Instead of checking condition Eq. (21) for all possible strategies σ', we only need to check it for all self-reactive strategies $\tilde{\mathbf{p}}\in\mathcal{S}_n$. The following result simplifies our task even further.

Theorem 1 (To any reactive strategy, there is a best response among the pure self-reactive strategies)
For any reactive strategy $\mathbf{p}\in\mathcal{R}_n$ there is some pure self-reactive strategy $\tilde{\mathbf{p}}\in\mathcal{S}_n$ such that

$$\pi^1(\tilde{\mathbf{p}},\mathbf{p})\geq\pi^1(\sigma',\mathbf{p}) \quad \textit{for all other strategies } \sigma' \textit{ for which the limit } \text{Eq. (14)} \textit{ exists.} \qquad [26]$$

This result implies that we only need to check finitely many other strategies if we are to verify that some given reactive-n strategy is a Nash equilibrium.

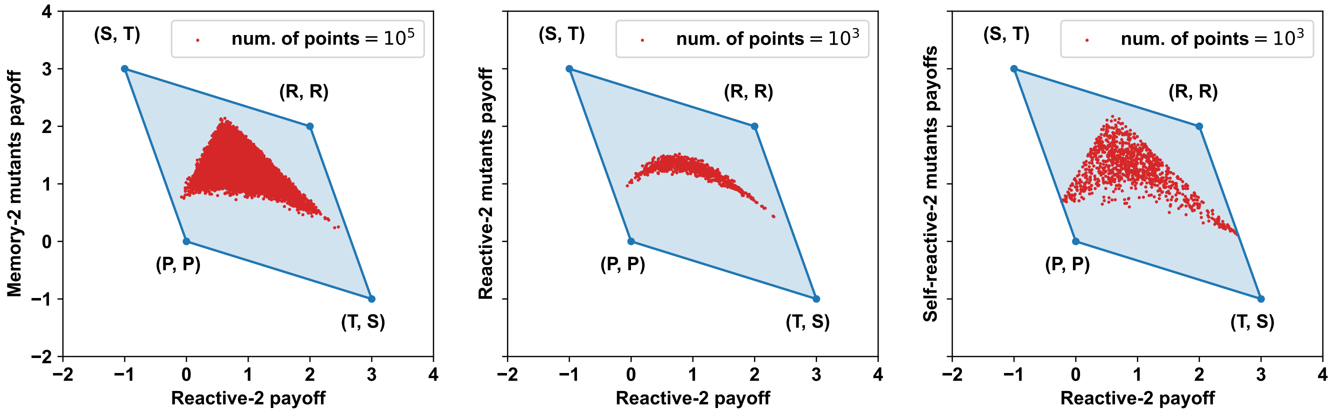

Fig. S1. Feasible payoffs for a reactive-2 strategy. We consider a player with reactive-2 strategy $\mathbf{p} = (0.37, 0.89, 0.95, 0.23)$. The player interacts with many other players (referred to as 'mutants') who adopt either some random memory-2 strategy (left), a random reactive-2 strategy (middle), or a random self-reactive-2 strategy (right panel). The panels show the resulting payoffs to the two players as red dots, with the x-axis showing the payoff of the focal player, and the y-axis showing the payoff of the mutants. We observe that when mutants use memory-2 strategies and self-reactive-2 strategies, we obtain the same region of feasible payoffs, in line with Lemma 2. In contrast, if mutants are restricted to reactive-2 strategies, the set of feasible payoffs is strictly smaller. Here, we consider a donation game with $b = 3$ and $c = 1$.

Algorithm 1 An algorithm to verify whether a given reactive strategy $\mathbf{p}$ is a Nash equilibrium.

input : $\mathbf{p}, n$

pure_self_reactive_strategies $\leftarrow \left\{ \tilde{\mathbf{p}} \;\middle|\; \tilde{\mathbf{p}} \in \{0,1\}^{2^n} \right\}$

isNash $\leftarrow$ True

for $\tilde{\mathbf{p}} \in$ *pure_self_reactive_strategies* **do**

  if $\pi^1(\mathbf{p},\mathbf{p}) < \pi^1(\tilde{\mathbf{p}},\mathbf{p})$ **then**

    isNash $\leftarrow$ False

return ($\mathbf{p}$, isNash)

Corollary 1 (An algorithm to check whether a reactive-n strategy is a Nash equilibrum)
A reactive strategy $\mathbf{p} \in \mathcal{R}_n$ *is a Nash equilibrium if and only if* $\pi^1(\mathbf{p},\mathbf{p}) \geq \pi^1(\tilde{\mathbf{p}},\mathbf{p})$ *for all pure self-reactive strategies* $\tilde{\mathbf{p}} \in \mathcal{S}_n$.

Corollary 1 gives us a straightforward procedure to check whether a given reactive strategy $\mathbf{p}$ is a Nash equilibrium (for a depiction, see Algorithm 1). To verify that $\mathbf{p}$ is a Nash equilibrium, we merely need to compare its payoff against itself to the payoff of a deviation towards one of the 2^{2^n} pure self-reactive strategies.

A more efficient way to calculate payoffs. For the remainder of this section, we thus assume that player 1 uses a self-reactive-n strategy $\tilde{\mathbf{p}} = (\tilde{p}_{\mathbf{h}^i})_{\mathbf{h}^i \in H^i}$, whereas player 2 uses a reactive-n strategy $\mathbf{p} = (p_{\mathbf{h}^{-i}})_{\mathbf{h}^{-i} \in H^{-i}}$. Our algorithm to compute payoffs for the two players in Section 2C would require us to interpret the two strategies as memory-n strategies. We would thus compute a left eigenvector of a $2^{2n} \times 2^{2n}$ transition matrix. In the following, however, we show that for games between reactive and self-reactive players, it suffices to consider a $2^n \times 2^n$ transition matrix. This efficiency gain is possible because both players only consider player 1's past actions. Instead of taking the space of all of the game's n-histories $H = H^1 \times H^2$ as the state space, we can thus take the space H^1. Let $h^1 = (a^1_{-n}, \ldots, a^1_{-1})$ be the state in the current round. Then we obtain the following probability that the state after one round is $\tilde{h}^1 = (\tilde{a}^1_{-n}, \ldots, \tilde{a}^1_{-1})$,

$$\tilde{M}_{\mathbf{h}^1,\tilde{\mathbf{h}}^1} = \begin{cases} \tilde{p}_{\mathbf{h}^1} & \text{if } \tilde{a}^1_{-1} = C, \text{ and } \tilde{a}^1_{-t} = a^1_{-t+1} \text{ for all } t \in \{2,\ldots,n\} \\ 1 - \tilde{p}_{\mathbf{h}^1} & \text{if } \tilde{a}^1_{-1} = D, \text{ and } \tilde{a}^1_{-t} = a^1_{-t+1} \text{ for all } t \in \{2,\ldots,n\} \\ 0 & \text{if } \tilde{a}^1_{-t} \neq a^1_{-t+1} \text{ for some } t \in \{2,\ldots,n\}. \end{cases} \qquad [27]$$

Similar to the vector $\mathbf{v}$ for matrix M, let $\tilde{\mathbf{v}} = (\tilde{v}_{\mathbf{h}^1})_{\mathbf{h}^1 \in H^1}$ be the limiting distribution of the dynamics defined by $\tilde{M}$ (which only in exceptional cases depends on player 1's behavior during the first n rounds). Then the players' payoffs are given by

$$\begin{aligned} \pi^1(\tilde{\mathbf{p}},\mathbf{p}) &= \sum_{\mathbf{h}^1 \in H^1} \tilde{v}_{\mathbf{h}^1} \Big(\tilde{\mathbf{p}}_{\mathbf{h}^1} \mathbf{p}_{\mathbf{h}^1} \cdot R + \tilde{\mathbf{p}}_{\mathbf{h}^1} (1 - \mathbf{p}_{\mathbf{h}^1}) \cdot S + (1 - \tilde{\mathbf{p}}_{\mathbf{h}^1}) \mathbf{p}_{\mathbf{h}^1} \cdot T + (1 - \tilde{\mathbf{p}}_{\mathbf{h}^1})(1 - \mathbf{p}_{\mathbf{h}^1}) \cdot P \Big), \\ \pi^2(\tilde{\mathbf{p}},\mathbf{p}) &= \sum_{\mathbf{h}^1 \in H^1} \tilde{v}_{\mathbf{h}^1} \Big(\tilde{\mathbf{p}}_{\mathbf{h}^1} \mathbf{p}_{\mathbf{h}^1} \cdot R + \tilde{\mathbf{p}}_{\mathbf{h}^1} (1 - \mathbf{p}_{\mathbf{h}^1}) \cdot T + (1 - \tilde{\mathbf{p}}_{\mathbf{h}^1}) \mathbf{p}_{\mathbf{h}^1} \cdot S + (1 - \tilde{\mathbf{p}}_{\mathbf{h}^1})(1 - \mathbf{p}_{\mathbf{h}^1}) \cdot P \Big). \end{aligned} \qquad [28]$$

Example: Payoffs and best responses with one-round memory. To illustrate the above results, we consider the case $n=1$. Assume player 1's self-reactive strategy is $\mathbf{\tilde{p}^1}=(\tilde{p}^1_C,\tilde{p}^1_D)$ and player 2's reactive strategy is $\mathbf{p^2}=(p^2_C,p^2_D)$. If we use the algorithm in Section 2C, we first formally represent these strategies as memory-1 strategies, $\mathbf{m^1}=(\tilde{p}^1_C,\tilde{p}^1_C,\tilde{p}^1_D,\tilde{p}^1_D)$ and $\mathbf{m^2}=(p^2_C,p^2_D,p^2_C,p^2_D)$. The respective transition matrix according to Eq. (12) is

$$M=\begin{pmatrix} \tilde{p}^1_C p^2_C & \tilde{p}^1_C(1-p^2_C) & (1-\tilde{p}^1_C)p^2_C & (1-\tilde{p}^1_C)(1-p^2_C)\\ \tilde{p}^1_C p^2_C & \tilde{p}^1_C(1-p^2_C) & (1-\tilde{p}^1_C)p^2_C & (1-\tilde{p}^1_C)(1-p^2_C)\\ \tilde{p}^1_D p^2_D & \tilde{p}^1_D(1-p^2_D) & (1-\tilde{p}^1_D)p^2_D & (1-\tilde{p}^1_D)(1-p^2_D)\\ \tilde{p}^1_D p^2_D & \tilde{p}^1_D(1-p^2_D) & (1-\tilde{p}^1_D)p^2_D & (1-\tilde{p}^1_D)(1-p^2_D) \end{pmatrix}. \qquad [29]$$

Assuming player 1's strategy is different from the one-round repeat strategy, $\mathbf{\tilde{p}^1}\neq(1,0)$, this transition matrix has a unique invariant distribution,

$$\mathbf{v}=\left(\frac{\tilde{p}^1_D\left(\tilde{p}^1_C(p^2_C-p^2_D)+p^2_D\right)}{1-(\tilde{p}^1_C-\tilde{p}^1_D)},\frac{\tilde{p}^1_D\left(1-\tilde{p}^1_C(p^2_C-p^2_D)-p^2_D\right)}{1-(\tilde{p}^1_C-\tilde{p}^1_D)},\frac{(1-\tilde{p}^1_C)\left(\tilde{p}^1_D(p^2_C-p^2_D)+p^2_D\right)}{1-(\tilde{p}^1_C-\tilde{p}^1_D)},\frac{(1-\tilde{p}^1_C)\left(1-\tilde{p}^1_D(p^2_C-p^2_D)-p^2_D\right)}{1-(\tilde{p}^1_C-\tilde{p}^1_D)}\right).$$

According to Eq. (16), Player 1's payoff is the scalar product

$$\pi^1(\mathbf{\tilde{p}^1},\mathbf{p^2})=\big\langle \mathbf{v}\,,(R,S,T,P)\big\rangle. \qquad [30]$$

Following Corollary 1, we can use these observations to characterize under which conditions a nice reactive strategy $\mathbf{p^2}=(1,p^2_D)$ is a partner. To this end, we compute player 1's payoff for all pure self-reactive strategies $\mathbf{\tilde{p}^1}=(\tilde{p}^1_C,\tilde{p}^1_D)$. These are `ALLC` $=(1,1)$, `ALLD` $=(0,0)$, and `Alternator` $=(0,1)$; we can ignore the one-round repeat strategy (1,0), because depending on the strategy's first round-behavior it is either equivalent to `ALLC` or to `ALLD`. The payoffs of these three strategies are

$$\begin{aligned} \pi^1(\texttt{ALLC},\mathbf{p^2}) &= R,\\ \pi^1(\texttt{ALLD},\mathbf{p^2}) &= p^2_D\cdot T+(1-p^2_D)\cdot P \\ \pi^1(\texttt{Alternator},\mathbf{p^2}) &= p^2_D/2\cdot R+(1-p^2_D)/2\cdot S+1/2\cdot T. \end{aligned} \qquad [31]$$

We conclude that player 2's reactive strategy $\mathbf{p^2}$ is a Nash equilibrium (and hence a partner) if none of these three payoffs exceeds the mutual cooperation payoff R. This requirement yields the condition

$$p^2_D\leq \min\left\{1-(T-R)/(R-S),\ (R-P)/(T-P)\right\}. \qquad [32]$$

As one may expect, $\mathbf{p^2}$ is a partner if and only if its generosity p^2_D does not exceed the generosity of `GTFT`, as defined by Eq. (8).

Instead of computing the 4×4 matrix M in Eq. (29), we could also consider the simplified 2×2 transition matrix Eq. (27). Here, the two possible states are $\mathbf{h^1}\in\{C,D\}$, and hence the matrix is

$$\tilde{M}=\begin{pmatrix}\tilde{p}^1_C & 1-\tilde{p}^1_C\\ \tilde{p}^1_D & 1-\tilde{p}^1_D\end{pmatrix}. \qquad [33]$$

Again, for $\mathbf{\tilde{p}^1}\neq(1,0)$, this transition matrix has a unique invariant distribution,

$$\mathbf{\tilde{v}}=(\tilde{v}_C,\tilde{v}_D)=\left(\frac{\tilde{p}^1_D}{1-(\tilde{p}^1_C-\tilde{p}^1_D)},\frac{1-\tilde{p}^1_C}{1-(\tilde{p}^1_C-\tilde{p}^1_D)}\right). \qquad [34]$$

If we take this invariant distribution and compute player 1's payoff according to Eq. (28), we recover the same expression as in Eq. (30), as expected.

D. Reactive partner strategies in the donation game. Just as in the previous example with $n=1$, we can use the results of the previous section to characterize the partner strategies for reactive-2 and reactive 3-strategies. For simplicity, we first consider the case of the donation game. Results for the general prisoner's dilemma follow in the next section.

Reactive-2 partner strategies. We first consider the case $n=2$. The resulting reactive-2 strategies can be represented as a vector $\mathbf{p}=(p_{CC},p_{CD},p_{DC},p_{DD})$. The entries $p_{\mathbf{h^{-i}}}$ are the player's cooperation probability, depending on the co-player's actions in the previous two rounds, $\mathbf{h^{-i}}=(a^{-i}_{-2},a^{-i}_{-1})$. For the strategy to be nice, we require $p_{CC}=1$. Based on Corollary 1, we obtain the following characterization of partners.

Theorem 2 (Reactive-2 partner strategies in the donation game)
A nice reactive-2 strategy $\mathbf{p}$*, is a partner strategy if and only if its entries satisfy the conditions*

$$p_{CC}=1,\qquad \frac{p_{CD}+p_{DC}}{2}\leq 1-\frac{1}{2}\cdot\frac{c}{b},\qquad p_{DD}\leq 1-\frac{c}{b}. \qquad [35]$$

The resulting conditions can be interpreted as follows: For each time a co-player has defected during the past two rounds, the reactive player's cooperation probability needs to decrease by $c/(2b)$. This reduced cooperation probability is sufficient to incentivize the co-player to cooperate. Interestingly, for the strategy to be a partner, the middle condition in Eq. (35) suggests that the exact timing of a co-player's defection is irrelevant. As long as *on average*, the respective cooperation probabilities p_{CD} and p_{DC} are below the required threshold $1-c/(2b)$, the strategy is a Nash equilibrium.

Reactive-3 partner strategies. Next, we focus on the case $n=3$. Reactive-3 strategies can be represented as a vector

$$\mathbf{p} = (p_{CCC}, p_{CCD}, p_{CDC}, p_{CDD}, p_{DCC}, p_{DCD}, p_{DDC}, p_{DDD}).$$

Again, each entry $p_{\mathbf{h^{-i}}}$ refers to the player's cooperation probability, depending on the co-player's previous three actions, $\mathbf{h^{-i}}=(a^{-i}_{-3}, a^{-i}_{-2}, a^{-i}_{-1})$. For the respective partner strategies, we obtain the following characterization.

Theorem 3 (Reactive-3 partner strategies in the donation game)
A nice reactive-3 strategy $\mathbf{p}$ *is a partner strategy if and only if its entries satisfy the conditions*

$$\begin{aligned} p_{CCC} &= 1 \\ \frac{p_{CDC}+p_{DCD}}{2} &\le 1-\frac{1}{2}\cdot\frac{c}{b} \\ \frac{p_{CCD}+p_{CDC}+p_{DCC}}{3} &\le 1-\frac{1}{3}\cdot\frac{c}{b} \\ \frac{p_{CDD}+p_{DCD}+p_{DDC}}{3} &\le 1-\frac{2}{3}\cdot\frac{c}{b} \\ \frac{p_{CCD}+p_{CDD}+p_{DCC}+p_{DDC}}{4} &\le 1-\frac{1}{2}\cdot\frac{c}{b} \\ p_{DDD} &\le 1-\frac{c}{b} \end{aligned} \qquad [36]$$

Compared to the case of reactive-2 strategies, the respective conditions are now somewhat less intuitive. However, there is still a general pattern that is preserved. On average, a player's cooperation probability needs to be diminished in proportion to the number of defections in memory. More specifically, in a given inequality, let x be the proportion of D's in the histories on the left-hand's side. Then the threshold on the right-hand side is given by $1-x\cdot c/b$. For example, in case of the second inequality in Eq. (36), the left-hand side features the histories CCD, CDC, DCC. The proportion of D's across all three histories is $1/3$. Therefore, the threshold on the right-hand side is $1-1/3{\cdot}c/b$.

Because reactive-2 strategies are a subset of reactive-3 strategies, we can use the conditions in Eq. (36) to recover the conditions in Eq. (35). For example, because reactive-2 strategies ignore what happened more than two rounds ago, they satisfy $p_{CDC}=p_{DDC}$. When we plug this equation into the first inequality in Eq. (36), we obtain

$$\frac{p_{CCD}+p_{CDC}}{2} \le 1-\frac{1}{2}\cdot\frac{c}{b} \qquad \text{and} \qquad \frac{p_{DCD}+p_{DDC}}{2} \le 1-\frac{1}{2}\cdot\frac{c}{b} \qquad [37]$$

That is, irrespective of what happened three rounds ago, the partners among the reactive-3 strategies satisfy the first inequality in Eq. (35). Similarly, the second inequality in Eq. (35) follows from the last inequality Eq. (36).

Partners among the reactive-n counting strategies.. Using the same methods as before, one can in principle also characterize the partners among the reactive-4 and the reactive-5 strategies. However, the respective conditions quickly become unwieldy. However, in the special case of *reactive-n counting strategies*, we can provide a simple condition for any memory length. To formally define these strategies, let $|\mathbf{h^{-i}}|$ denote the number of C's in a given n-history of the co-player. We say a reactive-n strategy $\mathbf{p}=(p_{h^{-i}})_{\mathbf{h^{-i}}\in\mathbf{H^{-i}}}$ is a counting strategy if

$$|\mathbf{h}^{-i}|=|\tilde{\mathbf{h}}^{-\mathbf{i}}| \quad \Rightarrow \quad p_{\mathbf{h^{-i}}} = p_{\tilde{\mathbf{h}}^{-\mathbf{i}}}. \qquad [38]$$

That is, the reactive player's cooperation probability only depends on the number of cooperative acts during the past n rounds and not on their timing. Such reactive-n counting strategies can be written as $n{+}1$-dimensional vectors $\mathbf{r}=(r_k)_{k\in\{n,\dots,0\}}$. Here, r_k is the player's cooperation probability, given the co-player cooperated k times during the past n rounds.

For example, for $n=2$, counting strategies are those reactive-2 strategies for which $p_{CD}=p_{DC}$. In that case, we associate $r_2=p_{CC}$, $r_1=p_{CD}=p_{DC}$, and $r_0=p_{DD}$. Within the set of reactive-n counting strategies, we get the following simple characterization of partners.

Theorem 4 (Partners among the reactive-n counting strategies)
A nice reactive-n counting strategy $\mathbf{r}=(r_k)_{k\in\{n,n-1,\dots,0\}}$, *is a partner strategy if and only if*

$$r_n=1 \qquad \textit{and} \qquad r_{n-k}\le 1-\frac{k}{n}\cdot\frac{c}{b} \quad \text{for} \quad k\in\{1,2,\dots,n\}. \qquad [39]$$

Here, the interpretation is straightforward. For each defection, the player's cooperation probability decreases by $c/(nb)$.

Note that for $n\ge 2$, the space of reactive-n counting strategies is strictly smaller than the space of reactive-n strategies. For example, the space of reactive-2 strategies is 4-dimensional, whereas the respective space of counting strategies is 3-dimensional. However, in terms of memory required to implement the respective strategies, the two strategy spaces are equivalent. In each case, the player needs to memorize the exact sequence $\mathbf{h^{-i}}=(a^{-i}_{-n},\dots,a^{-i}_{-1})$ of the co-player's last n actions. In the case of reactive-n strategies this memory requirement is obvious. After all, players need to know the exact sequence $\mathbf{h^{-i}}$ to select

the appropriate cooperation probability $p_{\mathbf{h^{-i}}}$. But even for reactive-n counting strategies merely memorizing the co-player's cooperation count $k := |\mathbf{h}^{-i}| \in \{0, \ldots, n\}$ is not sufficient. This number would suffice to choose the correct cooperation probability r_k in the respective round. However, to correctly update the co-player's cooperation count $|\mathbf{h}^{-i}|$, the player still needs to remember which action the co-player chose n rounds ago (because that is the part of the n-history that will be dropped after the current round). A similar observation then applies to the next round, too: at that point, a player will need to remember exactly what happened (what is currently) $n-1$ rounds ago. By this logic, it follows that even a memory-n counting strategy requires a memory of the exact sequence of the co-player's last n actions, not a mere memory of how often the co-player cooperated during the past n rounds.

E. Reactive partner strategies in the general prisoner's dilemma. In the previous section, we have characterized the reactive partner strategies for the donation game. In the following, we apply the same methods based on Section 3C to analyze the general prisoner's dilemma. For the case of reactive-2 strategies, we obtain the following characterization.

Theorem 5 (Reactive-2 partner strategies in the prisoner's dilemma)
A nice reactive-2 strategy $\mathbf{p}$ *is a partner strategy if and only if its entries satisfy the conditions*

$$\begin{aligned} p_{CC} &= 1, \\ (T-P)\,p_{DD} &\leq R-P, \\ (R-S)\,(p_{CD}+p_{DC}) &\leq 3R-2S-T, \\ (T-P)\,p_{DC}+(R-S)\,p_{CD} &\leq 2R-S-P, \\ (T-P)\,(p_{CD}+p_{DC})+(R-S)\,p_{DD} &\leq 3R-S-2P, \\ (T-P)\,p_{CD}+(R-S)\,(p_{CD}+p_{DD}) &\leq 4R-2S-T-P. \end{aligned} \qquad [40]$$

Compared to the donation game, there are now more conditions, and these conditions are more difficult to interpret. Reassuringly, however, the conditions simplify to the conditions Eq. (35) in the special case that the payoff values satisfy $R=b-c$, $S=-c$, $T=b$, and $P=0$. For the case of reactive-3 strategies, the characterization is as follows.

Theorem 6 (Reactive-3 partner strategies in the prisoner's dilemma)
A nice reactive-3 strategy $\mathbf{p}$ *is a partner strategy if and only if its entries satisfy the conditions in Table S1.*

Given the large number of conditions in Table S1, we do not pursue a similar characterization for $n>3$, even though the same methods remain applicable.

F. Defector strategies in the donation game. In the previous sections, we considered partner strategies – those strategies that sustain full cooperation in a Nash equilibrium. For comparison, in the following we characterize equilibria in which players fully defect. In analogy to partner strategies, the corresponding *defector strategies* need to satisfy two properties. (i) A player with a defector strategy is never the first to cooperate. (ii) When both players adopt a defector strategy, the strategies form a Nash equilibrium. Based on our general framework in Section 3C, we can characterize defector strategies as follows.

Theorem 7 (Reactive-n defector strategies in the donation game)

1. *A reactive-1 strategy* $\mathbf{p}=(p_C, p_D)$ *is a defector strategy if and only if* $p_C \leq \frac{c}{b}$ *and* $p_D = 0$.

2. *A reactive-2 strategy* $\mathbf{p}=(p_{CC}, p_{CD}, p_{DC}, p_{DD})$ *is a defector strategy if and only if*

$$p_{CC} \leq \frac{c}{b}, \qquad \frac{p_{CD}+p_{DC}}{2} \leq \frac{c}{2b}, \qquad p_{DD} = 0. \qquad [41]$$

3. *A reactive-3 strategy* $\mathbf{p}$ *is a defector strategy if and only if*

$$\begin{aligned} p_{CCC} &\leq \frac{c}{b} \\ \frac{p_{CDC}+p_{DCD}}{2} &\leq \frac{1}{2}\cdot\frac{c}{b} \\ \frac{p_{CCD}+p_{CDC}+p_{DCC}}{3} &\leq \frac{2}{3}\cdot\frac{c}{b} \\ \frac{p_{CDD}+p_{DCD}+p_{DDC}}{3} &\leq \frac{1}{3}\cdot\frac{c}{b} \\ \frac{p_{CCD}+p_{CDD}+p_{DCC}+p_{DDC}}{4} &\leq \frac{1}{2}\cdot\frac{c}{b} \\ p_{DDD} &= 0. \end{aligned} \qquad [42]$$

$$
\begin{aligned}
p_{CCC} &= 1,\\
(T-P)\,(p_{CDD}+p_{DCD}+p_{DDC})+(R-S)\,p_{DDD} &\leq 4R{-}3P{-}S\\
(T-P)\,p_{CDC}+(R-S)\,p_{DCD} &\leq 2R{-}P{-}S\\
(T-P)\,p_{DDD} &\leq R{-}P\\
(T-P)\,(p_{CCD}+p_{CDD}+p_{DDC})+(R-S)\,(p_{CDC}+p_{DCC}+p_{DCD}+p_{DDD}) &\leq 8R{-}3P{-}4S{-}T\\
(T-P)\,p_{DCC}+(R-S)\,(p_{CCD}+p_{CDC}) &\leq 3R{-}P{-}2S\\
(T-P)\,(p_{CCD}+p_{DCC}+p_{DDC})+(R-S)\,(p_{CDC}+p_{CDD}+p_{DCD}) &\leq 6R{-}3P{-}3S\\
(T-P)\,(p_{CCD}+p_{DDC})+(R-S)\,(p_{CDC}+p_{CDD}+p_{DCC}+p_{DCD}) &\leq 7R{-}2P{-}4S{-}T\\
(T-P)\,(p_{CCD}+p_{CDD}+p_{DCC})+(R-S)\,(p_{DDC}+p_{DDD}) &\leq 5R{-}3P{-}2S\\
(T-P)\,(p_{DCD}+p_{DDC})+(R-S)\,p_{CDD} &\leq 3R{-}2P{-}S\\
(T-P)\,p_{CCD}+(R-S)\,(p_{CDD}+p_{DCC}+p_{DDC}) &\leq 5R{-}P{-}3S{-}T\\
(T-P)\,(p_{CCD}+p_{DCC})+(R-S)\,(p_{CDD}+p_{DDC}) &\leq 4R{-}2P{-}2S\\
(T-P)\,(p_{CDC}+p_{DCD})+(R-S)\,(p_{CCD}+p_{CDD}+p_{DCC}+p_{DDC}) &\leq 7R{-}2P{-}4S{-}T\\
(T-P)\,(p_{CDC}+p_{CDD}+p_{DCD})+(R-S)\,(p_{CCD}+p_{DCC}+p_{DDC}+p_{DDD}) &\leq 8R{-}3P{-}4S{-}T\\
(T-P)\,(p_{CDC}+p_{DCC}+p_{DCD})+(R-S)\,(p_{CCD}+p_{CDD}+p_{DDC}) &\leq 6R{-}3P{-}3S\\
(T-P)\,(p_{CCD}+p_{CDD}+p_{DCC}+p_{DDC})+(R-S)\,(p_{CDC}+p_{DCD}+p_{DDD}) &\leq 7R{-}4P{-}3S\\
(R-S)\,(p_{CCD}+p_{CDC}+p_{DCC}) &\leq 4R{-}3S{-}T\\
(T-P)\,(p_{CCD}+p_{CDD})+(R-S)\,(p_{DCC}+p_{DDC}+p_{DDD}) &\leq 6R{-}2P{-}3S{-}T\\
(T-P)\,(p_{CDC}+p_{CDD}+p_{DCC}+p_{DCD})+(R-S)\,(p_{CCD}+p_{DDC}+p_{DDD}) &\leq 7R{-}4P{-}3S
\end{aligned}
$$

Table S1. Necessary and sufficient conditions for a nice reactive-3 strategy to be a partner in the prisoner's dilemma.

The interpretation of these conditions is similar to before. In each case, a defector strategy's cooperation probability increases in proportion to the co-player's average cooperation rate during the last n rounds. Similar to the case of partner strategies, we can also identify the defector strategies among the reactive-n counting strategies $\mathbf{r} = (r_k)$. We obtain that a reactive-n counting strategy is a defector if and only if

$$r_k \leq \frac{k}{n} \cdot \frac{c}{b} \quad \text{for} \quad k \in \{0, 1, \ldots, n\}. \qquad [43]$$

G. Games with errors. So far, we have studied equilibrium behavior when actions are executed perfectly. Individuals who wished to cooperate always had the means to do so; individuals who wished to defect always did. In the following, we allow decisions to be subject to errors. There are three motivations to do so. First, a model with errors seems to be the more realistic approximation to the games played in real life, where decisions tend to be noisy. Second, empirical evidence suggests that individuals take error rates into account, and adapt their behavior accordingly (25, 26). Finally, the study of errors is of considerable theoretical interest. For example, it has been noted early on that errors can have a drastic effect on the effectiveness of classical strategies such as `TFT` (20, 27, 28). Similarly, errors have a considerable impact on whether cooperation can be evolutionarily stable at all (29–31). These reasons make an extension of our results to games with errors desirable.

At first, such an extension may appear straightforward. Indeed, many of our key results naturally carry over. For example, in order to explore whether a reactive-n strategy is a Nash equilibrium, it still suffices to compare it to all pure self-reactive-n strategies. Nevertheless, errors give rise to additional complications. In particular, they make it more difficult to obtain closed-form expressions for the payoff of a partner strategy against itself. Without errors, this payoff is simply $b-c$ (in the donation game), or R (in the general prisoner's dilemma). In games with errors, the expressions quickly become more complex. Already for relatively simple strategy spaces, like reactive-2 or memory-1 strategies, these complications render an analytical treatment difficult. For example, as of now the set of all Nash equilibria among the memory-1 strategies is only known for games without errors (17, 21). More recently, some progress has been possible in games with an alternating move structure (32). But for games in which players decide simultaneously (as considered herein), an analytical treatment remains challenging.

In the following, we extend our results into two directions. First, we characterize equilibrium strategies when error rates are positive, but vanishingly small. In a second step, we then characterize a subset of reactive-n equalizer strategies that form a Nash equilibrium for any error rate.

Implementation errors. In line with the existing literature (1–4), we focus on the effect of implementation errors. An individual who intends to cooperate instead defects with some probability ε. Conversely, an individual who intends to defect instead cooperates with the same probability. It is straightforward to include such errors into our model (see also Ref. 32). Once a

player i with some memory-n strategy $\mathbf{m^i} = (m^i_{\mathbf{h}})_{\mathbf{h}\in H}$ is subject to errors, the player acts as if it used a strategy

$$\mathbf{m^{i,\varepsilon}} = \varphi^{\varepsilon}(\mathbf{m^i}) := (1-\varepsilon)\mathbf{m^i} + \varepsilon(\mathbf{1} - \mathbf{m^i}). \quad [44]$$

The transformation φ^{ε} is a bijection that maps any *nominal strategy* $\mathbf{m} \in [0,1]^{2^{2n}}$ to an effective strategy $\mathbf{m}^{\varepsilon} \in [\varepsilon, 1-\varepsilon]^{2^{2n}}$. The inverse mapping is given by

$$\left(\varphi^{\varepsilon}\right)^{-1}(\mathbf{m}^{\varepsilon}) = \frac{\mathbf{m}^{\varepsilon} - \varepsilon \cdot \mathbf{1}}{1-2\varepsilon}. \quad [45]$$

In addition to $\mathbf{m}^{\varepsilon}$, we use an analogous notation $\mathbf{p}^{\varepsilon}$ and $\tilde{\mathbf{p}}^{\varepsilon}$ for reactive and self-reactive strategies, respectively. For example, in games with errors, the strategy ALLD is mapped to $\texttt{ALLD}^{\varepsilon} = (\varepsilon, \ldots, \varepsilon)$. Similarly, ALLC is mapped onto $\texttt{ALLC}^{\varepsilon} = (1-\varepsilon, \ldots, 1-\varepsilon)$. Finally, a strategy like Tit-for-two-Tat is mapped from $\mathbf{p} = (1,1,1,0)$ to $\mathbf{p}^{\varepsilon} = (1-\varepsilon, 1-\varepsilon, 1-\varepsilon, \varepsilon)$.

Games with errors can be analyzed the same way as games without errors. We only need to replace the players' nominal strategies with their effective strategies. For example, suppose we wish to compute payoffs when a player with nominal strategy $\mathbf{m^1}$ interacts with a co-player with strategy $\mathbf{m^2}$, in a game with error rate ε. Then we can use Eq. (4) to obtain

$$\pi^{i,\varepsilon}(\mathbf{m^1}, \mathbf{m^2}) := \pi^{i}(\mathbf{m^{1,\varepsilon}}, \mathbf{m^{2,\varepsilon}}). \quad [46]$$

A few remarks are in order.

Remark 1 (*i*) *On payoffs being well-defined.* Because all entries of $m^{1,\varepsilon}$ and $m^{2,\varepsilon}$ are strictly between 0 and 1 for $\varepsilon > 0$, the payoffs $\pi^{i,\varepsilon}(\mathbf{m^1}, \mathbf{m^2})$ are independent of the players' actions during the first n rounds. They only depend on m^1, m^2, and on the error rate ε.

(*ii*) *On the definition of partners.* For the case with errors, we define a memory-n strategy $\mathbf{m}$ to be self-cooperative if it yields the mutual cooperation payoff against itself as $\varepsilon \to 0$, i.e., when errors are rare. We say the strategy is a Nash equilibrium for the error rate ε if $\pi^{1,\varepsilon}(\mathbf{m}, \mathbf{m}) \geq \pi^{1,\varepsilon}(\mathbf{m'}, \mathbf{m})$ for all other memory-n strategies $\mathbf{m'}$. We note that because of the results of Press and Dyson (12) and Levinsky et al (33) the restriction that $\mathbf{m'}$ has the same memory length n imposes no restriction on the generality of this definition. We say a self-cooperative strategy is a partner for the error rate ε if it is a Nash equilibrium for that error rate.

(*iii*) *On the continuity of payoffs with respect to the error rate.* We note that in general, the payoffs $\pi^{i,\varepsilon}(\mathbf{m^1}, \mathbf{m^2})$ may be discontinuous as a function of ε at $\varepsilon = 0$. Such discontinuities can arise when the transition matrix M according to Eq. (12) has multiple absorbing states in the game without errors. In that case, the players' payoffs depend on their actions during the initial n rounds. The most prominent example of such a case occurs when two TFT players interact. Under the assumption that these players cooperate in the first round, we obtain for the donation game $\pi^1(\texttt{TFT}, \texttt{TFT}) = b-c$. That is, players achieve the mutual cooperation payoff. Once we allow for errors, however, we obtain $\pi^{1,\varepsilon}(\texttt{TFT}, \texttt{TFT}) = (b-c)/2$ even when error rates are vanishingly small, $\varepsilon \to 0$. Importantly, however, across all memory-n strategies these discontinuities in ε are rare. If the players' nominal strategies are such that the resulting transition matrix M has a unique absorbing state, the resulting payoff function $\pi^{i,\varepsilon}(\mathbf{m^1}, \mathbf{m^2})$ is continuous in ε at $\varepsilon = 0$.

(*iv*) *On the complexity of computing payoffs.* In general, it can be difficult to derive algebraic expressions for the players' payoffs when both use general reactive-n strategies. Already for $n = 2$, such a payoff computation requires us to derive an eigenvector of a 16×16 matrix. In the previous section without errors, we could bypass some of these complexities. For example, when a self-cooperative strategy $\mathbf{p} = (1, p_{CD}, p_{DC}, p_{DD})$ interacts with another, they cooperate indefinitely. Hence, the relevant eigenvector follows immediately. It is the 16-dimensional vector $\mathbf{v} = (v_{\mathbf{h}})$ with an entry of $v_{CC,CC} = 1$ and $v_{\mathbf{h}} = 0$ for all other histories $\mathbf{h}$. Unfortunately, this bypass is no longer available in games with errors. In that case, all of the 16 possible 2-histories $\mathbf{h}$ will be played with positive probability (in fact, that probability is bounded from below by ε^4, the probability that both players experience that history by a sequence of consecutive errors). It follows that for games with errors, we can no longer hope for a simple characterization of all Nash equilibria. To still make progress, in the following we use two distinct approaches. For the first approach, we weaken the equilibrium notion we use, by considering approximate Nash equilibria. For the second approach, we consider a subset of Nash equilibria for which rigorous results are feasible.

Approximate Nash equilibria in games with implementation errors. Instead of Nash equilibria, in the following we consider a slightly looser equilibrium concept. Specifically, we say a memory-n strategy $\mathbf{m}$ is an approximate Nash equilibrium for a threshold of $\Delta > 0$ and an error rate ε if

$$\pi^{1,\varepsilon}(\mathbf{m'}, \mathbf{m}) - \pi^{1,\varepsilon}(\mathbf{m}, \mathbf{m}) \leq \Delta \quad \text{for all } m' \in \mathcal{M}_n. \quad [47]$$

According to this definition, any payoff advantage that an alternative strategy $\mathbf{m'}$ might yield is bounded from above by Δ. For $\Delta \to 0$, this notion recovers the definition of a Nash equilibrium. For positive Δ, the set of approximate equilibria is a proper superset of the set of Nash equilibria. If $\mathbf{m}$ is self-cooperative and a proximate Nash equilibrium, we call it an *approximate partner strategy.*

Especially in the context of stochastic evolutionary dynamics (as in our paper), the concept of an approximate Nash equilibrium is still informative. To see why, consider a finite resident population that uses the strategy $\mathbf{m}$. Furthermore,

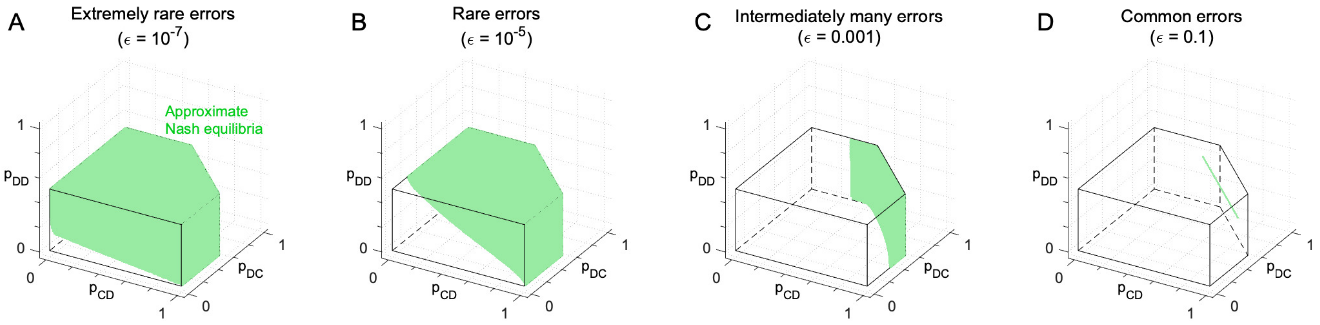

Fig. S2. The set of approximate Nash equilibria for varying error rates. We numerically compute the set of approximate Nash equilibria among the reactive-2 strategies for the donation game (using the same parameters as in Fig. 2C of the main text). To construct this figure, we use a finite grid of self-cooperative strategies $\mathbf{p}=(1,p_{CD},p_{DC},p_{DD})$. The entries p_{CD},p_{DC},p_{DD} are taken from the set $\{0,0.01,0.02,\ldots,1\}$. For each resulting strategy $\mathbf{p}$, we explore whether any pure self-reactive-2 strategy $\tilde{\mathbf{p}}$ satisfies $\pi^{1,\varepsilon}(\tilde{\mathbf{p}},\mathbf{p})-\pi^{1,\varepsilon}(\mathbf{p},\mathbf{p})>\Delta$. We use a comparably small value of $\Delta=0.001$, and we vary the error rate between $\varepsilon=10^{-7}$ and $\varepsilon=10^{-1}$. For sufficiently small error rates, the set of approximate partners for the game with errors approaches the set of partners for the game without errors (panel A). As we increase the error rate, the set of approximate partners approaches a line (panel D).

suppose this resident population is occasionally challenged by introducing a novel mutant strategy $\mathbf{m}'$. If $\mathbf{m}$ is a conventional Nash equilibrium, no mutant strategy has a selective advantage. Nevertheless, if the mutant strategy yields the same payoff as the resident, it may invade due to neutral drift (31). For approximate Nash equilibria, the situation is similar. Even if an advantageous mutant strategy $\mathbf{m}'$ appears, the initial dynamics will be well-approximated by neutral drift – provided the threshold Δ and the selection strength β are sufficiently small. In this sense, we may still expect approximate Nash equilibria to be played for a substantial amount of time during the evolutionary process.

For analytical purposes, the notion of approximate Nash equilibria is useful because it allows us to exploit the properties of continuous functions. If a strategy $\mathbf{m}$ is a Nash equilibrium of the game without errors, and payoffs are continuous in ε, then $\mathbf{m}$ remains an approximate Nash equilibrium for sufficiently small error rates. By this argument, and by Remark 1(*iii*), we immediately obtain the following result.

Theorem 8 (Approximate partners for games with errors)
Suppose $\mathbf{m}$ *is a partner strategy and suppose all entries of* $\mathbf{m}$ *are strictly positive. Then for any threshold* Δ *one can find a threshold* $\bar{\varepsilon}$ *such that* $\mathbf{m}$ *is an approximate partner for sufficiently small error rates,* $\varepsilon\leq\bar{\varepsilon}$.

For sufficiently small error rates, this result guarantees that almost all partner strategies identified in the previous section remain approximate partners once we allow for rare errors. In particular, the theorem captures all of the reactive-2 strategies in the interior of the polyhedron displayed in the main text Figure 2C,D. Only some boundary strategies such as TFT are excluded. At the same time, however, Theorem 8 does not quantify how small error rates need to be for a strategy $\mathbf{m}$ to be an approximate partner. To explore this issue in more detail, Figure S2 illustrates the set of approximate partners among the reactive-2 strategies for the donation game. As expected, the respective set approaches the polyhedron displayed in Figure 2C as errors become increasingly rare (Fig. S2A). However, as errors become more abundant, the set shrinks considerably. It approaches a line once $\varepsilon=0.1$. As we describe in the next section, this line corresponds to a line of equalizer strategies. In particular, we show that all these strategies are not merely approximate Nash equilibria; they are conventional Nash equilibria.

Equalizers for games with implementation errors. In Section 3B, we have introduced equalizers for the donation game without errors. Using Eqs. (22) and (25), we can write these equalizers as those memory-n strategies that can be written as

$$\mathbf{m}^{\mathbf{i}}=\sum_{k=1}^{n}\delta_k\left(-\frac{1}{b}\mathbf{g}^{-i}(k)+\mathbf{m}^{k-\mathrm{Rep}}\right)+\kappa(1-c/b)\,\mathbf{1}. \qquad [48]$$

Again, $\mathbf{g}^{i}(k)$ is the vector that returns player i's payoff k rounds ago, as defined by Eq. (15), $\mathbf{m}^{k-\mathrm{Rep}}$ is the memory-n strategy that repeats player i's own move k rounds ago, as defined by Eq. (11). Finally, $\boldsymbol{\delta}=(\delta_1,\ldots,\delta_n)$ satisfies $\sum_{k=1}^{n}\delta_k=1$. As shown in Section 3B, such strategies unilaterally impose a fixed payoff on the opponent, independent of the opponent's strategy σ^{-i},

$$\pi^{-i}(\mathbf{m}^{\mathbf{i}},\sigma^{-i})=\kappa(b-c). \qquad [49]$$

In particular, we concluded that any such strategy is a Nash equilibrium (there is no incentive to deviate because all deviations yield the same payoff).

In order to construct such equalizers for donation games with errors, we need to ensure that the player's *nominal* strategy is such that it gets mapped onto an *effective* equalizer strategy. By using the inverse mapping defined by Eq. (45), we conclude

that any strategy of the following form is a Nash equilibrium in a donation game with error rate ε,

$$\mathbf{m}^{\mathbf{i}} = \frac{1}{1-2\varepsilon}\left(\sum_{k=1}^{n}\delta_k\left(-\frac{1}{b}\mathbf{g}^{-i}(k)+\mathbf{m}^{k-\mathrm{Rep}}\right)+\Big(\kappa(1-c/b)-\varepsilon\Big)\,\mathbf{1}\right). \quad [50]$$

If we additionally require the strategy to be a partner, we need to ensure that it is nice as the error rate $\varepsilon \to 0$. This means that given a fully cooperative history $\mathbf{h_C}$, the strategy needs to ensure that the player would certainly cooperate in the absence of errors, $m^i_{\mathbf{h_C}}=1$. When we plug this condition into Eq. (50), we obtain

$$1 = m^i_{\mathbf{h_C}} = \frac{1}{1-2\varepsilon}\left(\sum_{k=1}^{n}\delta_k\left(-\frac{1}{b}(b-c)+1\right)+\Big(\kappa(1-c/b)-\varepsilon\Big)\right). \quad [51]$$

By noting that $\sum_{k=1}^{n}\delta_k=1$, we can solve this equation for κ, which yields $\kappa=(1-c/b-\varepsilon)/(1-c/b)$. By plugging this value of κ into Eq. (50), we conclude that for games with errors, all strategies of the following form are partner strategies,

$$\mathbf{m}^{\mathbf{i}} = \frac{1}{1-2\varepsilon}\left(\sum_{k=1}^{n}\delta_k\left(-\frac{1}{b}\mathbf{g}^{-i}(k)+\mathbf{m}^{k-\mathrm{Rep}}\right)+(1-c/b-2\varepsilon)\,\mathbf{1}\right). \quad [52]$$

Partners among the reactive-2 strategies. To illustrate this result, let us use Eq. (52) to identify the partners among the reactive-2 strategies, by setting $n=2$. First, it is straightforward to check that the memory-2 strategy defined by Eq. (52) is in fact a reactive-2 strategy. That is, the cooperation probabilities $m^i_{(\mathbf{h^i},\mathbf{h^{-i}})}$ do not depend on the focal player's own history $\mathbf{h^i}$. Next, let us consider a special case with $\delta_1=1$ and $\delta_2=0$. In that case, Eq. (52) implies

$$m^i_{(\mathbf{h^i},CC)} = m^i_{(\mathbf{h^i},DC)} = 1 \quad \text{and} \quad m^i_{(\mathbf{h^i},CD)} = m^i_{(\mathbf{h^i},DD)} = 1-\frac{c}{(1-2\varepsilon)b} \quad \text{for all } \mathbf{h^i}\in H^i. \quad [53]$$

Similarly, when we consider $\delta_1=0$ and $\delta_2=1$, we obtain

$$m^i_{(\mathbf{h^i},CC)} = m^i_{(\mathbf{h^i},CD)} = 1 \quad \text{and} \quad m^i_{(\mathbf{h^i},DC)} = m^i_{(\mathbf{h^i},DD)} = 1-\frac{c}{(1-2\varepsilon)b} \quad \text{for all } \mathbf{h^i}\in H^i. \quad [54]$$

That is, when we write these conditions as reactive-2 strategies $\mathbf{p}=(p_{CC},p_{CD},p_{DC},p_{DD})$, we recover a generalized version of Generous Tit-for-Tat, and another version with one round delay,

$$\texttt{GTFT} = \left(1, 1-\frac{c}{(1-2\varepsilon)b}, 1, 1-\frac{c}{(1-2\varepsilon)b}\right), \qquad \texttt{DGTFT} = \left(1, 1, 1-\frac{c}{(1-2\varepsilon)b}, 1-\frac{c}{(1-2\varepsilon)b}\right). \quad [55]$$

For general $\boldsymbol{\delta}=(\delta_1,\delta_2)$, we obtain linear combinations of these two strategies. For the numerical values $\varepsilon=0.1$, $b=2$, $c=1$, the two corner strategies become

$$\texttt{GTFT} = \big(1.000, 0.375, 1.000, 0.375\big), \qquad \texttt{DGTFT} = \big(1.000, 1.000, 0.375, 0.375\big). \quad [56]$$

These are exactly the end points of the green line depicted in panel D of Fig. S2. In particular, this figure panel suggests that except for the equalizers defined above, there are no other partner strategies within the set of reactive-2 strategies for games with errors. Whether the same is true for all reactive-n strategies, for general n, is an open question.

4. Further numerical results

In the following, we present some additional numerical results. In particular, we compute the volume of partner and defector strategies, we discuss an interesting asymmetry in our evolutionary simulations, we present simulation results for memory-n strategies, and we explore the role of implementation errors.

A. Volume of partner and defector strategies. In the previous section, we have mathematically characterized the set of partner and defector strategies. We can use the respective sizes of these two sets as a proxy for how likely cooperation is to evolve. To this end, following the work of Stewart and Plotkin (16), we compute the volume of the respective strategy sets. Specifically, to estimate the respective proportion of partners, we do the following: We ask: among all nice strategies, what is the proportion of strategies that additionally satisfy the conditions of being a Nash equilibrium? For $n \in \{1, 2, 3\}$, this condition can easily be calculated, given the respective characterizations in Eqs. (32), (35), (36) for reactive-n strategies, and Eq. (39) for reactive-n counting strategies. Analogously, we can derive the volume of defector strategies. To this end, we consider all strategies that defect with certainty after the co-player defected for n consecutive rounds (i.e., those strategies with $p_{\mathbf{h_D}} = 0$, where $\mathbf{h_D}$ is the n-history that only contains D). We ask which proportion of those strategies satisfy the respective conditions in Theorem 7. We compute these volumes with Monte Carlo simulations, by generating 10^4 random strategies with either $p_{\mathbf{h_C}} = 1$ or $p_{\mathbf{h_D}} = 0$, respectively, see Fig. S3. In this way, we get a quantitative measure for how abundant partners and defectors are for different memory lengths (we consider $n \in \{1, 2, 3\}$).

To get a first impression, we consider a donation game with a fixed cost-to-benefit ratio of $c/b = 0.7$ (Fig. S3A,D). Here, for one-round memory, defectors are more than twice as abundant as partners. As we increase the memory length to $n = 2$ and $n = 3$, the volume of both strategy sets shrinks, but the volume of defectors shrinks at a much higher rate. To understand why, it is intuitive to consider the conditions for reactive-n counting strategies, see Tab. S3. As this table shows, the two volumes become smaller because with every increase in n, there is an additional condition that needs to be satisfied. Importantly, however, for defectors, the conditions become more stringent with n. In contrast, for partners, all further conditions are increasingly easy to satisfy (all additional thresholds are above the first threshold of $r_0 \leq 0.3$).

As one may expect, these qualitative results are independent of the precise cost-to-benefit ratio. Of course, larger cost values increase the volume of defectors and reduce the volume of partners (Fig. S3B,E). However, for any given c/b value, we find that the volume of defectors shrinks more rapidly in n than the volume of partners. As introduced by Stewart and Plotkin (16), we can derive an important measure for the overall prospects of cooperation by computing the ratio of these two volumes. As shown in Fig. S3C,F, this relative proportion of partners increases in the players' memory capacity n. In particular, even for generally unfavorable conditions (such as $c/b > 0.5$), the relative proportion of partners can exceed one half if only n is sufficiently large. These results give some indication that with increasing memory, cooperation should evolve more easily.

B. Exploring an emerging asymmetry in the evolving strategies. In the main text, we report our evolutionary results in Fig. 4. There, we also observe an interesting asymmetry in the emerging strategies. For example, for reactive-2 strategies, we observe that the most abundant strategies tend to have $p_{CD} < p_{DC}$. These strategies are more likely to defect if a co-player defected in the previous round, rather than in the second-to-last round. One may interpret this as an indication that players are incentivized to punish a co-player's defection immediately, rather than with some delay. From a static perspective, this asymmetry is remarkable. After all, our conditions for reactive-2 partner strategies in Eq. (35) suggest that with respect to being a Nash equilibrium, the two entries p_{CD} and p_{DC} are interchangeable.

In order to explore this emerging asymmetry, it is again instructive to study a specific example. To this end, we consider the two reactive-2 strategies $\mathbf{p} = (1, 0.1, 0.6, 0.3)$ and $\mathbf{p}' = (1, 0.6, 0.1, 0.3)$. The two strategies are identical, except that the values of p_{CD} and p_{DC} are flipped. With respect to our conditions for partners, this difference is inconsequential: $\mathbf{p}$ is a partner for a given donation game if and only $\mathbf{p}'$ is a partner. To investigate whether the two strategies differ in their evolutionary properties, we explore whether they are equally robust with respect to invasions. To this end, we consider a homogeneous resident population that either adopts $\mathbf{p}$ or $\mathbf{p}'$. Then we simulate our evolutionary process by repeatedly introducing random mutant strategies. We record how many mutants need to be introduced on average until one of them replaces the resident. The simulations suggest that the two strategies $\mathbf{p}$ and $\mathbf{p}'$ are equivalent with respect to this metric, see Fig. S4A. In particular, the two strategies are equivalent when it comes to resist invasion by `ALLD` mutants (Fig. S4B).

The situation changes, however, once we take into account that in our simulations, resident populations do not implement perfect partner strategies. After all, according to our evolutionary process, mutant strategies are generated by randomly drawing cooperation probabilities in the interval [0,1]. As a result, we might occasionally see strategies with $p_{CC} > 0.99$, or even $p_{CC} > 0.999$, but we do not observe strategies with $p_{CC} = 1$ exactly. To take this kind of noise into account, we repeat our previous analysis, but now with resident strategies $\mathbf{p} = (0.99, 0.1, 0.6, 0.3)$ and $\mathbf{p}' = (0.99, 0.6, 0.1, 0.3)$, respectively. Those two strategies differ substantially with respect to their robustness against invasions, see Fig. S4C. Now, strategy $\mathbf{p}$ is considerably more robust ($\mathbf{p}$ is the strategy that tends to punish defection immediately). In particular, `ALLD` mutants are less likely to reach fixation in a homogeneous $\mathbf{p}$ population, compared to a homogeneous $\mathbf{p}'$ population, Fig. S4D.

The same panel, Fig. S4D, suggests the following key difference: When two $\mathbf{p}$-players interact in a repeated game, they get slightly higher payoffs than when two $\mathbf{p}'$-players interact. To analyze this difference in more detail, we consider the invariant distribution $\mathbf{v}$ in a game among two $\mathbf{p}$-players and among two $\mathbf{p}'$-players, respectively, see Fig. S5A. This invariant distribution indicates which states (2-histories) the two players typically visit over the course of the game. As one may expect, for both strategies the most visited state is the full cooperation history, (CC, CC). Interestingly, however, two $\mathbf{p}$-players tend to visit

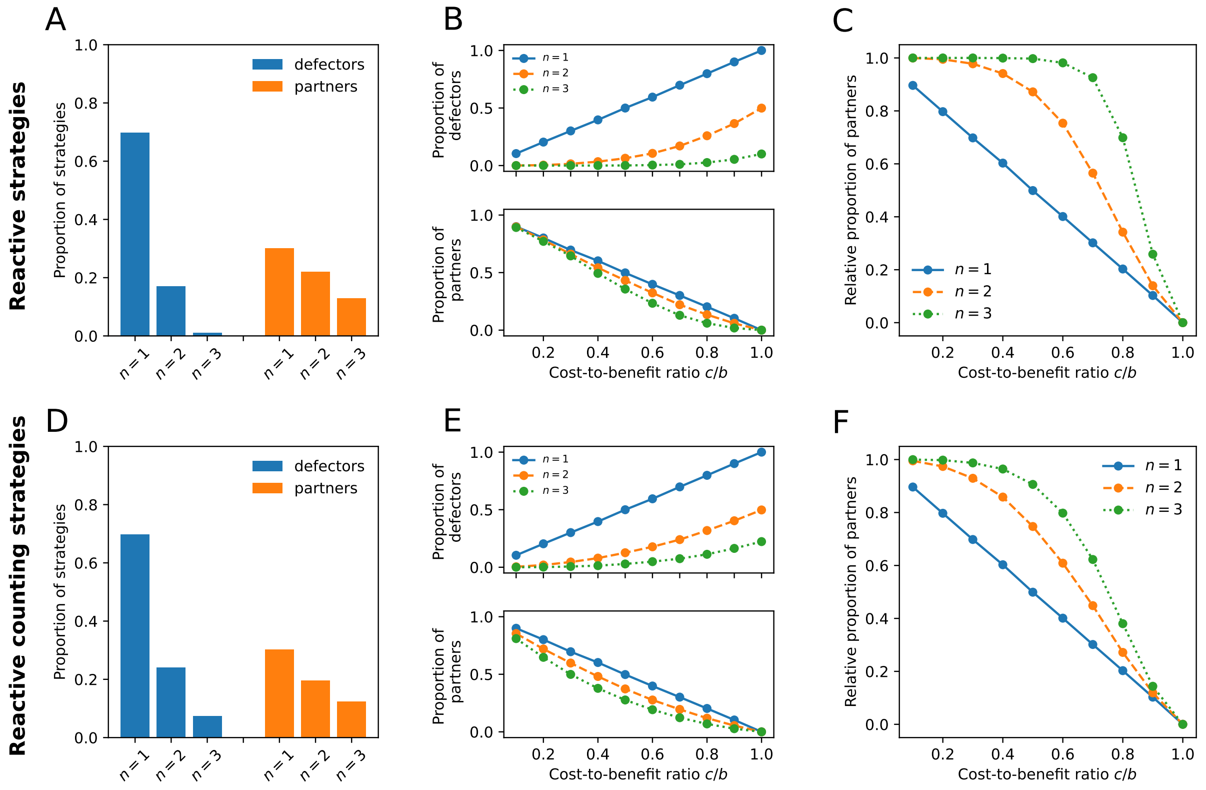

Fig. S3. Proportions of cooperative and defective Nash. For each strategy space, we draw 10^4 random strategies and create two copies of each one. For one copy, we set the cooperation probability after full cooperation of the co-player to one. For the second copy, we set the cooperation probability after full defection of the co-player to zero. We check whether the copies satisfy the properties of a partner or a defector, respectively. We consider reactive-n strategies (top) and reactive-n counting strategies (bottom), for $n \in \{1,2,3\}$. **A, D,** We plot the results for a fixed cost-to-benefit ratio $c/b = 0.5$. For $n=1$, defector strategies make up a larger proportion of the respective strategy space, compared to partners. This relationship is reversed for larger memory lengths. **B, E,** This qualitative result also holds for other cost-to-benefit ratios. In each case, the volume of defectors shrinks more rapidly in n than the volume of partners. **C, D,** As a simple measure for the likelihood of the evolution of cooperation, we plot the ratio of the two volumes (see also Ref. 16). We refer to this ratio as the relative proportion of partners. As we increase n, the relative proportion of partners increases, for all cost-to-benefit ratios.

**Memory capacity**	$n=1$	$n=2$	$n=3$
**Defectors**	$r_1 \leq \frac{c}{b} = 0.70$<br>*Volume: 70.0%*	$r_2 \leq \frac{c}{b} = 0.70$<br>$r_1 \leq \frac{1}{2}\frac{c}{b} = 0.35$<br>*Volume: 24.5%*	$r_3 \leq \frac{c}{b} = 0.70$<br>$r_2 \leq \frac{2}{3}\frac{c}{b} = 0.47$<br>$r_1 \leq \frac{1}{3}\frac{c}{b} = 0.23$<br>*Volume: 7.6%*
**Partners**	$r_0 \leq 1 - \frac{c}{b} = 0.30$<br>*Volume: 30.0%*	$r_0 \leq 1 - \frac{c}{b} = 0.30$<br>$r_1 \leq 1 - \frac{1}{2}\frac{c}{b} = 0.65$<br>*Volume: 19.5%*	$r_0 \leq 1 - \frac{c}{b} = 0.30$<br>$r_1 \leq 1 - \frac{2}{3}\frac{c}{b} = 0.53$<br>$r_2 \leq 1 - \frac{1}{3}\frac{c}{b} = 0.77$<br>*Volume: 12.3%*

Table S2. Volume of defectors and partners among the reactive-n counting strategies in a donation game with $b/c = 0.7$. The volume corresponds to the proportion of reactive-n counting strategies that satisfy all inequalities, among those strategies with $r_0 = 0$ (for defectors) and $r_n = 1$ (for partners).

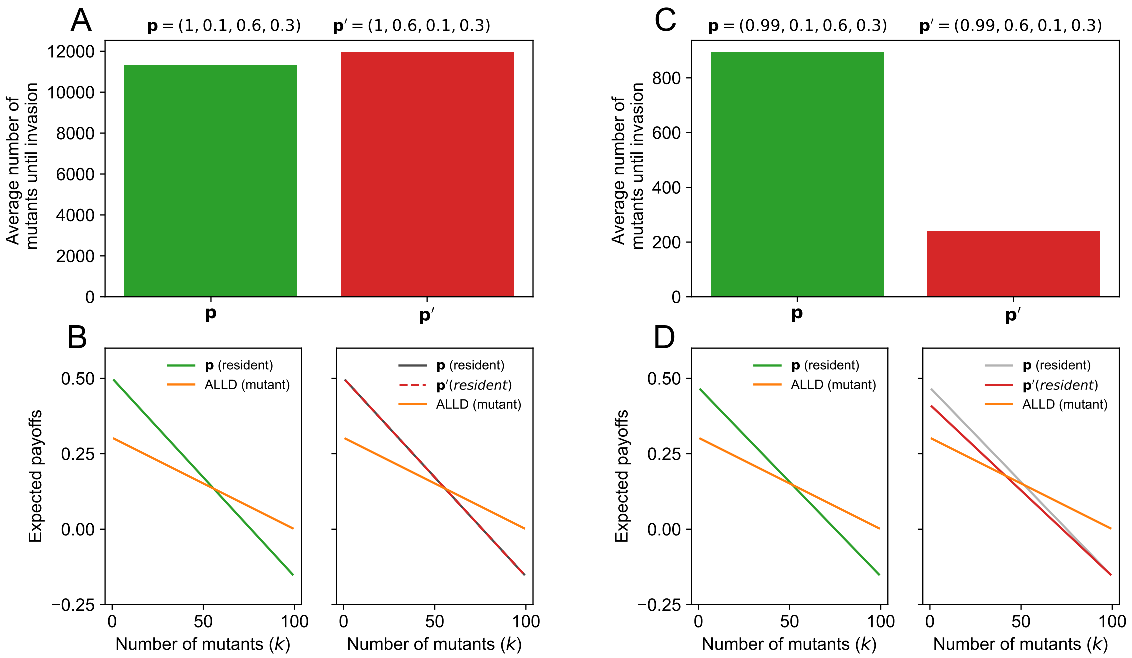

Fig. S4. An invasion analysis for reactive-2 strategies when $\mathbf{p}_{\mathbf{CD}}$ and $\mathbf{p}_{\mathbf{DC}}$ are reversed. A, We study the evolutionary dynamics for two different resident populations, $\mathbf{p} = (1, 0.1, 0.6, 0.3)$ and $\mathbf{p}' = (1, 0.6, 0.1, 0.3)$. In each case, we record how many mutant strategies it takes until a mutant successfully invades the resident. We report averages over 10^4 iterations. We find that for these two strategies, invasion times are similar. **B,** As a special case, we consider the mutant strategy ALLD, and we plot the players' payoffs as a function of the number of mutants in the population. Also here, $\mathbf{p}$ and $\mathbf{p}'$ are equivalent. **C,D,** We repeat the previous analysis for slightly perturbed strategies, $\mathbf{p} = (0.99, 0.1, 0.6, 0.3)$ and $\mathbf{p}' = (0.99, 0.6, 0.1, 0.3)$. Now the strategies are no longer equivalent. Parameters are the same as in Fig. 4 of the main text.

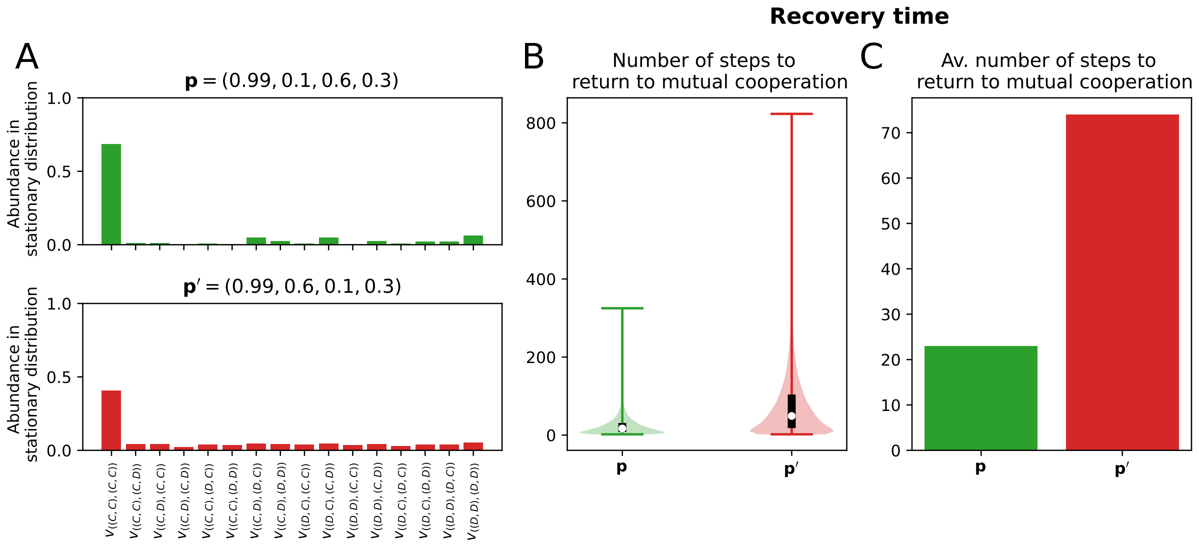

Fig. S5. Game dynamics of reactive-2 strategies when $\mathbf{p}_{\mathbf{CD}}$ and $\mathbf{p}_{\mathbf{DC}}$ are reversed. As in Fig. S4C,D, we consider the two strategies $\mathbf{p} = (0.99, 0.1, 0.6, 0.3)$ and $\mathbf{p}' = (0.99, 0.6, 0.1, 0.3)$. We study games in which a $\mathbf{p}$ player meets another $\mathbf{p}$ player, or in which a $\mathbf{p}'$ player meets another $\mathbf{p}'$ player. **A,** We illustrate the invariant distribution $\mathbf{v}$ of the resulting games. For both strategies, the most abundant state (the most abundant 2-history) is the one in which both players cooperate in both rounds. However, for $\mathbf{p}'$ this state is visited less often. **B,C** To understand the difference, we explore how quickly the two players return to full cooperation, after one of the players defected in the previous round. To this end, we take the state (CC, CD) as the starting history. We use the Axelrod-Python (34) package to simulate the subsequent game dynamics. In each case, we record how many rounds it takes to reach the state (CC, CC). We refer to this number of rounds as the recovery time. Here, we plot the distribution of recovery times and the mean, obtained from $5 \cdot 10^4$ independent realizations. We observe that $\mathbf{p}$ has the shorter recovery time. In panel B, the whiskers represent the minima and maxima, the box extends from the first quartile to the third quartile, and the scatter point in the center shows the mean.

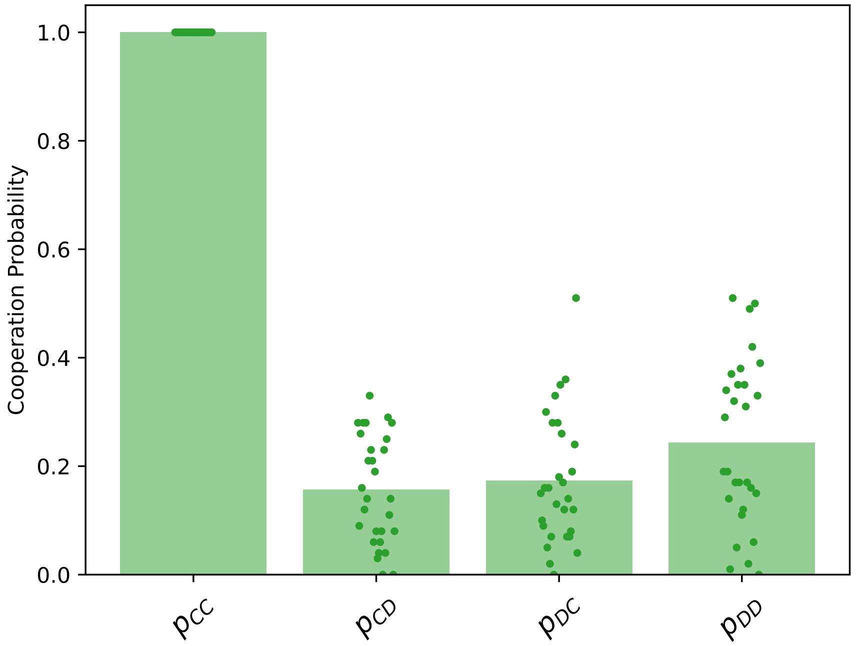

Fig. S6. Most abundant strategies when mutants are sampled from a discretized unit interval. We conducted 30 independent simulations where individuals could adopt strategies from the reactive-2 space. In **Fig. 4** of the main text, mutant strategies were sampled from a uniform distribution over $[0, 1]$, whereas here, they are drawn from a discretized unit interval (e.g., $0.00, 0.01, 0.02, \ldots, 0.99, 1.00$). This change allows probabilities such as 0 and 1, which were previously impossible to sample, to be implemented. We report the strategies of the most abundant residents that were “nice” (i.e., $p_{CC} = 1$). As shown, the asymmetry between p_{cd} and p_{dc} observed when perfect $p_{cc} = 1$ values were unavailable now disappears. Parameters are the same as in Fig. 4 of the main text.

this state more often. For two $\mathbf{p}'$-players, the weights according to the invariant distribution are more spread out across all possible 16 states.

We further explore this difference by exploring how easily players recover from a state in which a single player defected in the last round. That is, we simulate typical game trajectories that start in a state (CC, CD). Then we record how many rounds it takes players to recover a mutual cooperation history (CC, CC). Results are shown in Fig. S5B,C. Again, we find that two $\mathbf{p}$ players recover more quickly from a single defection. On average, it takes them a bit more than 20 rounds, compared to more than 70 rounds for two $\mathbf{p}'$ players. To get a better sense of these recovery results, Table S3 displays the 15 paths that players are most likely to take from state (CC, CD) to state state (CC, CC). According to this table, the top 15 paths among $\mathbf{p}$ players are comparably short (recovery in at most 7 rounds). These 15 paths make up roughly 16.9% of all recoveries. Among $\mathbf{p}'$ players, the respective list features longer paths (up to 12 rounds until recovery), and these 15 paths explain fewer of the total recoveries (only 14.5%).

Lastly, to clearly demonstrate that this asymmetry arises solely because individuals in our simulations cannot implement perfect partner strategies, we ran an evolutionary analysis where mutants are now drawn from a discretized unit interval (e.g., $0.00, 0.01, 0.02, \ldots, 0.99, 1.00$). We ran the analysis as in **Fig. 4** of the main text, specifically for reactive-2 strategies. Namely, we conducted 30 independent simulations. For each simulation, we recorded the most abundant strategy with $p_{CC} = 1$ (the strategy that resisted the most mutants). The results are presented in Fig. S6. As shown, the asymmetry between p_{CD} and p_{DC} is no longer present.

Overall, we obtain the following interpretation for the emerging asymmetry in the evolving reactive-2 strategies. When executed perfectly, partner strategies $\mathbf{p} = (1, 0.1, 0.6, 0.3)$ and $\mathbf{p}' = (1, 0.6, 0.1, 0.3)$ are indeed equivalent with respect to their evolutionary properties. Noisy variants of these strategies, however, are not equivalent. Once we study variants $\mathbf{p} = (0.99, 0.1, 0.6, 0.3)$ and $\mathbf{p}' = (0.99, 0.6, 0.1, 0.3)$, the strategy $\mathbf{p}$ is more robust. This strategy tends to punish a co-player's defection immediately, rather than with one round delay. Our analysis suggests that such strategies are more effective in quickly recovering mutual cooperation if one of the players defected due to stochastic effects.

C. Evolutionary dynamics among memory-n strategies. Given our analytical work, our evolutionary simulations focus on the dynamics among reactive-n strategies. There, we observe that higher memory capacities can favor the evolution of cooperation. At the same time, however, the positive effects of memory length seem to be stronger for reactive-n strategies (when players take into account the entire sequence of moves), compared to reactive-n counting strategies (when players only take into account how often a co-player cooperated). In the following, we ask whether this qualitative result continues to hold in the more general space of memory-n strategies.

To this end, we repeat the evolutionary simulations shown in our main text Fig. 4. However, instead of reactive-n strategies, mutant strategies are now taken from the set of all memory-n strategies, as defined by Eq. (5). Similarly, instead of reactive-n

	$\mathbf{p} = (0.99, 0.1, 0.6, 0.3)$				$\mathbf{p}' = (0.99, 0.6, 0.1, 0.3)$			
Most common paths	Path	Recovery time	Path probability	Frequency of occurrence in 5×10^4 simulations	Path	Recovery time	Path probability	Frequency of occurrence in 5×10^4 simulations
1	CC CC<br>CD CC	2	0.0588	5.9%	CC CC<br>CD CC	2	0.0588	5.9%
2	CC DCC<br>CD CCC	3	0.0318	3.0%	CC CDCC<br>CD CCCC	4	0.0311	3.2%
3	CC DCCC<br>CD CDCC	4	0.0171	1.8%	CC CDCCCC<br>CD CCCDCC	6	0.0165	1.6%
4	CC DCDCC<br>CD CDCCC	5	0.0093	1.0%	CC CDCCCDCC<br>CD CCCDCCCC	8	0.0087	0.9%
5	CC DDDCC<br>CD CDCCC	5	0.0093	0.9%	CC DDCC<br>CD CCCC	4	0.0063	0.6%
6	CC DDCC<br>CD CCCC	4	0.0063	0.6%	CC CDCCCDCCCC<br>CD CCCDCCCDCC	10	0.0046	0.4%
7	CC DDDCC<br>CD CDDCC	5	0.0065	0.6%	CC DDCCCC<br>CD CCCDCC	6	0.0033	0.3%
8	CC DCDCCC<br>CD CDDDCC	6	0.0050	0.5%	CC CDCCCC<br>CD CCDDCC	6	0.0033	0.3%
9	CC DCDCCC<br>CD CDCDCC	6	0.0050	0.5%	CC DDCCCC<br>CD CCDDCC	6	0.0024	0.2%
10	CC DDDDCC<br>CD CDDCCC	6	0.0045	0.4%	CC DCC<br>CD CCC	3	0.0024	0.2%
11	CC DDDCCC<br>CD CDDDCC	6	0.0045	0.4%	CC CDCCCDCCCDCC<br>CD CCCDCCCDCCCC	12	0.0024	0.2%
12	CC DCDDCC<br>CD CDDDCC	6	0.0035	0.4%	CC CDCCCDCC<br>CD CCDDCCCC	8	0.0018	0.2%
13	CC DDCC<br>CD CDCC	4	0.0035	0.3%	CC CDCCDDCC<br>CD CCCDCCCC	8	0.0018	0.2%
14	CC DDDDCC<br>CD CDDDCC	6	0.0032	0.3%	CC DDCCCDCC<br>CD CCCDCCCC	8	0.0018	0.2%
15	CC DCDCDCC<br>CD CDCDCCC	7	0.0020	0.3%	CC DCCCC<br>CD CCDCC	5	0.001	0.1%
Sum				16.9%				14.5%

Table S3. Most likely recovery paths when either two $\mathbf{p}$ players or two $\mathbf{p}'$ players experience a single defection. Based on the simulations in Fig. S5B,C, this table describes typical recovery paths. To obtain this data, we use the Axelrod-Python (34) package to simulate $5 \cdot 10^4$ instances with a starting history (CC, CD). We record the players' subsequent decisions until they reach the state (CC, CC). We refer to such a sequence of decisions from state (CC, CD) to (CC, CC) as a 'path'. Here, we display the 15 paths that were taken most often, separately for each of the two strategies $\mathbf{p}$ and $\mathbf{p}'$. 'Path probability' refers to how likely the respective path is to occur, given an initial state (CC, CD) and the players' conditional cooperation probabilities. 'Frequency' refers to how often we observed the path across all independent realizations. We observe that the most common path is the same for both strategies (here, both players return to full cooperation immediately). However, for the other paths, the recovery times tend to be shorter in the case of $\mathbf{p}$, compared to the case of $\mathbf{p}'$. For $\mathbf{p}'$, two of the most common paths have a recovery time longer than 10. For $\mathbf{p}$, the longest recovery time in this list is 7.

counting strategies, we consider memory-n counting strategies. A memory-n counting strategy can be represented as a vector $\mathbf{m} = (m_{i,j})$, with $0 \le i, j \le n$. The entries $m_{i,j}$ denote a player's cooperation probability in the next round, given the focal player cooperated i times and the co-player cooperated j times during the last n rounds. Memory-1 strategies and memory-1 counting strategies are the same; for $n \ge 2$, memory-n counting strategies are a strict subset of memory-n strategies (similar to the case of reactive strategies).

For memory-n counting strategies, we simulate evolution for $n \in \{1, 2, 3\}$. For memory-n strategies, we simulate $n \in \{1, 2\}$. The space of memory-3 strategies is vast (it corresponds to all 64-dimensional vectors with entries in [0,1]). This space is difficult to sample comprehensively, using simulations with a typical number of mutants (most simulations in the literature consider $10^7 - 10^9$ mutant strategies; the number of pure memory-3 strategies is $2^{64} \approx 2 \cdot 10^{19}$).

Results are shown in Fig. S7, for three different selection strengths, $\beta \in \{0.1, 1, 2\}$. Similar to the case of reactive-n strategies, there is a positive effect of memory on cooperation among the memory-n strategies. However, this time, we also find a notably positive effect of memory among the memory-n counting strategies (even though the effect still seems to be weaker than with memory-n strategies).

D. Evolution of cooperation in games with errors. All simulations so far assume that players execute their actions perfectly. In Fig. S8, we explore the evolution of cooperation when individuals occasionally commit implementation errors, as introduced in Section 3G. The simulations follow the same setup as before; but this time, the players' nominal strategies are subject to an error rate ε. Overall, results are as one may expect: Cooperation is most likely to evolve when the cost of cooperation is sufficiently small, and when errors are comparably rare (Fig. S8A,C). As before, we also observe that individuals are more likely to cooperate when they have longer memory. Again, this effect is particularly pronounced for reactive-n strategies (Fig. S8B,D).

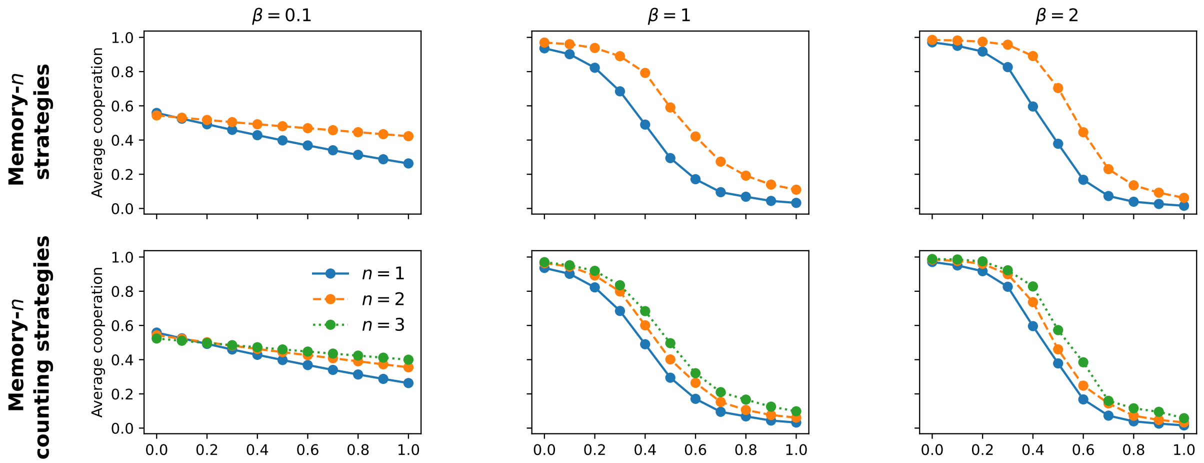

Fig. S7. Dynamics among memory-n strategies. We explore the cooperation rates of memory-n strategies and memory-n counting strategies for different costs and selection strengths. We use the same parameters as in Fig. 4 of the main text: We consider a donation game with $b = 1$, $c = 0.5$, a selection strength $\beta = 1$ and a population size $N = 100$, unless noted otherwise. Simulations are run for 10^7 time steps.

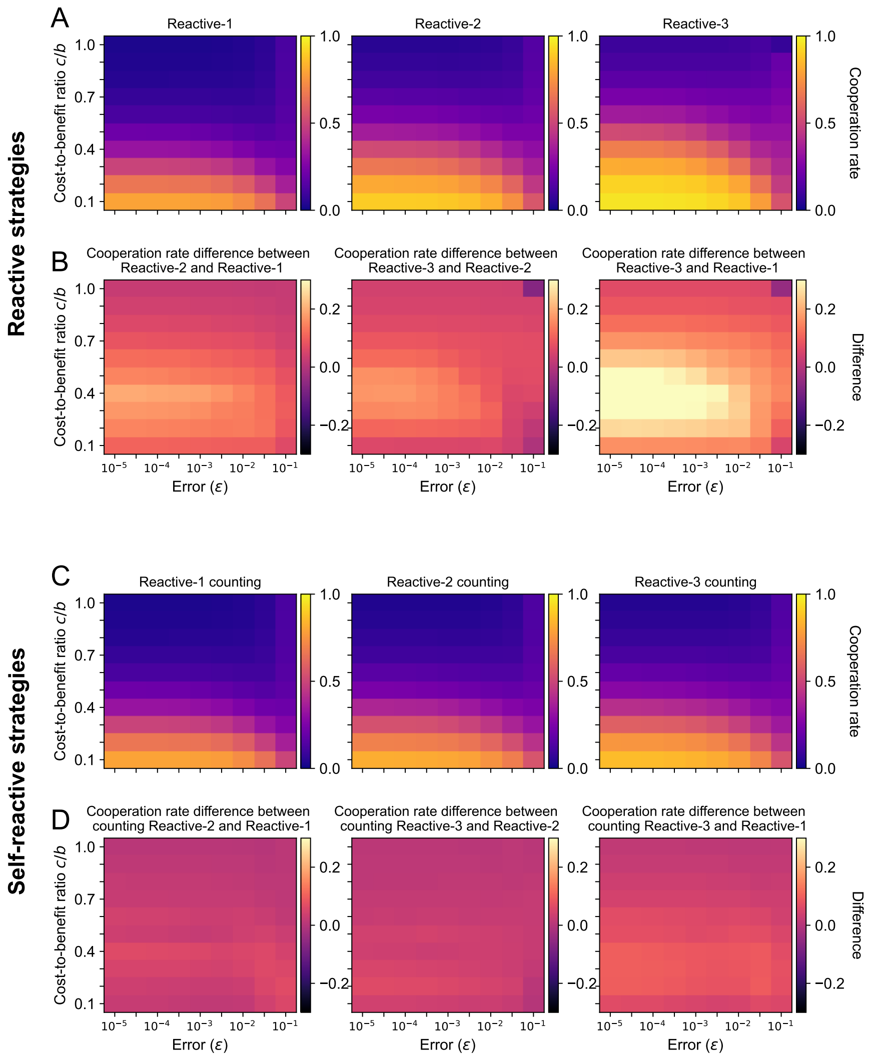

Fig. S8. Evolving cooperation rates in games with implementation errors. We simulate the evolutionary process, this time allowing for implementation errors. Each time a player decides to take an action, with probability ϵ that player implements the opposite action. We calculate the average cooperation rate for different error rates ϵ, different costs c, and for different memory lengths, $n \in \{1, 2, 3\}$. We do this for reactive strategies ($\mathbf{A}$,$\mathbf{B}$) and for reactive counting strategies ($\mathbf{C}$,$\mathbf{D}$). In each case, the upper row depicts the evolving average cooperation rate. In the lower row, we compare these results across different memory lengths. From left to right, we show the differences in cooperation rates between reactive-2 and reactive-1, reactive-3 and reactive-2, and reactive-3 and reactive-1 strategies. Unless noted otherwise, parameters are the same as in Fig. 4 of the main text.

5. Appendix: Proofs

A. Proof of Lemma 1: Akin's lemma.

Proof. The proof is based on a similar argument as the proof of Eq. (18), showing that different ways of calculating payoffs are equivalent. Let us first introduce some notation. Let $\mathbf{m^1}$ be the memory-n strategy of player 1. For $t \geq n$ and the given strategy of player 2, let $\mathbf{v}(t) = (v_\mathbf{h})_{\mathbf{h}\in H}$ be the probability that player 1 observes the n-history $\mathbf{h}$ after players have made their t-th decision. By assumption, we can compute the limiting distribution

$$\mathbf{v} = \lim_{\tau\to\infty} \frac{1}{\tau} \sum_{t=n}^{n+\tau-1} \mathbf{v}(t). \qquad [57]$$

Moreover, let $\rho^i(t)$ be player i's cooperation probability in round t. For $t \geq n+1$, we obtain

$$\rho^1(t) = \big\langle \mathbf{v}(t-1), \mathbf{m^1} \big\rangle = \big\langle \mathbf{v}(t+k-1), \mathbf{m^{k-\mathrm{Rep}}} \big\rangle. \qquad [58]$$

That is, we either need to know how likely each n-history occurred at time $t-1$, and then we compute how likely player 1 is to cooperate in the next round, based on player 1's strategy. Or, we need to know how likely each n-history occurred after round $t+k-1$; and then we compute the correct probability by assuming player 1 cooperates in the next round if and only if the player cooperated k rounds before. Eq. (58) gives us two different ways to compute player 1's average payoff across all rounds,

$$\rho^1 := \lim_{\tau\to\infty} \frac{1}{\tau} \sum_{t=1}^{\tau} \rho^1(t). \qquad [59]$$

The first way is to take

$$\begin{aligned} \rho^1 &= \lim_{\tau\to\infty} \frac{1}{\tau} \sum_{t=1}^{\tau} \rho^i(t) = \lim_{\tau\to\infty} \frac{1}{\tau} \sum_{t=n+1}^{n+\tau} \rho^i(t) \\ &= \lim_{\tau\to\infty} \frac{1}{\tau} \sum_{t=n+1}^{n+\tau} \big\langle \mathbf{v}(t-1), \mathbf{m^1} \big\rangle = \Big\langle \lim_{\tau\to\infty} \frac{1}{\tau} \sum_{t=n+1}^{n+\tau} \mathbf{v}(t-1), \mathbf{m^1} \Big\rangle = \big\langle \mathbf{v}, \mathbf{m^1} \big\rangle. \end{aligned}$$

In particular, because $\big\langle \mathbf{v}, \mathbf{m^1} \big\rangle$ is well-defined, so is the limiting time average ρ^1. The second way is to take

$$\begin{aligned} \rho^1 &= \lim_{\tau\to\infty} \frac{1}{\tau} \sum_{t=1}^{\tau} \rho^i(t) = \lim_{\tau\to\infty} \frac{1}{\tau} \sum_{t=n+1}^{n+\tau} \rho^i(t) \\ &= \lim_{\tau\to\infty} \frac{1}{\tau} \sum_{t=n+1}^{n+\tau} \big\langle \mathbf{v}(t+k-1), \mathbf{m^{k-\mathrm{Rep}}} \big\rangle = \Big\langle \lim_{\tau\to\infty} \frac{1}{\tau} \sum_{t=n+1}^{n+\tau} \mathbf{v}(t+k-1), \mathbf{m^{k-\mathrm{Rep}}} \Big\rangle = \big\langle \mathbf{v}, \mathbf{m^{k-\mathrm{Rep}}} \big\rangle. \end{aligned}$$

We conclude $0 = \rho^1 - \rho^1 = \big\langle \mathbf{v}, \mathbf{m^1} \big\rangle - \big\langle \mathbf{v}, \mathbf{m^{k-\mathrm{Rep}}} \big\rangle = \big\langle \mathbf{v}, \mathbf{m^1} - \mathbf{m^{k-\mathrm{Rep}}} \big\rangle$. □

B. Proof of Lemma 2: Sufficiency of testing self-reactive strategies.

Proof. The proof uses similar arguments as in a study by Park on alternating games *et al* (32). For the given game between player 1 (with arbitrary strategy σ^1) and player 2 (with reactive-n strategy $\mathbf{p^2}$), let $v_\mathbf{h}(t)$ denote the probability to observe an n-history $\mathbf{h}$ at time $t \geq n$. By assumption, the following time averages are well-defined,

$$v_\mathbf{h} := \lim_{\tau\to\infty} \frac{1}{\tau} \sum_{t=n}^{n+\tau-1} v_\mathbf{h}(t) \qquad [60]$$

Moreover, for any $t \geq n$ and $\mathbf{h} \in H$, let $\sigma^1_\mathbf{h}(t)$ denote the conditional probability that player 1 cooperates at time $t+1$, given the n-history after round t is $\mathbf{h}$. Depending on $\big(\sigma^1_\mathbf{h}(t)\big)$ and $\mathbf{v}$, we define an associated self-reactive strategy $\tilde{\mathbf{p}}^\mathbf{1}$ for player 1. For any given history $\mathbf{h^1} \in H^1$, the corresponding probability $\tilde{p}^1_\mathbf{h^1}$ is defined as an implicit solution of the equation

$$\left(\sum_{\mathbf{h^2}\in H^2} v_{(\mathbf{h^1},\mathbf{h^2})} \right) \tilde{p}^1_\mathbf{h^1} = \sum_{\mathbf{h^2}\in H^2} \left(\lim_{\tau\to\infty} \frac{1}{\tau} \sum_{t=n}^{n+\tau-1} v_{(\mathbf{h^1},\mathbf{h^2})}(t) \cdot \sigma^1_{(\mathbf{h^1},\mathbf{h^2})}(t) \right). \qquad [61]$$

Note that for each $\mathbf{h} \in H$, the limit in the bracket on the right hand side exists, for otherwise the limits $v_\mathbf{h}$ according to Eq. (60) would not exist. Also note that if the bracket on the left hand's side is zero, the right hand side must be zero, and $\tilde{p}^1_\mathbf{h^1}$ can be chosen arbitrarily. Only if the bracket on the left hand side is positive, $\tilde{p}^1_\mathbf{h^1}$ is uniquely defined.

We are going to show: If player 1 uses $\tilde{\mathbf{p}}^{\mathbf{1}}$ instead of σ^1, then $\mathbf{v}$ defined by Eq. (60) is an invariant distribution of the corresponding transition matrix M defined by Eq. (12) (hence it is also the limiting distribution of the resulting game if the first n moves are chosen accordingly). For simplicity, we show the required relationship $\mathbf{v}=\mathbf{v}M$ for one of the 2^{2^n} equations. For the one equation we show, we consider the history according to which everyone fully cooperates, $\mathbf{h_C}=(\mathbf{h^1_C},\mathbf{h^2_C})$. For an arbitrary n-history $\mathbf{h}^i=(a^i_{-n},\ldots,a^i_{-i})$, we say the n-history $\tilde{\mathbf{h}}^{\mathbf{i}}=(\tilde{a}^i_{-n},\ldots,\tilde{a}^i_{-1})$ *is a possible successor of* $\mathbf{h}$ if $\tilde{a}^i_{-t}=a^i_{-t+1}$ for $t\in\{2,\ldots,n\}$. To indicate successorship, we define a function $e_{\mathbf{h},\tilde{\mathbf{h}}}$ that is one if $\tilde{\mathbf{h}}$ is a possible successor of $\mathbf{h}$, and zero otherwise. By definition of $v_{\mathbf{h}}(t)$, $\sigma^1_{\mathbf{h}}(t)$, and $p^2_{\mathbf{h}}(t)$, we obtain for $t\geq n$

$$v_{(\mathbf{h^1_C},\mathbf{h^2_C})}(t+1)=\sum_{\mathbf{h^1}\in H^1}\sum_{\mathbf{h^2}\in H^2} v_{(\mathbf{h^1},\mathbf{h^2})}(t)\cdot\sigma^1_{(\mathbf{h^1},\mathbf{h^2})}(t)\cdot p^2_{\mathbf{h^1}}\cdot e_{\mathbf{h^1},\mathbf{h^1_C}}\cdot e_{\mathbf{h^2},\mathbf{h^2_C}}. \qquad [62]$$

If we sum up this equation from time $t=n$ to $t=n+\tau-1$, divide by τ, and rearrange the terms, we obtain

$$\frac{1}{\tau}\sum_{t=n}^{n+\tau-1} v_{(\mathbf{h^1_C},\mathbf{h^2_C})}(t+1)=\sum_{\mathbf{h^1}\in H^1}\sum_{\mathbf{h^2}\in H^2}\left(\frac{1}{\tau}\sum_{t=n}^{n+\tau-1} v_{(\mathbf{h^1},\mathbf{h^2})}(t)\cdot\sigma^1_{(\mathbf{h^1},\mathbf{h^2})}(t)\right)\cdot p^2_{\mathbf{h^1}}\cdot e_{\mathbf{h^1},\mathbf{h^1_C}}\cdot e_{\mathbf{h^2},\mathbf{h^2_C}}. \qquad [63]$$

Taking the limit $\tau\to\infty$, and taking into account the relationships Eq. (60) and Eq. (61), this simplifies to

$$v_{(\mathbf{h^1_C},\mathbf{h^2_C})}=\sum_{\mathbf{h^1}\in H^1}\sum_{\mathbf{h^2}\in H^2} v_{(\mathbf{h^1},\mathbf{h^2})}\cdot\left(\tilde{p}^1_{\mathbf{h^1}}\,e_{\mathbf{h^1},\mathbf{h^1_C}}\right)\cdot\left(p^2_{\mathbf{h^1}}\,e_{\mathbf{h^2},\mathbf{h^2_C}}\right). \qquad [64]$$

By using the definition of transition probabilities in Eq. (12), this expression further simplifies to

$$v_{\mathbf{h_C}}=\sum_{\mathbf{h}} v_{\mathbf{h}}\cdot M_{\mathbf{h},\mathbf{h_C}} \qquad [65]$$

That is, out of the 2^{2n} individual equations in the linear system $\mathbf{v}=\mathbf{v}M$, we have verified the equation for the probability to observe full cooperation $\mathbf{h_C}$ after one round. All other equations follow analogously. □

C. Proof of Theorem 1: Sufficiency of pure self-reactive strategies. By Lemma 2, there exists a best response to $\mathbf{p}$ within the self-reactive n strategies. It remains to show that this best response $\tilde{\mathbf{p}}$ can be chosen to be pure. The proof follows from a series of auxiliary results. The first such result uses an insight by Press & Dyson (12). They showed that given the transition matrix of a game among two memory-1 players, one can compute the players' payoffs by considering determinants of certain associated matrices. Herein, we apply their method to the transition matrix $\tilde{M}=(\tilde{M}_{\mathbf{h},\mathbf{h'}})$ according to Eq. Eq. (27) for a given self-reactive strategy $\tilde{\mathbf{p}}\in\mathcal{S}_n$. For some fixed n-history $\mathbf{h}'$, we define an associated matrix $\tilde{M}_{\mathbf{h}'}$ that one obtains from $\tilde{M}$ with the following two steps:

1. Subtract the $2^n\times 2^n$ identity matrix I from $\tilde{M}$.
2. In the resulting matrix, replace the last column by a column that only contains zeros, except for the row corresponding to the history $\mathbf{h}'$, for which the entry is one.

These matrices $\tilde{M}_{\mathbf{h}'}$ can be used to compute the invariant distribution of the original matrix $\tilde{M}$ as follows.

Auxiliary result 1: Let $\tilde{\mathbf{p}}\in\mathcal{S}_n$ be such that its transition matrix $\tilde{M}$ according to Eq. Eq. (27) has a unique invariant distribution $\tilde{\mathbf{v}}=(\tilde{v}_{\mathbf{h^1}})_{\mathbf{h^1}\in H^1}$. Then for all $\mathbf{h}'\in H^1$ we have

$$\tilde{v}_{\mathbf{h}'}=\frac{\det(\tilde{M}_{\mathbf{h}'})}{\sum_{\mathbf{h^1}\in H^1}\det(\tilde{M}_{\mathbf{h^1}})}. \qquad [66]$$

Proof of Auxiliary result 1. The result follows from Press & Dyson's formula for the dot product of the invariant distribution $\tilde{\mathbf{v}}$ with an arbitrary vector $\mathbf{f}$, by taking the vector $\mathbf{f}$ to be the unit vector with only the entry for history $\mathbf{h}'$ being one. □

Based on this first auxiliary result, we have an explicit representation of the payoff function $\pi^1(\tilde{\mathbf{p}},\mathbf{p})$ that describes the payoff of a self-reactive player with strategy $\tilde{\mathbf{p}}$ against a reactive player with strategy $\mathbf{p}$. Specifically, by plugging Eq. Eq. (66) into Eq. (28), we obtain

$$\pi^1(\tilde{\mathbf{p}},\mathbf{p})=\frac{\sum_{\mathbf{h^1}\in H^1}\det(\tilde{M}_{\mathbf{h^1}})\Big(\tilde{\mathbf{p}}_{\mathbf{h^1}}\mathbf{p}_{\mathbf{h^1}}\cdot R+\tilde{\mathbf{p}}_{\mathbf{h^1}}(1-\mathbf{p}_{\mathbf{h^1}})\cdot S+(1-\tilde{\mathbf{p}}_{\mathbf{h^1}})\mathbf{p}_{\mathbf{h^1}}\cdot T+(1-\tilde{\mathbf{p}}_{\mathbf{h^1}})(1-\mathbf{p}_{\mathbf{h^1}})\cdot P\Big)}{\sum_{\mathbf{h^1}\in H^1}\det(\tilde{M}_{\mathbf{h^1}})}. \qquad [67]$$

For our purposes, the following properties of this payoff function will be important.

Auxiliary Result 2: On its domain, the payoff function $\pi^1(\tilde{\mathbf{p}},\mathbf{p})$ is a bounded rational function, and both its numerator and denominator are linear in each entry $\tilde{p}_{\mathbf{h^i}}$, for all $\mathbf{h^i}\in H^i$.

Proof of Auxiliary Result 2. By its definition, each $\det(\tilde{M}_{\mathbf{h^i}})$ is a polynomial. Moreover, because for each history $\mathbf{h}'$, the cooperation probability $\tilde{p}_{\mathbf{h}'}$ only appears in a single row of $\tilde{M}_{\mathbf{h^i}}$ (and there it appears linearly), it also appears linearly in $\det(\tilde{M}_{\mathbf{h^i}})$. Finally, we note that $\det(\tilde{M}_{\mathbf{h^i}})$ does not depend on $\tilde{p}_{\mathbf{h^i}}$. To see this, we can compute $\det(\tilde{M}_{\mathbf{h^i}})$ using Laplace expansion along the last column. As a result, we obtain that this determinant is up to its sign equal to the determinant of the matrix one obtains from $\tilde{M}_{\mathbf{h^i}}$ by deleting the last column, and the row $\mathbf{h^i}$ (which is the only row of $\tilde{M}_{\mathbf{h^i}}$ that contains $\tilde{p}_{\mathbf{h^i}}$).

Finally, we note that the payoff function is bounded, because as an average payoff per round, payoffs need to be between T and S. Taken together, these observations imply the result for $\pi^1(\tilde{\mathbf{p}}, \mathbf{p})$. ☐

The following result describes a useful property of bounded linear rational functions.

Auxiliary Result 3: Suppose $g, h : [0,1]^k \to \mathbb{R}$ and suppose both $g(\mathbf{x})$ and $h(\mathbf{x})$ are linear in each component of $\mathbf{x} = (x_1, \ldots, x_k)$. Moreover, suppose $f := g/h$ is bounded on $[0,1]^k$. For a given $\mathbf{x}$ and $j \in \{1, \ldots, k\}$, we define an associated function $f_{\mathbf{x},j} : [-x_j, 1-x_j] \to \mathbb{R}$ by only varying the j-th component, $f_{\mathbf{x},j}(t) = f(x_1, \ldots, x_j + t, \ldots, x_k)$. Then for all $\mathbf{x} \in [0,1]^k$ and j, the function $f_{\mathbf{x},j}(t)$ is either monotonically increasing, monotonically decreasing, or constant.

Proof of Auxiliary Result 3. Let $g(\mathbf{x}) := a_0 + a_1 x_1 + \ldots + a_k x_k$ and $h(\mathbf{x}) := b_0 + b_1 x_1 + \ldots + b_k x_k$, and consider some arbitrary but fixed $\mathbf{x} \in [0,1]^k$ and j. We compute

$$f'_{\mathbf{x},j}(t) = \frac{\partial}{\partial t} f(x_1, \ldots, x_j + t, \ldots, x_k) = \frac{a_j \left(\sum_{i \neq j} b_i x_i\right) - b_j \left(\sum_{i \neq j} a_i x_i\right)}{\left(b_0 + b_1 x_1 + \ldots + b_j (x_j + t) + \ldots + b_k x_k\right)^2}. \qquad [68]$$

Because f is bounded on the entire domain, the denominator in this expression for $f'_{\mathbf{x},j}(t)$ is strictly positive. Moreover, we note that the numerator is independent of t. Thus, depending on the sign of the numerator, $f'_{\mathbf{x},j}(t)$ is either monotonically increasing, monotonically decreasing, or constant. ☐

After these preparations, we are ready to prove the main result.

Proof of Theorem 1. For a given reactive strategy $\mathbf{p} \in \mathcal{R}_n$, let the self-reactive $\tilde{\mathbf{p}} \in \mathcal{S}_n$ be a best response. Suppose there is some history $\mathbf{h}'$ such that $0 < \tilde{p}_{\mathbf{h}} < 1$. It follows from the Auxiliary Results 2 and 3 that $\pi^1(\tilde{\mathbf{p}}, \mathbf{p})$ is either monotonically increasing, monotonically decreasing, or constant in $\tilde{p}_{\mathbf{h}'}$. If it was increasing or decreasing, we end up with a contradiction, because no local improvement should be possible for a best response. Therefore, $\pi^1(\tilde{\mathbf{p}}, \mathbf{p})$ must be independent of $\tilde{p}_{\mathbf{h}'}$, and hence we can set $\tilde{p}_{\mathbf{h}'} = 0$ or $\tilde{p}_{\mathbf{h}'} = 1$ without changing $\pi^1(\tilde{\mathbf{p}}, \mathbf{p})$. By iteratively applying this reasoning to all histories $\mathbf{h}$ for which $0 < \tilde{p}_{\mathbf{h}} < 1$, we obtain the desired result. ☐

D. Proof of Theorem 2: Reactive-2 partner strategies in the donation game.

Proof. Given that player 1 uses a nice reactive-2 strategy $\mathbf{p} = (1, p_{CD}, p_{DC}, p_{DD})$, the claim is true if and only if it is true for all deviation towards the sixteen pure self-reactive-2 strategies $\tilde{\mathbf{p}} \in \{0,1\}^{16}$. In the following, we enumerate these sixteen strategies, $\{\tilde{\mathbf{p}}_\mathbf{0}, \ldots, \tilde{\mathbf{p}}_\mathbf{15}\}$, by interpreting them as binary numbers,

$$\tilde{\mathbf{p}} = (\tilde{p}_{CC}, \tilde{p}_{CD}, \tilde{p}_{DC}, \tilde{p}_{DD}) \;\mapsto\; \tilde{p}_{CC} \cdot 2^3 + \tilde{p}_{CD} \cdot 2^2 + \tilde{p}_{DC} \cdot 2^1 + p_{DD} \cdot 2^0. \qquad [69]$$

In particular, `ALLD` $= (0,0,0,0)$ is mapped to the number $j = 0$, and `ALLC` $= (1,1,1,1)$ is mapped to $j = 15$. The possible payoffs against the reactive strategy $\mathbf{p}$ can be computed by Eq. (28), which yields

$$\begin{aligned}
\pi^1(\tilde{\mathbf{p}}_\mathbf{j}, \mathbf{p}) &= p_{DD} \cdot b && \text{for } j \in \{0,2,4,6,8,10,12,14\} \\
\pi^1(\tilde{\mathbf{p}}_\mathbf{j}, \mathbf{p}) &= \frac{p_{CD} + p_{DC} + p_{DD}}{3} \cdot b - \frac{1}{3} \cdot c && \text{for } j \in \{1,9\} \\
\pi^1(\tilde{\mathbf{p}}_\mathbf{j}, \mathbf{p}) &= \frac{1 + p_{CD} + p_{DC} + p_{DD}}{4} \cdot b - \frac{1}{2} \cdot c && \text{for } j \in \{3\} \\
\pi^1(\tilde{\mathbf{p}}_\mathbf{j}, \mathbf{p}) &= \frac{p_{CD} + p_{DC}}{2} \cdot b - \frac{1}{2} \cdot c && \text{for } j \in \{4,5,12,13\} \\
\pi^1(\tilde{\mathbf{p}}_\mathbf{j}, \mathbf{p}) &= \frac{1 + p_{CD} + p_{DC}}{3} \cdot b - \frac{2}{3} \cdot c && \text{for } j \in \{6,7\} \\
\pi^1(\tilde{\mathbf{p}}_\mathbf{j}, \mathbf{p}) &= b - c && \text{for } j \in \{8,9,10,11,12,13,14,15\}
\end{aligned}$$

In this list, some strategy indices j appear multiple times. Those instances correspond to strategies that have multiple invariant distributions (such as the strategy 1-round repeat, with $j = 10$). For those strategies, we have computed the payoffs for all possible initial n-histories. Requiring the payoffs in this list to be at most the mutual cooperation payoff $b - c$, we get the following unique conditions,

$$p_{DD} \leq 1 - \frac{c}{b}, \qquad \frac{p_{CD} + p_{DC}}{2} \leq 1 - \frac{1}{2}\frac{c}{b}, \qquad \frac{p_{CD} + p_{DC} + p_{DD}}{3} \leq 1 - \frac{2}{3}\frac{c}{b}.$$

Because the last condition is implied by the first two, we end up with the conditions in Eq. (35). □

E. Proof of Theorem 3: Reactive-3 partner strategies in the donation game.

Proof. The proof is similar to the previous one. Again, enumerating the 256 pure self-reactive 3 strategies $\tilde{\mathbf{p}}$ by interpreting the strategy as a binary number, we obtain the following payoffs.

$$\pi^1(\tilde{\mathbf{p}}_\mathbf{j},\mathbf{p}) = b\ p_{DDD} \qquad \text{for } j\in\{0,2,4,6,\dots,250,252,254\}$$

$$\pi^1(\tilde{\mathbf{p}}_\mathbf{j},\mathbf{p}) = \frac{p_{CDD}+p_{DCD}+p_{DDC}+p_{DDD}}{4}\,b-\frac{1}{4}\,c \qquad \text{for } j\in\{1,9,33,41,65,73,97,105,129,137,161,169,193,201,225,233\}$$

$$\pi^1(\tilde{\mathbf{p}}_\mathbf{j},\mathbf{p}) = \frac{p_{CCD}+p_{CDD}+p_{DCC}+p_{DDC}+p_{DDD}}{5}\,b-\frac{2}{5}\,c \qquad \text{for } j\in\{3,7,35,39,131,135,163,167\}$$

$$\pi^1(\tilde{\mathbf{p}}_\mathbf{j},\mathbf{p}) = \frac{p_{CDC}+p_{DCD}}{2}\,b-\frac{1}{2}\,c \qquad \text{for } j\in\{4-7,12-15,20-23,28-31,68-71,76-79,84-87,92-95,132-135,140-143,148-151,156-159,196-199,204-207,212-215,220-223\}$$

$$\pi^1(\tilde{\mathbf{p}}_\mathbf{j},\mathbf{p}) = \frac{1+p_{CCD}+p_{CDD}+p_{DCC}+p_{DDC}+p_{DDD}}{6}\,b-\frac{1}{2}\,c \qquad \text{for } j\in\{11,15,43,47\}$$

$$\pi^1(\tilde{\mathbf{p}}_\mathbf{j},\mathbf{p}) = \frac{p_{CDD}+p_{DCD}+p_{DDC}}{3}\,b-\frac{1}{3}\,c \qquad \text{for } j\in\{16,17,24,25,48,49,56,57,80,81,88,89,112,113,120,121,144,145,152,153,176,177,184,185,208,209,216,217,240,241,248,249\}$$

$$\pi^1(\tilde{\mathbf{p}}_\mathbf{j},\mathbf{p}) = \frac{p_{CCD}+p_{CDD}+p_{DCC}+p_{DDC}}{4}\,b-\frac{1}{2}\,c \qquad \text{for } j\in\{18,19,22,23,50,51,54,55,146,147,150,151,178,179,182,183\}$$

$$\pi^1(\tilde{\mathbf{p}}_\mathbf{j},\mathbf{p}) = \frac{1+p_{CCD}+p_{CDD}+p_{DCC}+p_{DDC}}{5}\,b-\frac{3}{5}\,c \qquad \text{for } j\in\{26,27,30,31,58,59,62,63\}$$

$$\pi^1(\tilde{\mathbf{p}}_\mathbf{j},\mathbf{p}) = \frac{p_{CCD}+p_{CDC}+p_{CDD}+p_{DCC}+p_{DCD}+p_{DDC}+p_{DDD}}{7}\,b-\frac{3}{7}\,c \qquad \text{for } j\in\{37,67,165,195\}$$

$$\pi^1(\tilde{\mathbf{p}}_\mathbf{j},\mathbf{p}) = \frac{1+p_{CCD}+p_{CDC}+p_{CDD}+p_{DCC}+p_{DCD}+p_{DDC}+p_{DDD}}{8}\,b-\frac{1}{2}\,c \qquad \text{for } j\in\{45,75\}$$

$$\pi^1(\tilde{\mathbf{p}}_\mathbf{j},\mathbf{p}) = \frac{p_{CCD}+p_{CDC}+p_{CDD}+p_{DCC}+p_{DCD}+p_{DDC}}{6}\,b-\frac{1}{2}\,c \qquad \text{for } j\in\{52,53,82,83,180,181,210,211\}$$

$$\pi^1(\tilde{\mathbf{p}}_\mathbf{j},\mathbf{p}) = \frac{1+p_{CCD}+p_{CDC}+p_{CDD}+p_{DCC}+p_{DCD}+p_{DDC}}{7}\,b-\frac{4}{7}\,c \qquad \text{for } j\in\{60,61,90,91\}$$

$$\pi^1(\tilde{\mathbf{p}}_\mathbf{j},\mathbf{p}) = \frac{p_{CCD}+p_{CDC}+p_{DCC}}{3}\,b-\frac{2}{3}\,c \qquad \text{for } j\in\{96-103,112-119,224-231,240-247\}$$

$$\pi^1(\tilde{\mathbf{p}}_\mathbf{j},\mathbf{p}) = \frac{1+p_{CCD}+p_{CDC}+p_{DCC}}{4}\,b-\frac{3}{4}\,c \qquad \text{for } j\in\{104-111,120-127\}$$

$$\pi^1(\tilde{\mathbf{p}}_\mathbf{j},\mathbf{p}) = b-c \qquad \text{for } j\in\{128,129,130,\dots,255\}$$

Requiring these payoffs to be at most equal to the mutual cooperation payoff $b-c$ gives

$$p_{DDD}\le 1-\frac{c}{b},\qquad \frac{p_{CDC}+p_{DCD}}{2}\le 1-\frac{1}{2}\cdot\frac{c}{b},\qquad \frac{p_{CDD}+p_{DCD}+p_{DDC}}{3}\le 1-\frac{2}{3}\cdot\frac{c}{b},$$

$$\frac{p_{CCD}+p_{CDC}+p_{DCC}}{3}\le 1-\frac{1}{3}\cdot\frac{c}{b},\qquad \frac{p_{CCD}+p_{CDD}+p_{DCC}+p_{DDC}}{4}\le 1-\frac{1}{2}\cdot\frac{c}{b},$$

$$\frac{p_{CDD}+p_{DCD}+p_{DDC}+p_{DDD}}{4}\le 1-\frac{3}{4}\cdot\frac{c}{b},\qquad \frac{p_{CCD}+p_{CDC}+p_{CDD}+p_{DCC}+p_{DCD}+p_{DDC}+p_{DDD}}{7}\le 1-\frac{4}{7}\cdot\frac{c}{b},$$

$$\frac{p_{CCD}+p_{CDD}+p_{DCC}+p_{DDC}+p_{DDD}}{5}\le 1-\frac{3}{5}\cdot\frac{c}{b},\qquad \frac{p_{CCD}+p_{CDC}+p_{CDD}+p_{DCC}+p_{DCD}+p_{DDC}}{6}\le 1-\frac{1}{2}\cdot\frac{c}{b}.$$

The statement follows by noting that the five conditions in the first two rows imply the four other conditions. □

F. Proof of Theorem 4: Reactive-n counting strategies in the donation game. Before we go into the details of the proof, we first start with two useful observations.

1. Assume player 1 adopts a given self-reactive strategy $\tilde{\mathbf{p}}$ and player 2 adopts the reactive-n strategy $\mathbf{r}=(r_k)_{k\in\{n,\dots,0\}}$. For the resulting game, suppose $\mathbf{v}$ is the limiting distribution according to Eq. (14). Then it is useful to express $\mathbf{v}$ in terms of what the counting player can remember. To this end, let H^1_k be the set of n-histories according to which player 1 has cooperated exactly k times,

$$H^1_k=\left\{\mathbf{h^1}\in H^1\;\middle|\;|\mathbf{h^1}|=k\right\}. \qquad [70]$$

 Nikoleta E. Glynatsi, Ethan Akin, Martin A. Nowak, Christian Hilbe

Accordingly, let $\mathbf{u}=(u_k)_{k\in\{0,\ldots,n\}}$ be the distribution that summarizes how often, on average, player 1 cooperates j times during n consecutive rounds,

$$u_k^1 = \sum_{\mathbf{h^1}\in H_k^1} v_{\mathbf{h^1}}. \qquad [71]$$

In particular, the entries of $\mathbf{u}$ are normalized,

$$\sum_{k=0}^{n} u_k^1 = 1. \qquad [72]$$

Moreover, the average cooperation rate of the two players can be written as

$$\rho^1 = \sum_{k=0}^{n} \frac{k}{n}\, u_k^1 \qquad \text{and} \qquad \rho^2 = \sum_{k=0}^{n} r_k\, u_k^1. \qquad [73]$$

Because payoffs in the donation game only depend on the players' average cooperation rates (but not on the timing of cooperation), we conclude that player 1's payoff is

$$\pi^1(\tilde{\mathbf{p}},\mathbf{r}) = \sum_{k=0}^{n} (r_k\, b - \frac{k}{n}\, c)\, u_k^1. \qquad [74]$$

2. There is a set of strategies for which payoffs are particularly easy to compute. We refer to them as simple periodic strategies, σ_k with $k\in\{0,\ldots,n\}$. A player with strategy σ_k cooperates in round t if and only if

$$t-1 \mod n < k. \qquad [75]$$

That is, such a player cooperates in the first k rounds, then defects for $n-k$ rounds, then cooperates for another k rounds, only to defect in the $n-k$ subsequent rounds, etc. Such strategies are interesting for two reasons. First, they all can be interpreted as a round-n repeat strategy $\tilde{\mathbf{p}}^{\mathbf{n}-\mathrm{Rep}}$, as defined by Eq. (11). During the initial n rounds, they cooperate according to Eq. (75); thereafter, they simply repeat whatever they have done n rounds ago. Second, players with strategy σ_k always act in such a way that according to any resulting n-history, they have cooperated exactly k times during the last n rounds. As a result, if player 1 adopts such a strategy in a donation game against a player with a reactive-n counting strategy $\mathbf{r}$, then player 1's average payoff is

$$\pi^1(\sigma_k,\mathbf{r}) = r_k\, b - \frac{k}{n}\, c. \qquad [76]$$

After these observations, we are ready for the actual proof.

Proof of Theorem 4.

(⇒) Suppose the reactive-n counting strategy $\mathbf{r}$ is a partner. Because it is nice, it cooperates against an unconditional cooperator, and hence $r_n=1$. Because it is a Nash equilibrium, player 1 must not have an incentive to deviate towards any of the simple periodic strategies σ_k. By Eq. (76), this means that for all $k\in\{0,\ldots,n\}$ we have

$$r_k\, b - \frac{k}{n}\, c \le b-c. \qquad [77]$$

These conditions are equivalent to $r_{n-k} \le 1-\frac{k}{n}\,\frac{c}{b}$, the inequalities in Eq. (39).

(⇐) Because $\mathbf{r}$ is nice, $r_n=1$. The proof is now by contradiction; suppose the conditions in Eq. (39) hold, but $\mathbf{r}$ is not a Nash equilibrium. Then there needs to be some self-reactive $\tilde{\mathbf{p}}$ such that $\pi^1(\tilde{\mathbf{p}},\mathbf{r}) > b-c$. It follows that

$$\begin{aligned}
0 \quad &< \quad \pi^1(\tilde{\mathbf{p}},\mathbf{r}) - (b-c)\\
&\overset{\text{Eq. (72),Eq. (74)}}{=} \sum_{k=0}^{n}(r_k\, b - \frac{k}{n}\, c)\, u_k^1 \;-\; \sum_{k=0}^{n}(b-c)u_k^1\\
&= \quad (r_n-1)b\, u_n + \sum_{k=0}^{n-1}\Big((r_k-1)b + \frac{n-k}{n}c\Big)\, u_k^1\\
&= \quad b\cdot\sum_{k=1}^{n}\underbrace{\Big(r_{n-k} - \big(1-\frac{k}{n}\frac{c}{b}\big)\Big)}_{\le\, 0 \text{ by Eq. (39)}} u_{n-k}^1 \le 0.
\end{aligned} \qquad [78]$$

We end up with $0<0$, a contradiction.

□

G. Proof of Theorem 5: Reactive-2 partner strategies in the prisoner's dilemma.

Proof. The proof is analogous to the proof of Theorem 2 for the donation game. For the general prisoner's dilemma, the payoffs of the 16 pure self-reactive-2 strategies are

$$\begin{aligned}
\pi^1(\tilde{\mathbf{p}}_\mathbf{j}, \mathbf{p}) &= P(1-p_{DD}) + T p_{DD} && \text{for } i \in \{0, 2, 4, 6, 8, 10, 12, 14\}\\
\pi^1(\tilde{\mathbf{p}}_\mathbf{j}, \mathbf{p}) &= \frac{R p_{DD} + S(1-p_{DD}) + T(p_{CD}+p_{DC}) + P(2-p_{CD}-p_{DC})}{3} && \text{for } i \in \{1, 9\}\\
\pi^1(\tilde{\mathbf{p}}_\mathbf{j}, \mathbf{p}) &= \frac{R(p_{DC}+p_{DD}) + S(2-p_{DC}-p_{DD}) + T(p_{CD}+1) + P(1-p_{CD})}{4} && \text{for } i \in \{3\}\\
\pi^1(\tilde{\mathbf{p}}_\mathbf{j}, \mathbf{p}) &= \frac{R p_{CD} + S(1-p_{CD}) + T p_{DC} + P(1-p_{DC})}{2} && \text{for } i \in \{4, 5, 12, 13\}\\
\pi^1(\tilde{\mathbf{p}}_\mathbf{j}, \mathbf{p}) &= \frac{R(p_{CD}+p_{DC}) + S(2-p_{CD}-p_{DC}) + T}{3} && \text{for } i \in \{6, 7\}\\
\pi^1(\tilde{\mathbf{p}}_\mathbf{j}, \mathbf{p}) &= R && \text{for } i \in \{8, 9, 10, 11, 12, 13, 14, 15\}
\end{aligned}$$

By requiring these expressions to be at most equal to R, we obtain

$$\begin{aligned}
(T-P)\, p_{DD} &\leq R-P,\\
(R-S)\,(p_{CD}+p_{DC}) &\leq 3R-2S-T,\\
(T-P)\, p_{DC} + (R-S)\, p_{CD} &\leq 2R-S-P,\\
(T-P)\,(p_{CD}+p_{DC}) + (R-S)\, p_{DD} &\leq 3R-S-2\,P,\\
(T-P)\, p_{CD} + (R-S)\,(p_{CD}+p_{DD}) &\leq 4\,R-2\,S-P-T.
\end{aligned}$$

□

H. Proof of Theorem 6: Reactive-3 partner strategies in the prisoner's dilemma. Again, we compute payoffs for all 256 self-reactive-3 strategies. The expressions are given below,

$$\pi^1(\tilde{\mathbf{p}}_\mathbf{j},\mathbf{p}) = \frac{(T-P)\left(p_{CDD}+p_{DCD}+p_{DDC}\right)+3\,P+(R-S)\,p_{DDD}+S}{4} \qquad \text{for } j\in\{1,9,33,41,65,73,97,105,129,137,161,169,193,201,225,233\}$$

$$\pi^1(\tilde{\mathbf{p}}_\mathbf{j},\mathbf{p}) = \frac{(T-P)\,p_{CDC}+P+(R-S)\,p_{DCD}+S}{2} \qquad \text{for } j\in\{4-7,12-15,20-23,28-31,68-71,76-79,84-87,92-95,132-135,140-143,148-151,156-159,196-199,204-207,212-215,220-223\}$$

$$\pi^1(\tilde{\mathbf{p}}_\mathbf{j},\mathbf{p}) = -P\left(p_{DDD}-1\right)+T\,p_{DDD} \qquad \text{for } j\in\{0,2,4,\ldots,252,254\}$$

$$\pi^1(\tilde{\mathbf{p}}_\mathbf{j},\mathbf{p}) = \frac{(T-P)\,(p_{CCD}+p_{CDD}+p_{DDC})+3\,P+(R-S)\,(p_{CDC}+p_{DCC}+p_{DCD}+p_{DDD})+4\,S+T}{8} \qquad \text{for } j\in\{45\}$$

$$\pi^1(\tilde{\mathbf{p}}_\mathbf{j},\mathbf{p}) = \frac{(T-P)\,p_{DCC}+P+(R-S)\,(p_{CDC}+p_{CCD})+2\,S}{3} \qquad \text{for } j\in\{96-103,112-119,224-231,240-247\}$$

$$\pi^1(\tilde{\mathbf{p}}_\mathbf{j},\mathbf{p}) = \frac{(T-P)\,(p_{CCD}+p_{DCC}+p_{DDC})+3\,P+(R-S)\,(p_{CDC}+p_{CDD}+p_{DCD})+3\,S}{6} \qquad \text{for } j\in\{52,53,180,181\}$$

$$\pi^1(\tilde{\mathbf{p}}_\mathbf{j},\mathbf{p}) = \frac{(T-P)\,(p_{CCD}+p_{DDC})+2\,P+T+(R-S)\,(p_{CDC}+p_{CDD}+p_{DCC}+p_{DCD})+4\,S}{7} \qquad \text{for } j\in\{60,61\}$$

$$\pi^1(\tilde{\mathbf{p}}_\mathbf{j},\mathbf{p}) = \frac{(T-P)\,(p_{CCD}+p_{CDD}+p_{DCC})+3\,P+(R-S)\,(p_{DDC}+p_{DDD})+2\,S}{5} \qquad \text{for } j\in\{3,7,35,39,131,135,163,167\}$$

$$\pi^1(\tilde{\mathbf{p}}_\mathbf{j},\mathbf{p}) = \frac{(T-P)\,(p_{DCD}+p_{DDC})+2\,P+(R-S)\,p_{CDD}+S}{3} \qquad \text{for } j\in\{16,17,24,25,48,49,56,57,80,81,88,89,112,113,120,121,144,145,152,153,176,177,184,185,208,209,216,217,240,241,248,249\}$$

$$\pi^1(\tilde{\mathbf{p}}_\mathbf{j},\mathbf{p}) = R \qquad \text{for } j\in\{128,129,\ldots,255\}$$

$$\pi^1(\tilde{\mathbf{p}}_\mathbf{j},\mathbf{p}) = \frac{(T-P)\,p_{CCD}+P+T+(R-S)\,(p_{CDD}+p_{DCC}+p_{DDC})+3S}{5} \qquad \text{for } j\in\{26,27,30,31,58,59,62,63\}$$

$$\pi^1(\tilde{\mathbf{p}}_\mathbf{j},\mathbf{p}) = \frac{(T-P)\,(p_{CCD}+p_{DCC})+2\,P+(R-S)\,(p_{CDD}+p_{DDC})+2\,S}{4} \qquad \text{for } j\in\{18,19,22,23,50,51,54,55,146,147,150,151,178,179,182,183\}$$

$$\pi^1(\tilde{\mathbf{p}}_\mathbf{j},\mathbf{p}) = \frac{(T-P)\,(p_{CDC}+p_{DCD})+2\,P+T+(R-S)\,(p_{CCD}+p_{CDD}+p_{DCC}+p_{DDC})+4\,S}{7} \qquad \text{for } j\in\{90,91\}$$

$$\pi^1(\tilde{\mathbf{p}}_\mathbf{j},\mathbf{p}) = \frac{(T-P)\,(p_{CDC}+p_{CDD}+p_{DCD})+3\,P+T+(R-S)\,(p_{CCD}+p_{DCC}+p_{DDC}+p_{DDD})+4\,S}{8} \qquad \text{for } j\in\{75\}$$

$$\pi^1(\tilde{\mathbf{p}}_\mathbf{j},\mathbf{p}) = \frac{(T-P)\,(p_{CDC}+p_{DCC}+p_{DCD})+3\,P+(R-S)\,(p_{CCD}+p_{CDD}+p_{DDC})+3\,S}{6} \qquad \text{for } j\in\{82,83,210,211\}$$

$$\pi^1(\tilde{\mathbf{p}}_\mathbf{j},\mathbf{p}) = \frac{(T-P)\,(p_{CCD}+p_{CDD}+p_{DCC}+p_{DDC})+4\,P+(R-S)\,(p_{CDC}+p_{DCD}+p_{DDD})+3\,S}{7} \qquad \text{for } j\in\{37,165\}$$

$$\pi^1(\tilde{\mathbf{p}}_\mathbf{j},\mathbf{p}) = \frac{T+(R-S)\,(p_{CCD}+p_{CDC}+p_{DCC})+3\,S}{4} \qquad \text{for } j\in\{104-111,120-127\}$$

$$\pi^1(\tilde{\mathbf{p}}_\mathbf{j},\mathbf{p}) = \frac{(T-P)\,(p_{CCD}+p_{CDD})+2\,P+T+(R-S)\,(p_{DCC}+p_{DDC}+p_{DDD})+3\,S}{6} \qquad \text{for } j\in\{11,15,43,47\}$$

$$\pi^1(\tilde{\mathbf{p}}_\mathbf{j},\mathbf{p}) = \frac{(T-p)\,(p_{CDC}+p_{CDD}+p_{DCC}+p_{DCD})+4\,P+(R-S)\,(p_{CCD}+p_{DDC}+p_{DDD})+3\,S}{7} \qquad \text{for } j\in\{67,195\}$$

By requiring the above expressions to be smaller than or equal to R, we obtain the inequalities in Table S1.

I. Proof of Theorem 7: Reactive-n defector strategies in the donation game.

Proof. The proof is analogous to the corresponding proof for partner strategies. Again, we enumerate the respective self-reactive strategies $\tilde{\mathbf{p}}$ that we need to check, by interpreting the strategy as a binary number.

1. If the co-player adopts a reactive-1 strategy, the possible payoffs of a pure self-reactive-1 strategy are

$$\begin{aligned}
\pi^1(\tilde{\mathbf{p}}_\mathbf{j},\mathbf{p}) &= 0 && \text{for } j\in\{0,2\} \\
\pi^1(\tilde{\mathbf{p}}_\mathbf{j},\mathbf{p}) &= \frac{b\cdot p_C-c}{p_C+1} && \text{for } j\in\{1\} \\
\pi^1(\tilde{\mathbf{p}}_\mathbf{j},\mathbf{p}) &= b\cdot p_C-c && \text{for } j\in\{3\}
\end{aligned}$$

   In particular, by requiring that each of these payoffs is at most the mutual defection payoff of zero, we get $p_C\leq c/b$. The requirement $p_D=0$ follows from the requirement that a defector is never the first to cooperate.

2. For $n=2$, the possible payoffs of a pure self-reactive strategy are

$$\begin{aligned}
\pi^1(\tilde{\mathbf{p}}_\mathbf{j},\mathbf{p}) &= 0 && \text{for } j\in\{0,2,4,6,8,10,12,14\}\\
\pi^1(\tilde{\mathbf{p}}_\mathbf{j},\mathbf{p}) &= \frac{p_{CD}+p_{DC}}{3}\,b-\frac{c}{3} && \text{for } j\in\{1,9\}\\
\pi^1(\tilde{\mathbf{p}}_\mathbf{j},\mathbf{p}) &= \frac{p_{CC}+p_{CD}+p_{DC}}{4}\,b-\frac{c}{2} && \text{for } j\in\{3\}\\
\pi^1(\tilde{\mathbf{p}}_\mathbf{j},\mathbf{p}) &= \frac{p_{CD}+p_{DC}}{2}\,b-\frac{c}{2} && \text{for } j\in\{4,5,12,13\}\\
\pi^1(\tilde{\mathbf{p}}_\mathbf{j},\mathbf{p}) &= \frac{p_{CC}+p_{CD}+p_{DC}}{3}\,b-\frac{2c}{3} && \text{for } j\in\{6,7\}\\
\pi^1(\tilde{\mathbf{p}}_\mathbf{j},\mathbf{p}) &= p_{CC}\,b-c && \text{for } j\in\{8,9,10,11,12,13,14,15\}
\end{aligned}$$

By imposing the requirement that each of these payoffs is at most zero, we get the following unique conditions,

$$p_{CC}\le\frac{c}{b},\qquad \frac{p_{CD}+p_{DC}}{2}\le\frac{1}{2}\cdot\frac{c}{b},\qquad \frac{p_{CD}+p_{DC}+p_{CC}}{3}\le\frac{2}{3}\cdot\frac{c}{b}.$$

Because the last condition is implied by the first two, we end up with the inequalities in Eq. (41).

3. For $n=3$, the possible payoffs of a pure self-reactive strategy are

$$\begin{aligned}
&\pi^1(\tilde{\mathbf{p}}_\mathbf{j},\mathbf{p})=0 && \text{for } j\in\{0,2,4,6,\ldots,250,252,254\}\\
&\pi^1(\tilde{\mathbf{p}}_\mathbf{j},\mathbf{p})=\frac{p_{CDD}+p_{DCD}+p_{DDC}}{4}\,b-\frac{1}{4}\,c && \text{for } j\in\{1,9,33,41,65,73,97,105,129,137,161,\\
& && \quad 169,193,201,225,233\}\\
&\pi^1(\tilde{\mathbf{p}}_\mathbf{j},\mathbf{p})=\frac{p_{CCD}+p_{CDD}+p_{DCC}+p_{DDC}}{5}\,b-\frac{2}{5}\,c && \text{for } j\in\{3,7,35,39,131,135,163,167\}\\
&\pi^1(\tilde{\mathbf{p}}_\mathbf{j},\mathbf{p})=\frac{p_{CDC}+p_{DCD}}{2}\,b-\frac{1}{2}\,c && \text{for } j\in\{4-7,12-15,20-23,28-31,68-71,\\
& && \quad 76-79,84-87,92-95,132-135,\\
& && \quad 140-143,148-151,156-159,\\
& && \quad 196-199,204-207,212-215,220-223\}\\
&\pi^1(\tilde{\mathbf{p}}_\mathbf{j},\mathbf{p})=\frac{p_{CCC}+p_{CCD}+p_{CDD}+p_{DCC}+p_{DDC}}{6}\,b-\frac{1}{2}\,c && \text{for } j\in\{11,15,43,47\}\\
&\pi^1(\tilde{\mathbf{p}}_\mathbf{j},\mathbf{p})=\frac{p_{CDD}+p_{DCD}+p_{DDC}}{3}\,b-\frac{1}{3}\,c && \text{for } j\in\{16,17,24,25,48,49,56,57,80,81,88,\\
& && \quad 89,112,113,120,121,144,145,152,153,\\
& && \quad 176,177,184,185,208,209,216,217,\\
& && \quad 240,241,248,249\}\\
&\pi^1(\tilde{\mathbf{p}}_\mathbf{j},\mathbf{p})=\frac{p_{CCD}+p_{CDD}+p_{DCC}+p_{DDC}}{4}\,b-\frac{1}{2}\,c && \text{for } j\in\{18,19,22,23,50,51,54,55,146,147,\\
& && \quad 150,151,178,179,182,183\}\\
&\pi^1(\tilde{\mathbf{p}}_\mathbf{j},\mathbf{p})=\frac{p_{CCC}+p_{CCD}+p_{CDD}+p_{DCC}+p_{DDC}}{5}\,b-\frac{3}{5}\,c && \text{for } j\in\{26,27,30,31,58,59,62,63\}\\
&\pi^1(\tilde{\mathbf{p}}_\mathbf{j},\mathbf{p})=\frac{p_{CCD}+p_{CDC}+p_{CDD}+p_{DCC}+p_{DCD}+p_{DDC}}{7}\,b-\frac{3}{7}\,c && \text{for } j\in\{37,67,165,195\}\\
&\pi^1(\tilde{\mathbf{p}}_\mathbf{j},\mathbf{p})=\frac{p_{CCC}+p_{CCD}+p_{CDC}+p_{CDD}+p_{DCC}+p_{DCD}+p_{DDC}}{8}\,b-\frac{1}{2}\,c && \text{for } j\in\{45,75\}\\
&\pi^1(\tilde{\mathbf{p}}_\mathbf{j},\mathbf{p})=\frac{p_{CCD}+p_{CDC}+p_{CDD}+p_{DCC}+p_{DCD}+p_{DDC}}{6}\,b-\frac{1}{2}\,c && \text{for } j\in\{52,53,82,83,180,181,210,211\}\\
&\pi^1(\tilde{\mathbf{p}}_\mathbf{j},\mathbf{p})=\frac{p_{CCC}+p_{CCD}+p_{CDC}+p_{CDD}+p_{DCC}+p_{DCD}+p_{DDC}}{7}\,b-\frac{4}{7}\,c && \text{for } j\in\{60,61,90,91\}\\
&\pi^1(\tilde{\mathbf{p}}_\mathbf{j},\mathbf{p})=\frac{p_{CCD}+p_{CDC}+p_{DCC}}{3}\,b-\frac{2}{3}\,c && \text{for } j\in\{96-103,112-119,224-231,240-247\}\\
&\pi^1(\tilde{\mathbf{p}}_\mathbf{j},\mathbf{p})=\frac{p_{CCC}+p_{CCD}+p_{CDC}+p_{DCC}}{4}\,b-\frac{3}{4}\,c && \text{for } j\in\{104-111,120-127\}\\
&\pi^1(\tilde{\mathbf{p}}_\mathbf{j},\mathbf{p})=p_{CCC}\,b-c && \text{for } j\in\{128,129,130,\ldots,255\}
\end{aligned}$$

Requiring these payoffs to be at most equal to zero gives

$$p_{CCC}\le\frac{c}{b},\qquad \frac{p_{CDC}+p_{DCD}}{2}\le\frac{1}{2}\cdot\frac{c}{b},\qquad \frac{p_{CDD}+p_{DCD}+p_{DDC}}{3}\le\frac{1}{3}\cdot\frac{c}{b},$$

$$\frac{p_{CCD}+p_{CDC}+p_{DCC}}{3}\le\frac{2}{3}\cdot\frac{c}{b},\qquad \frac{p_{CCD}+p_{CDD}+p_{DCC}+p_{DDC}}{4}\le\frac{1}{2}\cdot\frac{c}{b},$$

$$\frac{p_{CCC}+p_{CCD}+p_{CDC}+p_{DCC}}{4}\le\frac{3}{4}\cdot\frac{c}{b},\qquad \frac{p_{CCC}+p_{CCD}+p_{CDD}+p_{DCC}+p_{DDC}}{6}\le\frac{1}{2}\cdot\frac{c}{b},$$

$$\frac{p_{CCD}+p_{CDC}+p_{CDD}+p_{DCC}+p_{DCD}+p_{DDC}}{6}\le\frac{1}{2}\cdot\frac{c}{b},\qquad \frac{p_{CCC}+p_{CCD}+p_{CDC}+p_{CDD}+p_{DCC}+p_{DCD}+p_{DDC}}{8}\le\frac{1}{2}\cdot\frac{c}{b}.$$

The statement follows by noting that the five conditions in the first two rows imply the four other conditions.

□

6. Supplementary References

1. R Axelrod, *The evolution of cooperation.* (Basic Books, New York, NY), (1984).
2. MA Nowak, *Evolutionary dynamics.* (Harvard University Press, Cambridge MA), (2006).
3. K Sigmund, *The calculus of selfishness.* (Princeton University Press), (2010).
4. C Hilbe, K Chatterjee, MA Nowak, Partners and rivals in direct reciprocity. *Nat. Hum. Behav.* **2**, 469–477 (2018).
5. N Glynatsi, V Knight, A bibliometric study of research topics, collaboration and centrality in the field of the Iterated Prisoner's Dilemma. *Humanit. Soc. Sci. Commun.* **8**, 45 (2021).
6. S Do Yi, SK Baek, JK Choi, Combination with anti-tit-for-tat remedies problems of tit-for-tat. *J. Theor. Biol.* **412**, 1–7 (2017).
7. Y Murase, SK Baek, Five rules for friendly rivalry in direct reciprocity. *Sci. Reports* **10**, 16904 (2020).
8. Y Murase, SK Baek, Grouping promotes both partnership and rivalry with long memory in direct reciprocity. *PLoS Comput. Biol.* **19**, e1011228 (2023).
9. C Hilbe, LA Martinez-Vaquero, K Chatterjee, MA Nowak, Memory-n strategies of direct reciprocity. *Proc. Natl. Acad. Sci.* **114**, 4715–4720 (2017).
10. M Nowak, K Sigmund, A strategy of win-stay, lose-shift that outperforms tit-for-tat in the prisoner's dilemma game. *Nature* **364**, 56–58 (1993).
11. J Li, et al., Evolution of cooperation through cumulative reciprocity. *Nat. Comput. Sci.* **2**, 677–686 (2022).
12. WH Press, FJ Dyson, Iterated prisoner's dilemma contains strategies that dominate any evolutionary opponent. *Proc. Natl. Acad. Sci.* **109**, 10409–10413 (2012).
13. M Ueda, Memory-two zero-determinant strategies in repeated games. *Royal Soc. Open Sci.* **8**, 202186 (2021).
14. M Ueda, Controlling conditional expectations by zero-determinant strategies. *Oper. Res. Forum* **3**, 48 (2022).
15. A McAvoy, MA Nowak, Reactive learning strategies for iterated games. *Proc. Royal Soc. A* **475**, 20180819 (2019).
16. AJ Stewart, JB Plotkin, Small groups and long memories promote cooperation. *Sci. Reports* **6**, 1–11 (2016).
17. AJ Stewart, JB Plotkin, Collapse of cooperation in evolving games. *Proc. Natl. Acad. Sci. USA* **111**, 17558 – 17563 (2014).
18. R Axelrod, WD Hamilton, The evolution of cooperation. *Science* **211**, 1390–1396 (1981).
19. MA Nowak, K Sigmund, Tit for tat in heterogeneous populations. *Nature* **355**, 250–253 (1992).
20. P Molander, The optimal level of generosity in a selfish, uncertain environment. *J. Confl. Resolut.* **29**, 611–618 (1985).
21. E Akin, The iterated prisoner's dilemma: good strategies and their dynamics in *Ergodic Theory, Advances in Dynamical Systems.* (de Gruyter Berlin), pp. 77–107 (2016).
22. C Hilbe, B Wu, A Traulsen, MA Nowak, Cooperation and control in multiplayer social dilemmas. *Proc. Natl. Acad. Sci. USA* **111**, 16425–16430 (2014).
23. C Hilbe, A Traulsen, K Sigmund, Partners or rivals? Strategies for the iterated prisoner's dilemma. *Games economic behavior* **92**, 41–52 (2015).
24. MC Boerlijst, MA Nowak, K Sigmund, Equal pay for all prisoners. *Am. Math. Mon.* **104**, 303–307 (1997).
25. D Fudenberg, DG Rand, A Dreber, Slow to anger and fast to forgive: Cooperation in an uncertain world. *Am. Econ. Rev.* **102**, 720–749 (2012).
26. AA Arechar, A Dreber, D Fudenberg, DG Rand, "I'm just a soul whose intentions are good": The role of communication in noisy repeated games. *Games Econ. Behav.* **104**, 726–743 (2017).
27. R Axelrod, D Dion, The further evolution of cooperation. *Science* **242**, 1385–1390 (1988).
28. J Wu, R Axelrod, How to cope with noise in the iterated prisoner's dilemma. *J. Confl. Resolut.* **39**, 183–189 (1995).
29. R Boyd, J Lorberbaum, No pure strategy is evolutionary stable in the iterated prisoner's dilemma game. *Nature* **327**, 58–59 (1987).
30. R Boyd, Mistakes allow evolutionary stability in the repeated Prisoner's Dilemma game. *J. Theor. Biol.* **136**, 47–56 (1989).
31. J García, M van Veelen, In and out of equilibrium I: Evolution of strategies in repeated games with discounting. *J. Econ. Theory* **161**, 161–189 (2016).
32. PS Park, MA Nowak, C Hilbe, Cooperation in alternating interactions with memory constraints. *Nat. Commun.* **13**, 737 (2022).
33. R Levinsky, A Neyman, M Zeleny, Should I remember more than you? On the best response to factor-based strategies. *Int. J. Game Theory* **49**, 1105–1124 (2020).
34. V Knight, et al., Axelrod-python/axelrod: v4.12.0 (2023).
